# Diversity, Bioactivity Profiling and Untargeted Metabolomics of the Cultivable Gut Microbiota of *Ciona intestinalis*

**DOI:** 10.3390/md19010006

**Published:** 2020-12-24

**Authors:** Caroline Utermann, Vivien A. Echelmeyer, Ernest Oppong-Danquah, Martina Blümel, Deniz Tasdemir

**Affiliations:** 1 GEOMAR Centre for Marine Biotechnology (GEOMAR-Biotech), Research Unit Marine Natural Products Chemistry, GEOMAR Helmholtz Centre for Ocean Research Kiel, Am Kiel-Kanal 44, 24106 Kiel, Germany; cutermann@geomar.de (C.U.); vivienechelmeyer@web.de (V.A.E.); eoppong-danquah@geomar.de (E.O.-D.); mbluemel@geomar.de (M.B.); 2 Faculty of Mathematics and Natural Sciences, Kiel University, Christian-Albrechts-Platz 4, 24118 Kiel, Germany

**Keywords:** tunicate, *Ciona intestinalis*, gut-associated microbiota, marine natural products, antimicrobial activity, anticancer activity, untargeted metabolomics, feature-based molecular networking, in silico MS/MS-based dereplication

## Abstract

It is widely accepted that the commensal gut microbiota contributes to the health and well-being of its host. The solitary tunicate *Ciona intestinalis* emerges as a model organism for studying host–microbe interactions taking place in the gut, however, the potential of its gut-associated microbiota for marine biodiscovery remains unexploited. In this study, we set out to investigate the diversity, chemical space, and pharmacological potential of the gut-associated microbiota of *C. intestinalis* collected from the Baltic and North Seas. In a culture-based approach, we isolated 61 bacterial and 40 fungal strains affiliated to 33 different microbial genera, indicating a rich and diverse gut microbiota dominated by Gammaproteobacteria. In vitro screening of the crude microbial extracts indicated their antibacterial (64% of extracts), anticancer (22%), and/or antifungal (11%) potential. Nine microbial crude extracts were prioritized for in-depth metabolome mining by a bioactivity- and chemical diversity-based selection procedure. UPLC-MS/MS-based metabolomics combining automated (feature-based molecular networking and in silico dereplication) and manual approaches significantly improved the annotation rates. A high chemical diversity was detected where peptides and polyketides were the predominant classes. Many compounds remained unknown, including two putatively novel lipopeptides produced by a *Trichoderma* sp. strain. This is the first study assessing the chemical and pharmacological profile of the cultivable gut microbiota of *C. intestinalis*.

## 1. Introduction

The animal gut is one of the most densely colonized microbial habitats representing a highly specialized internal ecosystem [1,2,3]. The commensal gut microbiota is known for contributing to the host’s health and homeostasis by assisting, e.g., chemical defense, immunity, metabolic capacity, and digestion [1,2,3]. For instance, vertebrate-associated gut bacteria provide “colonization resistance” through, e.g., short-chain fatty acids to inhibit proliferation of pathogenic microorganisms such as *Salmonella enterica* [3,4]. Commensal gut bacteria also induce immune reactions by producing antimicrobial peptides or aid nutrient uptake by breaking down complex polysaccharides [3,4]. In the marine world, cultivable gut-associated bacteria of farmed fish, such as *Vibrio* sp., have been reported to inhibit common aquaculture pathogens, e.g., *Vibrio anguillarum* and *Pasteurella piscicida* [5,6]. The current evidence suggests that *Vibrio* spp. residing in the alimentary tract of the alga-feeding sea urchin *Strongylocentrotus* spp. promote the animal´s digestion by breaking down large algal polysaccharides such as alginates [7]. 

Marine microorganisms represent unparalleled resources for biodiscovery of compounds with great pharmaceutical application potential [8,9]. The majority of marine natural products (MNPs) discovered between 2014 and 2018 originate from bacteria or fungi [9]. Microorganisms associated with invertebrate hosts such as sponges and tunicates are promising resources for marine biodiscovery [8,10,11,12]. For example, ascidian-associated bacteria are prolific producers of antibiotics and anticancer drug leads [11,13] including the anticancer drug Yondelis® produced by a gammaproteobacterial symbiont of the colonial sea squirt *Ecteinascidia turbinata* [14]. However, the gut microbiota of marine sessile animals has rarely been studied. The few available examples include the ascomycete fungi *Aspergillus* sp. and *Letendraea* sp., which were isolated from the gut of marine crustaceans [15,16,17]. They yielded novel cytotoxic aspochalazines [15,16] and the anti-inflammatory polyketide phomopsiketone D [17], rendering the gut-associated microbiota of marine invertebrates as a valuable and underexploited source for MNP biodiscovery.

Host-associated microbial communities can be analyzed by culture-dependent and -independent methods. Culture-based studies capture only a small fraction of the actual microbiota (~0.001–1%), often select for easily culturable microorganisms, and, therefore, do not adequately reflect the microbial diversity [18,19]. In contrast, culture-independent approaches such as metagenomics and amplicon sequencing allow comprehensive description of the microbiota of interest, although the large majority of the detected microorganisms remains uncultivable [19]. Hence, comparison of the microbial diversity obtained by both methodologies often reveals a huge discrepancy [20], mainly due to the large fraction of uncultured microorganisms and the strong impact of the applied cultivation media on the culture-dependent microbial diversity [18,19]. Despite recent advances enabling, e.g., the access to compounds of yet uncultivable microorganisms via heterologous expression, marine biodiscovery studies still largely apply cultivation-dependent methods, mainly due to their high efficiency for bioactivity screening [21].

For decades, the sea vase *Ciona* spp. (chordate subphylum Tunicata) has served as a model organism for developmental biology, evolution, and chordate immunity [22,23]. Recently, *C. intestinalis* and *C. robusta* were introduced as model organisms for studying host–microbe interactions in animal gastrointestinal tracts, because they feature a compartmentalized gut resembling that of the vertebrates [2,23,24]. The gut of the filter feeder is in constant contact with millions of microbial cells posing a great challenge for the tunicate; on the one hand it must defend against pathogenic microorganisms, but at the same time allow colonization of commensals [2,25]. Initial studies have reported a few gut-associated Gammaproteobacteria (e.g., *Shewanella* sp. and *Vibrio* sp. [23,26,27]) and Ascomycota (e.g., *Penicillium* sp. and *Trichoderma* sp. [28]) from *C. intestinalis* and *C. robusta*. In line with this, amplicon sequencing of the bacterial community associated with *C. intestinalis* and *C. robusta* described a specific and diverse gut community dominated by Gammaproteobacteria [29], while culture-dependent and -independent studies on the tunic-associated microbiota of *Ciona* spp. revealed comparably high abundance of Alphaproteobacteria [30,31]. However, no information exists on chemical composition or biological activities of the gut-associated microbial community of *C. intestinalis*. This fact prompted us to isolate and study cultivable bacteria and fungi associated with the gut of *C. intestinalis* to explore their chemical machinery. Dissected guts of *C. intestinalis* sampled at two sites in Germany (Helgoland, North Sea and Kiel Fjord, Baltic Sea) yielded 61 bacterial and 40 fungal isolates. An initial bioactivity screening (antimicrobial and anticancer) and chemical profiling of the crude extracts of these microorganisms allowed selection of nine microbial extracts for LC-MS/MS-based untargeted metabolomics employing feature-based molecular networking (FBMN) [32], in silico [33] and manual dereplication tools. This study enabled us to prioritize two bacterial and three fungal strains for purification and characterization of their bioactive constituents in future.

## 2. Results

### 2.1. Cultivable Fraction of the Gut Microbiota of C. intestinalis

**Figure 1:**
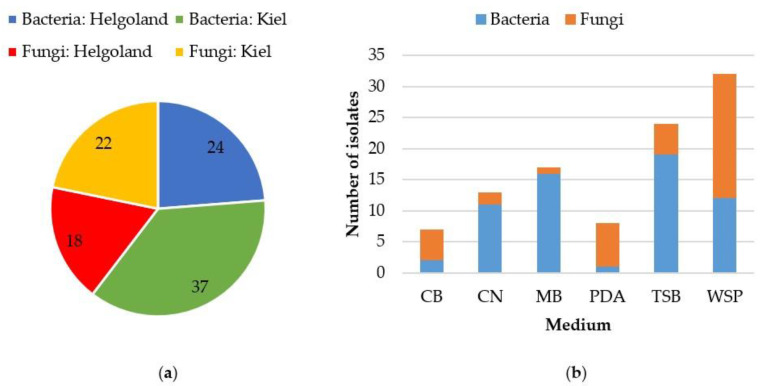
Distribution of bacterial (*n* = 61) and fungal (*n* = 40) isolates deriving from the gut of *Ciona intestinalis* sampled in the North (Helgoland) and Baltic Seas (Kiel Fjord). Numbers of bacterial and fungal isolates are displayed for (**a**) the two different sampling sites and (**b**) with respect to the used isolation media. CB/CN: *C. intestinalis* media adjusted to Baltic (CB) or North Sea (CN) salinity; MB: Marine Broth; PDA: potato dextrose agar; TSB: trypticase soy broth; WSP: modified Wickerham medium.

Application of six different cultivation media led to 61 bacterial and 40 fungal isolates from the gut of *C. intestinalis* sampled in Helgoland (H) and Kiel Fjord (K; Figure 1a, Table S1). The number of bacterial isolates was much higher in the Baltic Sea samples than in the North Sea samples (H: 24, K: 37). However, samples from Kiel Fjord (Baltic Sea) and the North Sea island Helgoland yielded similar numbers of fungal isolates (H: 18, K: 22). As expected, the applied isolation media had a remarkable influence on the number of isolated microorganisms (Figure 1b). Isolation on modified Wickerham medium (WSP) yielded the highest number of isolates (32, compared to other media: 7–24 isolates). Bacterial isolates derived mainly from Marine Broth (MB; 26%) and tryptic soy broth (TSB; 31%) media, while gut-associated fungal strains were mainly obtained from WSP medium (20 isolates; other media: 1–7 fungal isolates). Similar to our previous study that reported the tunic-associated microbiota of *C. intestinalis* [31], we used media resembling the natural habitat of the isolates, i.e., media adjusted to Baltic (CB) or North Sea (CN) salinity containing *C. intestinalis* powder. They yielded many microbial strains (*n* = 20), and the microbial genera *Acrostalagmus*, *Arthopyrenia*, *Cordyceps,* and *Sporosarcina* were exclusively isolated from these media. Accordingly, the *C. intestinalis* media CB and CN proved valuable for isolating a diverse tunicate-associated microbiota. 

**Figure 2:**
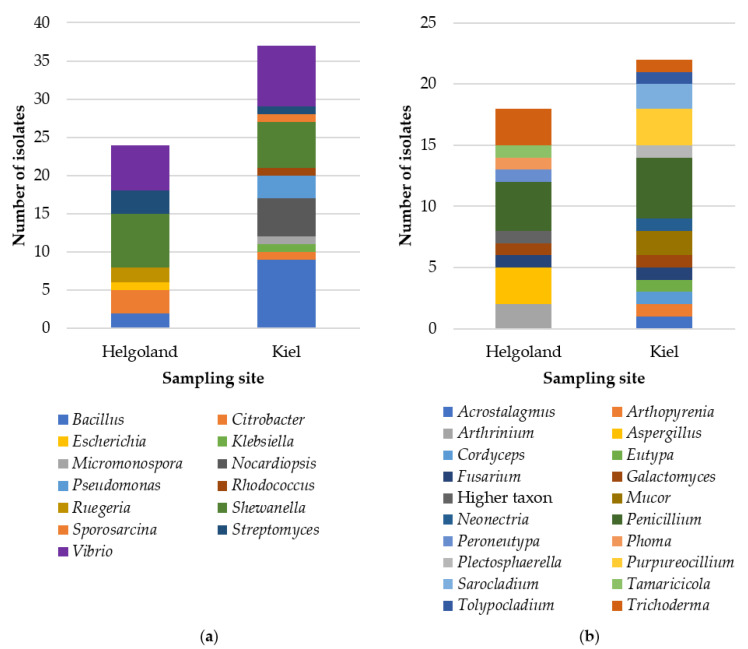
Diversity of (**a**) bacterial and (**b**) fungal isolates associated with the gut of *C. intestinalis* at genus level. The designation “Higher taxon” refers to isolate CHG49 (only identified to family level, Pleosporaceae).

Compositionally, the cultivable microbiota was affiliated to three bacterial (Actinobacteria, Firmicutes, Proteobacteria) and two fungal phyla (Ascomycota, Mucoromycota; Table S1). Sanger sequencing allowed identification of all but one isolate to species (31 isolates) or genus (69 isolates) level. The gut-associated bacterial community was dominated by *Shewanella* sp. (H: 7 isolates, K: 6 isolates) and *Vibrio* sp. (H: 6 isolates, K: 8 isolates; Figure 2a). Out of 13 bacterial genera, six were exclusively found in Baltic *C. intestinalis* gut samples (*Klebsiella*, *Micromonospora*, *Nocardiopsis*, *Pseudomonas*, *Rhodococcus*, and *Sporosarcina*), while only two were exclusive to Helgoland (*Escherichia* and *Ruegeria*). Moreover, *Bacillus* sp. showed higher abundance in Kiel (9 isolates) compared to samples from Helgoland (2 isolates). *Penicillium* was the predominant fungal genus with four (from H) or five isolates (from K), respectively (Figure 2b). Out of 20 detected fungal genera, only *Fusarium*, *Galactomyces*, *Penicillium*, and *Trichoderma* were common to both locations indicating a differential fungal diversity of the gut of *C. intestinalis* collected from Helgoland and Kiel Fjord. Helgoland-exclusive fungal genera included, e.g., *Arthrinium* sp. (2 isolates) and *Aspergillus* sp. (3 isolates). The gut of Baltic *C. intestinalis* delivered 10 exclusive fungal genera such as *Mucor* sp. (2 isolates), *Purpureocillium* sp. (3 isolates), and *Sarocladium* sp. (2 isolates).

### 2.2. Biological Activities of Gut-Derived Microbial Extracts

To assess the biotechnological potential of the gut-associated microbiota, bacterial isolates were cultured on the agar media glucose–yeast–malt (GYM) and Marine Broth (MB), while fungi were grown on solid casamino acids–glucose (CAG) and potato dextrose agar (PDA) media. Extracts received identification codes referring to the host organism *C. intestinalis* (C), the sampling site (H or K), the origin of the microbial isolates (gut, G), the respective strain number, and cultivation medium (CAG, GYM, MB or PDA). For example, CHG2-MB is the Marine Broth extract of strain 2 that was isolated from the gut of *C. intestinalis* sampled in Helgoland.

**Figure 3:**
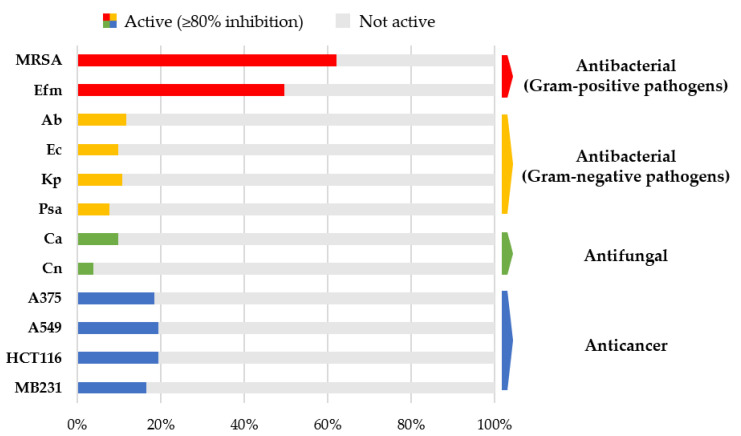
Antimicrobial and anticancer activities of 103 microbial crude extracts. Extracts were classified as active when showing inhibitory activity ≥80% at a test concentration of 100 µg/mL. Activities were determined against Gram-positive bacterial pathogens (red; MRSA: methicillin-resistant *Staphylococcus aureus*, Efm: *Enterococcus faecium*), Gram-negative bacterial pathogens (yellow; Ab: *Acinetobacter baumannii*, Ec: *Escherichia coli*, Kp: *Klebsiella pneumoniae*, Psa: *Pseudomonas aeruginosa*), fungal pathogens (green; Ca: *Candida albicans*, Cn: *Cryptococcus neoformans*) and cancer cell lines (blue; A375: malignant melanoma, A549: lung carcinoma, HCT116: colon cancer, MB231: breast cancer).

In vitro bioactivities were determined for 103 microbial crude extracts against eight human microbial pathogens including the ESKAPE panel (see Section 4.4.), *Candida albicans,* and *Cryptococcus neoformans,* and against four cancer cell lines. A total of 68 extracts (reflecting 66%) were active at a bioactivity threshold of ≥80% inhibition (= highly active, test concentration of 100 µg/mL) in at least one assay (Table S2). Most frequently, activity was observed against the Gram-positive bacterial pathogens methicillin-resistant *Staphylococcus aureus* (MRSA; 62%) and *Enterococcus faecium* (50%; Figure 3, Table S2). Notably, twelve crude extracts derived from the fungi *Acrostalagmus luteoalbus* (CKG66-CAG), *Galactomyces candidum* (CKG25-CAG, -PDA), *Penicillium* sp. (CHG25-CAG, -PDA, CHG35-CAG, -PDA, CKG23-CAG, -PDA, CKG63-PDA), and Pleosporaceae sp. (CHG49-CAG, -PDA) inhibited the growth of at least one Gram-negative test strain. The *Penicillium* sp. extract CHG25-CAG showed 98% to 100% growth inhibitory activity against all four Gram-negative bacterial pathogens (*Acinetobacter baumannii*, *Escherichia coli*, *Klebsiella pneumoniae,* and *Pseudomonas aeruginosa*). Eleven extracts exhibited antifungal activity against *Candida albicans* and/or *Cryptococcus neoformans*. Among them, three extracts, namely, extracts produced by *Streptomyces* sp. (CHG48-GYM) and *Trichoderma* sp. (CHG34-PDA and CKG62-PDA), were active against both fungal pathogens. Anticancer activity was detected in 23 crude extracts. Proliferation of all four cancer cell lines was inhibited by five bacterial (*Micromonospora* sp. CKG20-GYM; *Nocardiopsis prasina* CKG58-GYM; *Streptomyces* sp. CHG40-GYM, CHG48-GYM, CHG64-GYM) as well as ten fungal extracts (*A. luteoalbus* CKG66-CAG; *G. candidum* CKG25-CAG, -PDA; *Penicillium* sp. CHG25-CAG, -PDA, CKG23-CAG; Pleosporaceae CHG49-CAG; and *Trichoderma* sp. CHG34-CAG, -PDA, CKG62-PDA). Five extracts showed somewhat narrow spectrum anticancer activity, for example, *Fusarium* sp. extract CHG38-CAG strongly inhibited the growth of the human melanoma cells (A375, 98%) and colon cancer cells (HCT116, 93%), but was only moderately or poorly active against the lung cancer (A549, 65%) and breast cancer cells (MB231, 40%).

### 2.3. Bioactivity- and Metabolome-Based Selection of Microbial Extracts

In order to prioritize the most promising candidates out of 68 active crude extracts, we applied a two-step selection approach. In the first step, all extracts with (i) high antimicrobial activity (≥80% inhibition) against at least one bacterial and one fungal pathogen, or (ii) high anticancer activity (≥80% inhibition) against at least one cancer cell line, or (iii) both, high antimicrobial and anticancer activity (≥80% inhibition), were selected. This approach allowed us to prioritize 26 extracts, including eight bacterial extracts obtained from *Bacillus* sp. (CKG24-GYM), *Micromonospora* sp. (CKG20-GYM), *N. prasina* (CKG58-GYM), *Pseudomonas anguilliseptica* (CKG38-GYM, -MB), and *Streptomyces* sp. (CHG40-GYM, CHG48-GYM, CHG64-GYM), as well as 18 fungal extracts originating from *A. luteoalbus* (CKG66-CAG), *Fusarium* sp. (CHG38-CAG, -PDA, CKG32-CAG), *G. candidum* (CKG25-CAG, -PDA), *Penicillium* sp. (CHG25-CAG, -PDA, CHG35-CAG, -PDA, CKG23-CAG, -PDA, CKG63-PDA), Pleosporaceae (CHG49-CAG, -PDA), and *Trichoderma* sp. (CHG34-CAG, -PDA, CKG62-PDA).

**Figure 4:**
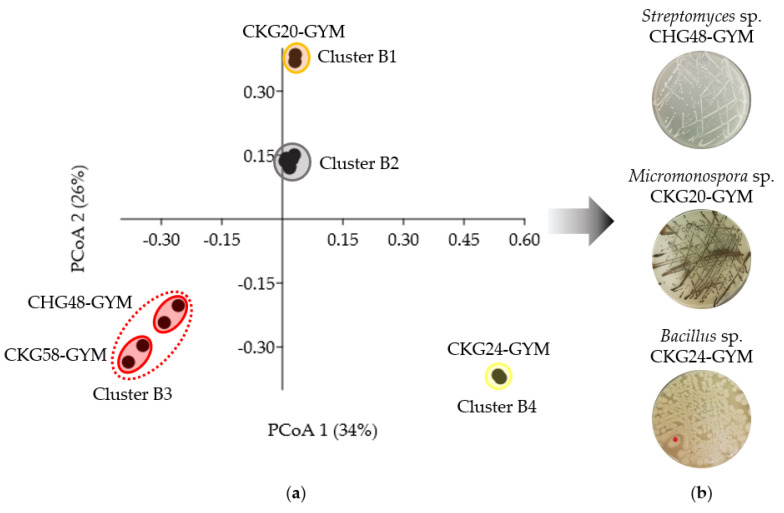
UPLC-MS/MS-based selection of bacterial extracts for in-depth metabolomics. (**a**) PCoA (Principal Coordinates Analysis) plot of eight pre-selected bioactive extracts. Cluster B2 includes extracts produced by *Pseudomonas anguilliseptica* (CKG38-glucose–yeast–malt (GYM) and -MB) and *Streptomyces* sp. (CHG40-GYM and CHG64-GYM). (**b**) Solid cultures of three bacterial extracts prioritized for further chemical investigations.

**Figure 5:**
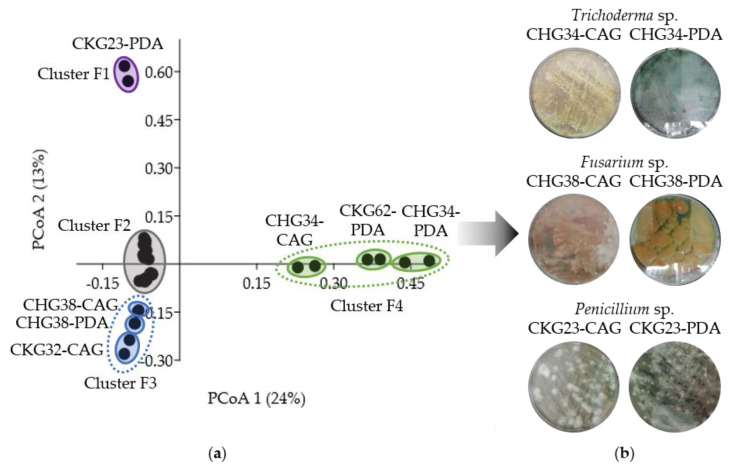
UPLC-MS/MS-based selection of fungal extracts for in-depth metabolomics. (**a**) PCoA plot of 18 pre-selected bioactive extracts. Cluster F2 includes extracts produced by *Acrostalagmus* *luteoalbus* (CKG66-casamino acids–glucose (CAG)), *Galactomyces* *candidum* (CKG25-CAG and -PDA), *Penicillium* sp. (CHG25-CAG and -PDA, CHG35-CAG and -PDA, CKG23-CAG, and CKG63-PDA), and Pleosporaceae sp. (CHG49-CAG and -PDA). (**b**) Solid cultures of six fungal extracts prioritized for further chemical investigations.

In the second step, metabolite profiling by an untargeted UPLC-MS/MS-based approach was applied to the 26 pre-selected extracts in order to detect those with the richest and most diverse chemistry. Pre-processed MS/MS data were converted into peak lists (*m*/*z* value, retention time, intensity) to generate PCoA (Principal Coordinates Analysis) plots reflecting the chemical distinctiveness of the bacterial (Figure 4) and fungal extracts (Figure 5). 

The PCoA plot of the eight pre-selected bacterial extracts clustered into four groups (B1–B4; Figure 4a). Three clusters, i.e., B1, B3, and B4, showed significant differences in their chemical profiles compared to cluster B2 (R = 1, *p* < 0.05; Table S3). The chemically distinct extracts in clusters B1, B3, and B4 had a higher number of detected peaks (80–110) compared to those clustering as B2 (9–66 peaks). *Micromonospora* sp. extract CKG20-GYM and *Bacillus* sp. extract CKG24-GYM clustered separately as B1 and B4, respectively. Accordingly, both were selected for further analysis. Cluster B3 was formed by two actinobacterial extracts with similar chemistry, namely, *N. prasina* extract CKG58-GYM and *Streptomyces* sp. extract CHG48-GYM. The latter extract was selected from cluster B3, since it showed higher bioactivity against *C. neoformans* (100% inhibition at 100 µg/mL; Table S2), and displayed, with 103 peaks, a more distinct metabolome than the other *Streptomyces* extracts CHG40-GYM (61 peaks) and CHG64-GYM (66 peaks) in cluster B2 (Figure S1). Hence, metabolite profiling aided the prioritization of the three bacterial extracts CHG48-GYM (*Streptomyces* sp.), CKG20-GYM (*Micromonospora* sp.), and CKG24-GYM (*Bacillus* sp.) for subsequent metabolomics studies (Figure 4b).

The same process was applied to 18 bioactive fungal extracts resulting in four different clusters in the PCoA plot (clusters F1–F4; Figure 5a). Extracts of three clusters (F1, F3, and F4) had significantly different metabolite profiles compared to extracts from cluster F2 (R = 0.68–1, *p* < 0.01; Table S4). Cluster F1 contained only one extract (*Penicillium* sp. CKG23-PDA), which was selected due to its strikingly different chemical profile. Cluster F3 consisted of three extracts obtained from two *Fusarium* sp. strains CHG38 (CAG and PDA) and CKG32 (PDA). From those, we selected strain CHG38 (CAG and PDA) because of the additional antifungal and anticancer bioactivities observed for the extract CHG38-CAG (96% inhibition against *C. neoformans,* 98% and 93% inhibition against cancer cell lines A375 and HCT116, respectively; Table S2). Similarly, cluster F4 contained in total three extracts from two *Trichoderma* sp. isolates CHG34 (CAG and PDA) and CKG62 (PDA). Revisiting these extracts´ bioactivities led to the selection of *Trichoderma* sp. strain CHG34 (CAG and PDA), as its PDA extract showed in addition strong antibacterial (MRSA, 94%) and antifungal (*C. neoformans,* 92%) activities (Table S2). In addition, we added *Penicillium* sp. CKG23-CAG (cluster F2) to the analysis pipeline, although it did not fulfill the chemical distinctiveness criterion. It shared only few metabolites with the PDA extract from the same *Penicillium* sp. strain (CKG23-PDA), which clustered in F1. Therefore, we aimed to analyze, comparatively, these two chemically different extracts (CKG23-CAG and CKG23-PDA) produced by the same *Penicillium* sp. strain. In total, six fungal extracts were prioritized for further chemical analyses, namely CHG34-CAG and -PDA (*Trichoderma* sp.), CHG38-CAG and -PDA (*Fusarium* sp.), and CKG23-CAG and -PDA (*Penicillium* sp.; Figure 5b). This sums up to a total of nine microbial crude extracts for subsequent in-depth metabolomic analyses. 

### 2.4. IC_50_ Determinations of Prioritized Microbial Extracts

All nine prioritized extracts were subjected to IC_50_ determinations (half maximal inhibitory concentration) against bacterial and fungal human pathogens (Table 1) as well as cancer cell lines (Table 2). The lowest IC_50_ values against the Gram-positive test strains MRSA and *E. faecium* were obtained for *Streptomyces* sp. CHG48-GYM, *Micromonospora* sp. CKG20-GYM, *Bacillus* sp. CKG24-GYM, and *Fusarium* sp. CHG38-CAG (Table 1). Notably, the anti-MRSA potency of *Bacillus* sp. extract CKG24-GYM (IC_50_ value 0.4 µg/mL) was about eight times higher than the positive control chloramphenicol (IC_50_ 3.1 µg/mL). Another very potent bacterium was *Micromonospora* sp. grown in GYM medium (CKG20-GYM), which showed superior activity (IC_50_ 0.1 µg/mL) to the reference antibiotic ampicillin (IC_50_ 0.4 µg/mL) against *E. faecium.* Only *Penicillium* sp. extracts CKG23-CAG and -PDA showed inhibitory activity against the four Gram-negative bacterial test strains *A. baumannii*, *E. coli*, *K. pneumoniae*, and *P. aeruginosa*. Notably, IC_50_ values of the CAG extract were more potent (IC_50_ values between 4.9 and 15.8 µg/mL) than those of the PDA extract (IC_50_ values between 31.4 and 42.6 µg/mL). Concerning antifungal activity, the lowest IC_50_ value against *C. albicans* was exerted by the PDA extract of *Trichoderma* sp. isolate CHG34 (IC_50_ value 3.7 µg/mL). With an IC_50_ value of 13.1 µg/mL, *Streptomyces* sp. CHG48-GYM emerged as the most potent extract towards the yeast-like pathogen *C. neoformans*.

When tested against cancer cell lines, all extracts showed inhibitory activity against at least one cancer cell line (IC_50_ values between 0.02 and 92.3 µg/mL; Table 2). *Streptomyces* sp. extract CHG48-GYM showed the strongest anticancer activity with an IC_50_ value 0.02 µg/mL against the lung carcinoma cell line A549, which was much lower compared to the positive control (IC_50_ 1.3 µg/mL). *Micromonospora* sp. extract CKG20-GYM showed potent in vitro cytotoxicity against all four cancer cell lines (IC_50_ values between 0.8 and 1.6 µg/mL). Among the fungal extracts, *Penicillium* sp. extract CKG23-CAG exerted the strongest antiproliferative effects (IC_50_ values between 2.0 and 8.5 µg/mL).

### 2.5. Feature-Based Molecular Networking and Dereplication of Nine Prioritized Microbial Extracts

We comparatively analyzed the metabolome of the nine prioritized extracts by an integrated dereplication strategy combining FBMN, in silico dereplication tools, and manual approaches. Chemical structures of the putatively annotated metabolites, dereplication tables, and generated FBMNs are shown in the Supplementary Figures S2–S8 and Tables S5–S10. 

**Figure 6:**
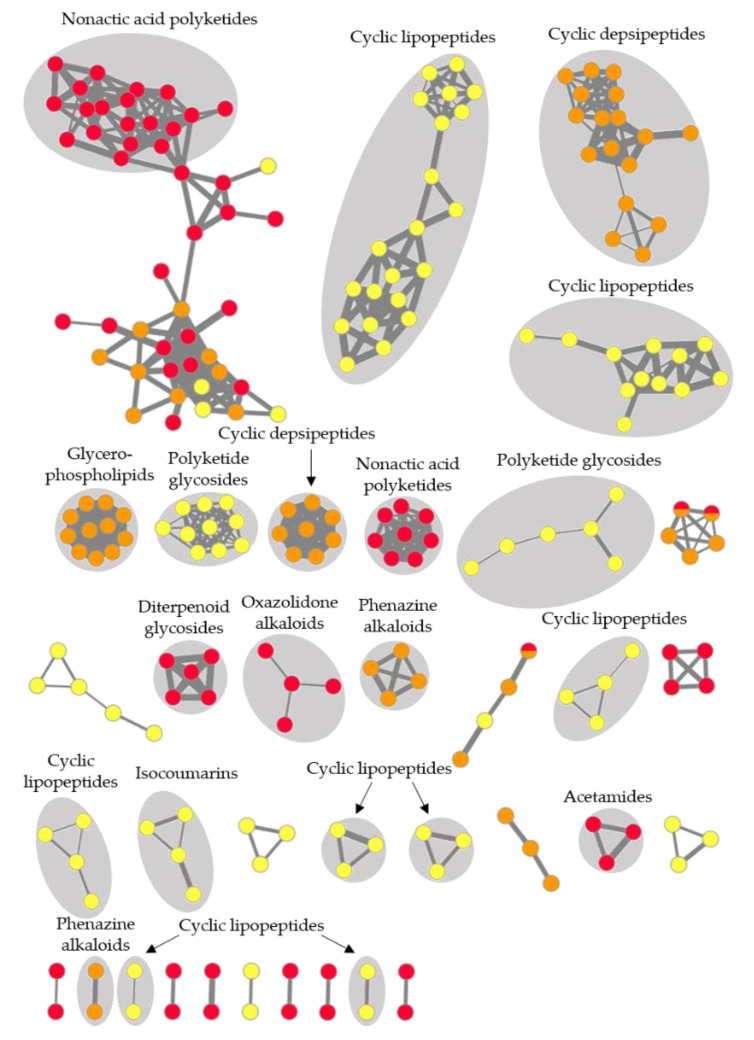
UPLC-MS/MS-based metabolome of three bacterial extracts. The feature-based molecular network (FBMN) displays only clusters containing ≥2 nodes. The width of edges represents the cosine similarity between 2 nodes. Nodes are color-coded by the respective extract: red: *Streptomyces* sp. extract CHG48-GYM; orange: *Micromonospora* sp. extract CKG20-GYM; yellow: *Bacillus* sp. extract CKG24-GYM.

Global metabolome analyses of three selected bacterial extracts, *Streptomyces* sp. CHG48-GYM, *Micromonospora* sp. CKG20-GYM, and *Bacillus* sp. CKG24-GYM, revealed a high metabolite diversity with a total of 220 nodes (ions) organized into 35 molecular clusters (Figure 6). Out of the 35 molecular clusters, 21 were putatively annotated as acetamide derivatives, cyclic peptides (including lipo- and depsipeptides), diterpenoid glycosides, glycerophospholipids, isocoumarin derivatives, nonactic acid polyketides, oxazolidone alkaloids, phenazine alkaloids, and polyketide glycosides. The global FBMN was dominated by several types of cyclic peptides, produced by *Micromonospora* sp. (CKG20-GYM) and *Bacillus* sp. (CKG24G-GYM). The *Bacillus* sp. extract CKG24-GYM exhibited the richest metabolite diversity (89 nodes), followed by *Streptomyces* sp. CHG48-GYM (73 nodes), and *Micromonospora* sp. CKG20-GYM (61 nodes). The chemical diversity of *Bacillus sp.* extract CKG24-GYM was also reflected in the number of exclusive clusters (15 exclusive molecular clusters) compared to CHG48-GYM (11 exclusive molecular clusters) and CKG20-GYM (6 exclusive molecular clusters). Most molecular clusters (91%) in the composite FBMN were exclusive to one bacterial extract. Shared metabolites, only detected in two molecular clusters, remained unannotated. 

In-depth chemical investigations of *Streptomyces* sp. extract CHG48-GYM led to the putative annotation of the alkaloids streptazolin (**2**) and streptenol E (**3**), the diterpenoid glycoside platensimycin B4 (**6**), the linear polyketide alpiniamide A (**7**), and four nonactic acid polyketides (**12**,**14**,**18**,**33**; Figure 6, Figure S3 and Table S5). Nonactic-acid-type polyketides formed the two largest clusters in the molecular network of *Streptomyces* sp., representing protonated ([M + H]^+^) and sodiated adducts ([M + Na]^+^) detected from this chemical family (Figure S3). FBMN-based dereplication led to the putative annotation of, e.g., nonactic acid polyketides. MS/MS library spectra of bonactin (**14**) and homononactyl homononactate (**18**) deposited at the Global Natural Products Social Molecular Networking platform (GNPS, [34]) revealed a precise match with the MS/MS spectra detected for *m*/*z* 401.2540 [M + H]^+^ (**14**) and *m*/*z* 415.2702 [M + H]^+^ (**18**; Figures S9 and S10). This aided manual identification of nonactyl nonactoate (**12**) and nonactin (**33**). Nevertheless, most compounds and clusters could not be linked to any known chemical entity, and, therefore, many compounds (76%) in *Streptomyces* sp. extract CHG48-GYM remained unknown.

The *Micromonospora* sp. isolate CKG20 (GYM medium) showed the lowest chemical diversity of all prioritized bacterial extracts (Figure 6, Figure S4 and Table S6). Putatively identified compounds belonged to phenazine alkaloids (**44**,**48**) and cyclic depsipeptides (**51**–**53**), of which the latter was the dominant chemical family in this extract. Cyclic depsipeptides were represented in three molecular clusters in the FBMN, since MS/MS analysis detected three different adduct types ([M + H]^+^, [M + Na]^+^, [M + K]^+^) that formed their own clusters due to their specific fragmentation patterns. In addition, the GNPS dereplication workflow annotated three compounds to known ubiquitous cell membrane components, i.e., glycerophospholipids (**42**,**45**,**50**). The majority (60%) of the detected metabolites and two molecular clusters did not match with any known compound.

Putatively identified compounds of *Bacillus* sp. extract CKG24-GYM were classified into three different chemical families, namely, isocoumarin derivatives (**55**,**59**,**60**), polyketide glycosides (**64**), and various cyclic lipopeptides (**61**–**63**,**65**,**66**,**68**–**72**,**74**,**76**,**77**,**79**,**81**,**82**,**84**–**87**; Figure 6, Figure S5 and Table S7). In the FBMN, the dominance of cyclic lipopeptides was reflected by eight molecular clusters putatively annotated to this NP family. Putatively annotated cyclic lipopeptides can be further classified into bacillomycins (**61**–**63**; *m*/*z* 1071.5811–1099.6122 [M + H]^+^), plipistatins (**65**,**66**,**68**–**72**; *m*/*z* 731.4171–753.4296 [M + 2H]^2+^), and surfactins (**74**,**76**,**77**,**79**,**81**,**82**,**84**–**87**; *m*/*z* 994.6426–1064.7209 [M + H]^+^; Figure S5, Table S7). The putative assignment to three different subfamilies (bacillomycins, plipistatins, surfactins) and the detection of different adduct types (e.g., surfactins: [M + H]^+^ and [M + Na]^+^ adducts) explain the formation of several distinct lipopeptide clusters. The highest annotation rate in this study (71%) was achieved for this extract, i.e., only 10 compounds (**54**,**56**–**58**,**67**,**73**,**75**,**78**,**80**,**83**) remained unannotated.

**Figure 7:**
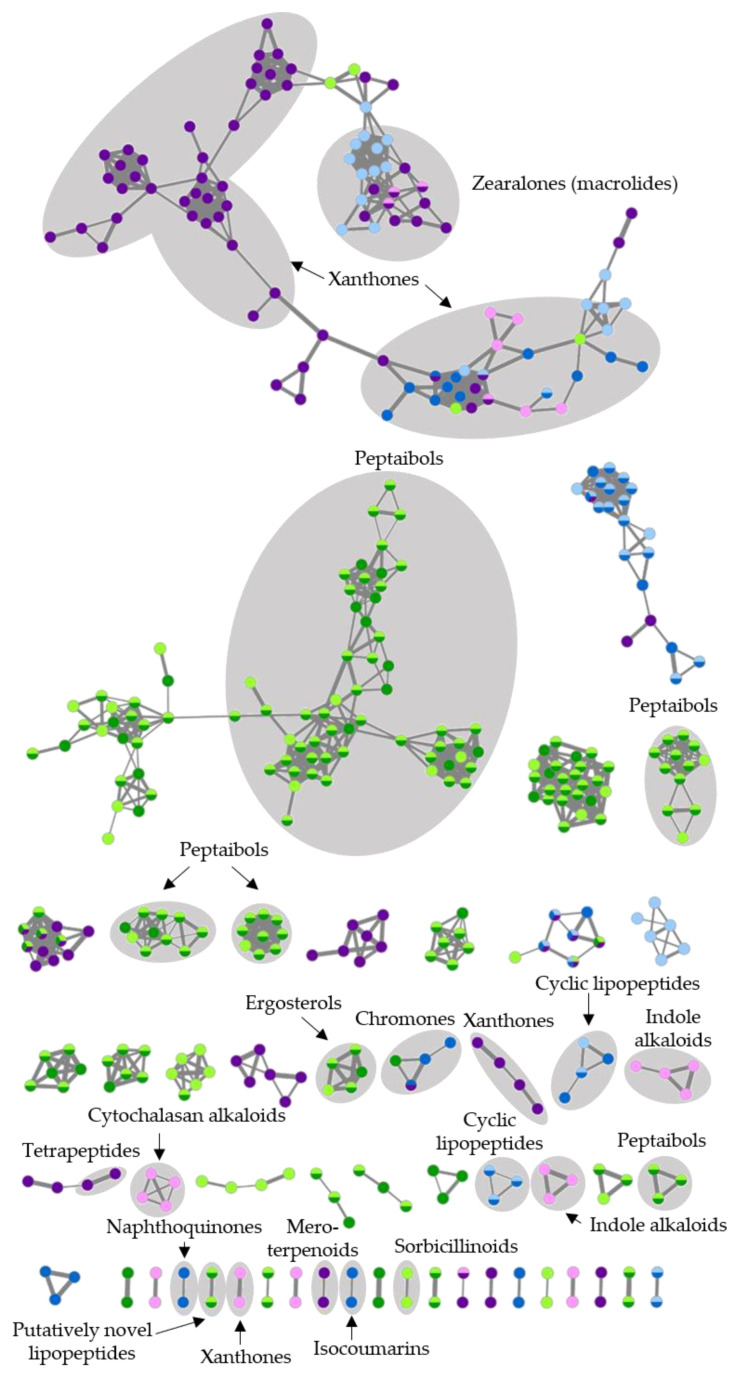
Global UPLC-MS/MS-based metabolome of six fungal extracts. The FBMN displays only molecular clusters containing ≥2 nodes. The width of edges represents the cosine similarity between 2 nodes. Nodes are color-coded by the respective extract (CAG: light color; PDA: strong color): green: *Trichoderma* sp. strain CHG34; blue: *Fusarium* sp. strain CHG38; purple: *Penicillium* sp. strain CKG23.

FBMN-based analysis also proved the high metabolite diversity (411 ions in total, 52 distinct molecular clusters) of the six selected fungal extracts (Figure 7). Putatively annotated clusters included alkaloids (indole and cytochalasan alkaloids), peptides (e.g., peptaibols), polyketides (e.g., macrolides), steroids (ergosterols), and terpenoids (mero- and sesquiterpenoids). The largest molecular cluster, putatively annotated by GNPS and in silico dereplication workflows, is xanthone and zearalenone type polyketides. It contained metabolites produced by all three fungal strains. With the exception of this shared polyketide cluster, the chemical diversity of the selected strains differed. As an example, *Trichoderma* sp. showed 171–173 nodes and 24 exclusive clusters in the global network and was the most chemically diverse. *Fusarium* sp. and *Penicillium* sp. produced only eight and fifteen exclusive clusters, respectively.

**Figure 8:**
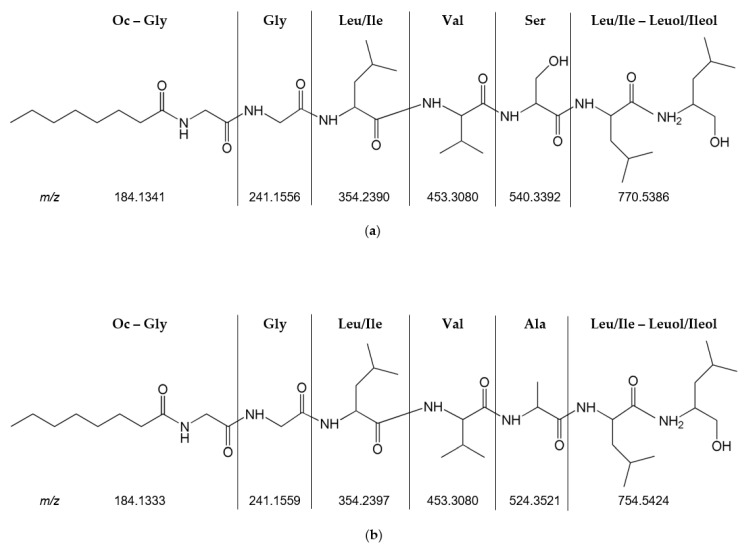
Two putatively novel seven-residue lipopeptides detected in *Trichoderma* sp. extracts CHG34-CAG and CHG34-PDA. Structures were putatively predicted based on the experimentally determined MS/MS fragments for (**a**) compound **103** (*m*/*z* 770.5386 [M + H]^+^) and (**b**) compound **105** (*m*/*z* 754.5423 [M + H]^+^). Ala: alanine; Gly: glycine; Leu/Ile: (iso)leucine (leucine is displayed); Leuol/Ileol: (iso)leucinol (leucinol is displayed); Oc: octanoyl; Ser: serine; Val: valine.

*Trichoderma* sp. isolate CHG34 produced ergosterols, sorbicillinoid-type polyketides, and peptaibols (Figure 7, Figure S6 and Table S8), of which the latter dominated the metabolome. Various peptaibols such as trichokindins (**125**,**143**,**144**,**147**,**150**,**151**,**154**,**155**,**157**) and neoatroviridins (**149**,**156**,**160**) were putatively annotated. As depicted in the FBMN (Figure S6), peptaibols are often detected as doubly charged (sodiated) ions ([M + 2H]^2+^ and [M + 2Na]^2+^) [35,36]. Accordingly, several distinct peptaibol clusters such as trichokindins and neoatroviridins were annotated. Despite our integrated dereplication efforts, most compounds (73%) in the *Trichoderma* extracts remained unannotated (Table S8). The amino acid sequence of two unannotated compounds, *m*/*z* 770.5386 [M + H]^+^ (**103**) and *m*/*z* 754.5424 [M + H]^+^ (**105**) (highlighted as “putatively novel lipopeptides” in the global MN; Figure 7 and Figure S6 and Table S8), were putatively predicted based on characteristic MS/MS fragments (Figure 8 and Figure S11). Tandem mass spectrometry (MS/MS) is an essential tool in peptide chemistry since mass differences of produced fragment ions allow the determination of the amino acid sequence of peptides [37]. Analysis of the MS/MS spectra of **103** and **105** revealed that both compounds contained seven amino acid residues. The first observed fragment of **103** (*m*/*z* value of 184.1341) reflects the fatty acyl moiety Oc (octanoyl) connected to Gly (C_10_H_18_NO_2_) at the N-terminus of the putative peptide. The loss of *m*/*z* 230.1994 (C_12_H_26_N_2_O_2_) positioned Leu/Ile-Leuol/Ileol at the C-terminus of **103**. The full sequence of **103** (*m*/*z* 770.5386 [M + H]^+^) determined by MS/MS fragmentation was proposed as N-Oc-Gly-Gly-Leu/Ile-Val-Ser-Leu/Ile-Leuol/Ileol. The second putatively novel linear peptide (**105**, *m*/*z* 754.5424 [M + H]^+^) had a similar amino acid sequence as **103** but with Ser replaced by Ala. Accordingly, these two molecular ions may be novel linear seven-residue lipopeptides produced by *Trichoderma* sp. strain CHG34. The CAG and PDA extract of *Trichoderma* sp. CHG34 showed a high overlap of their chemical space (82%) with 61 shared metabolites (Table S8). 

In *Fusarium* sp. extracts CHG38-CAG and -PDA, the cyclic lipopeptide fusaristatin A (**182**) and chromone (**164**,**165**), isocoumarin (**166**), naphthoquinone (**162**,**163**,**168**), xanthone (**171**,**178**), and zearalenone (**169**,**176**,**179**) polyketides were annotated (Figure 7, Figure S7 and Table S9). According to the cluster analysis, xanthone and zearalenone polyketides dominated the FBMN by forming the two largest clusters. Zearalenone (**176**) was predicted by the GNPS-based MS/MS spectral match and guided us to putatively annotate 2‣-hydroxyzearalanol (**169**) and zearalanone (**179**) in the same cluster. The majority of compounds (62%) remained unidentified. Notably, cultivation of *Fusarium* sp. strain CHG38 triggered the production of several metabolites that were exclusive to either CAG (10 compounds) or PDA (16 compounds) medium.

The metabolome of *Penicillium* sp. strain CKG23 contained eight different chemical families, such as cytochalasans (**209**,**212**), indole alkaloids (**206**,**219**), meroterpenoids (**222**), sesquiterpenoids (**213**), and various types of polyketides including zearalenone derivatives (**197**,**199**,**205**,**208**,**224**; Figure 7 and Figure S8, Table S10). The largest cluster in the FBMN belonged to the polyketide family xanthones, which were also detected in *Fusarium* and *Trichoderma* sp. extracts in the global FBMN. Notably, the CAG and PDA extracts of *Penicillium* sp. strain CKG23 shared only seven molecular ions (Figure S12). Moreover, CKG23-PDA showed a strikingly higher chemical diversity with 185 detected peaks compared to CKG23-CAG (51 peaks). For example, most putatively identified compounds in the zearalenone cluster were only detected in the PDA extract of *Penicillium* sp. (**197**,**199**,**205**,**224**), while no compound was exclusive to extract CKG23-CAG.

The untargeted metabolomics approach employed here revealed the huge chemical inventory of nine microbial extracts (Table 3). The metabolomes showed large variations between the different microbial taxa, but also the applied cultivation media impacted the chemical diversity. Compared to low annotation rates of <2% normally achieved in untargeted metabolomics studies [38], the integrated dereplication effort applied herein significantly improved the putative annotation rates ranging from 24% (*Streptomyces* sp. extract CHG48-GYM) to 71% (*Bacillus* sp. extract CKG24G). Nevertheless, many compounds and molecular clusters did not match any known compounds, suggesting that they could represent putatively new compounds.

## 3. Discussion

In the present study, the unexplored gut microbiota of the tunicate *C. intestinalis* was investigated for its potential to deliver novel MNPs with pharmaceutical potential. The obtained strain collection represents with 101 gut-associated bacteria and fungi (Figure 1a, Table S1) the most comprehensive strain collection from the tunicate’s gut available to date. A diverse microbial community (i.e., 33 different genera) was obtained from six different isolation media, which showed different suitability for growth of a diverse array of microorganisms (Figure 1b). For instance, glucose is a common carbon source for fungi [39], and, accordingly, most fungi were obtained from the glucose-containing media, WSP and PDA. In addition, media containing several carbon sources and other complex compounds often yield the highest microbial diversity [18], and this is in line with our finding that most isolates were obtained from the complex WSP medium (glucose, malt extract, peptone, and yeast extract). This indicates that the selection of isolation media has a huge impact on the isolated microbiota due to specific nutrient requirements of different microorganisms [18,19]. Furthermore, other cultivation conditions such as temperature (22 °C) also significantly influence the cultivable fraction of bacteria and fungi [18,19]. 

Although highly abundant bacterial genera such *Shewanella* and *Vibrio* and fungal genera such as *Penicillium* and *Trichoderma* (Figure 2) were previously isolated from the gut of *Ciona* spp. [23,26,27,28], most microbial genera were isolated for the first time from the gut of *C. intestinalis*. The high abundance of Gammaproteobacteria is in accordance with a previous culture-independent study performed on the gut microbiome of *Ciona* spp. [29]. We have recently described the cultivable microbiota of the tunic of *C. intestinalis* [31] that differed strikingly from the gut-associated microbial community isolated herein. Both tissues shared only few microbial genera (e.g., bacteria: *Bacillus* and *Vibrio*; fungi: *Fusarium* and *Penicillium*), which is in accordance with culture-independent microbiome studies on the gut and tunic of *Ciona* spp., indicating tissue-specific microbial communities [29,30]. Moreover, the diversity of culture-dependent fungi was higher in the gut (gut: 40 isolates assigned to 20 genera; tunic: 22 isolates, 15 genera), while bacteria were more prominent in the tunic (89 isolates assigned 37 genera; gut: 61 isolates, 13 genera) [31]. In line with our previous study on the tunic-associated microbiota [31], the gut-associated microbiota, especially the bacterial community, was more diverse in Kiel than in Helgoland samples (Figure 1 and Figure 2). This may be attributed to different salinity levels (Kiel: brackish, Helgoland: oceanic salinity) and the comparably higher anthropogenic input, i.e., more eutrophic conditions, at the sampling site in Kiel Fjord [31]. Moreover, samples were obtained from different depths (Helgoland: <1 m depth; Kiel: approx. 3 m depth) and different artificial surfaces (Helgoland: pontoon; Kiel: mussel-cultivation basket), which both may have influenced the obtained microbial diversity. Other parameters not determined in this study, e.g., diet, water temperature, and the age and genetic background of the sampled specimens, may be additional factors shaping the diversity of the cultivable microbiota [18,40,41].

Gut-derived microbial extracts (*n* = 103) were screened against a panel of cancer cell lines and microbial pathogens, since ascidians and their associated microorganisms are well-known producers of MNPs with antimicrobial and anticancer properties [11,13,42]. The antimicrobial assays included the so-called ESKAPE panel, drug-resistant bacterial pathogens that were categorized by the WHO as priority level 1 and 2 for the discovery of new antimicrobial agents [43]. Most extracts (*n* = 65) exhibited activity against MRSA and/or *E. faecium*, but also anticancer (*n* = 23) and antifungal (*n* = 11) activities were observed (Figure 3, Table S2), exceeding bioactivity levels previously reported for cultivable bacteria associated with solitary ascidians [44,45]. The high rate of bioactivity observed in this study is in line with the excellent biodiscovery potential reported for tunicates and their microbial associates [11,13,42]. As outlined before, an intact gut microbiota has crucial functions for the health and performance of its host [1,3]. Possible functions related to chemical defense and nutrition were already proposed for cultivable bacteria obtained from the intestine of a solitary ascidian and a sea urchin [46,47]. The high proportion of microorganisms with, e.g., antibacterial properties (64%) indicates their potential involvement in the tunicate’s chemical defense. However, it was beyond the scope of this study to detect specific functions fulfilled by specific cultivable gut-associated microorganisms in the host–microbiota interplay.

Bioactive crude extracts (*n* = 68) were subjected to a two-step selection procedure considering the bioactivity profile and chemical diversity of the extracts to prioritize the most promising extracts for in-depth metabolome mining. This strategy aided prioritization of nine microbial extracts affiliated to the bacterial genera *Bacillus*, *Micromonospora*, and *Streptomyces* as well as the fungal genera *Fusarium*, *Penicillium*, and *Trichoderma* (Figure 4 and Figure 5). These microbial genera are known as the most talented producers of MNPs highlighting the strength of the applied prioritization pipeline (e.g., [9,48,49]). 

The integrated dereplication approach combining automated and manual dereplication tools allowed the putative identification of 94 metabolites (Tables S5–S10). They belonged to various NP classes such as alkaloids, lipids, peptides, polyketides, steroids, and terpenoids, revealing a huge metabolic capacity of the prioritized microbial strains. Dereplication was substantially supported by the recently released FBMN workflow [32], which, in combination with other tools, led to annotation rates of up to 71%. Increasing annotation rates in untargeted metabolomic experiments is crucial to overcome time-consuming re-isolation of known compounds, which severely hampers biodiscovery efforts [33].

Untargeted metabolomics studies on fungi (e.g., *Fusarium* and *Penicillium* spp. [31,50,51]) and bacteria (e.g., *Streptomyces* and *Salinispora* spp. [52]) cultured on the same medium already revealed huge chemical variations at both species and strain level and are therefore used as chemotaxonomic species discrimination markers ([51] and references therein). Hence, we expected to find distinct chemical profiles of strains from the same genus, e.g., *Penicillium* or *Streptomyces* (Figure 4, Figure 5 and Figure S1, Tables S3–S4). Beyond this, variations in metabolite diversity in different media is also a well-known phenomenon in the OSMAC (one strain–many compounds) approach [53]. In line with this, *Fusarium* sp. strain CHG38 and *Penicillium* sp. strain CKG23 showed differential bioactivities and metabolomes when cultured on two different media (Figure 7, Figure S7–S8 and S12, Table 1, Table 2 and Tables S9–S10). Furthermore, the fungal cultivation media CAG and PDA appeared to trigger the production of various (bioactive) metabolites, e.g., the metabolomes of *Fusarium* sp. CHG38 and *Penicillium* sp. CKG23 were richer when cultured on PDA medium, while cultivation of *Trichoderma* sp. on CAG yielded more compounds (Figure 7, Tables S8–S10). Both media contain the simple sugar glucose, an ideal carbon source for fungal growth [39], plus an additional mixed carbon source, which seemingly meets requirements for fungal growth and production of secondary metabolites. In contrast, GYM and MB media used for bacterial strains were not equally suitable as only one bacterial extract obtained from cultivation on MB medium met the bioactivity selection criterion (*P. anguilliseptica* extract CKG38-MB), while seven GYM extracts exerted strong antimicrobial and/or anticancer activities (Table S2). While MB medium mimics the major mineral composition of seawater, GYM medium lacks these minerals, but contains glucose and malt extract, which are easily accessible carbon sources. However, eleven bacterial strains, including all *Vibrio* sp. isolates, failed to grow on GYM medium. This may be attributed to the lack of sodium chloride (NaCl) in this medium, since most *Vibrio* spp. require NaCl for growth [54]. Apart from the media composition, other parameters such as the temperature, solid regime (in contrast to liquid cultures) or aeration may have influenced the observed chemical composition [53,55]. Moreover, artificial laboratory conditions often lack important environmental cues such as multispecies interactions, which can lead to, e.g., silencing of important biosynthetic gene clusters [55]. Therefore, the obtained metabolomic compositions may not necessarily reflect the true metabolite repertoire of the organism [55].

The observed anti-MRSA and *E. faecium* activity of *Streptomyces* sp. extract CHG48 (Table 1) might be explained by the diterpenoid glycoside platensimycin B (**6**) and two nonactic acid polyketides (**18**,**33**), all with reported antibacterial activities [56,57,58,59]. The putatively annotated polyketide homononactyl homononactate (**18**) shows weak activity against colon cancer cell line HCT116 [60], but not against lung cancer cell line A549 [56]. Hence, this compound cannot explain the detected selective anticancer activities against cell lines A549 and A375 (Table 2). Moreover, none of the dereplicated metabolites can explain the detected antifungal activities of *Streptomyces* sp. extract CHG48-GYM against *C. albicans* and *C. neoformans* (Table 1), and the annotation rate was the lowest detected in this study (24%; Table S5). We therefore consider *Streptomyces* sp. isolate CHG48 as a promising candidate strain for isolation of its chemical constituents.

The antibacterial and anticancer activities (Table 1 and Table 2) of *Micromonospora* sp. (CKG20-GYM) may be attributed to the putatively annotated cyclic depsipeptides rakicidins (**51**–**53**) **[61,62,63]** and the phenazine alkaloid diazepinomicin (**44**) [49,64] (Figure 6 and Figure S4, Table S6), for which these activities are known. About 60% of the detected compounds remained unannotated, including a putatively novel rakicidin derivative (**49**), which is worth investigating in future studies. 

The *Bacillus* sp. extract CKG24-GYM inhibited the growth of microbial pathogens and showed antiproliferative activity against cancer cell lines A375, A549 and HCT116 (Table 1 and Table 2). Anti-MRSA activity has been described for the putatively identified isocoumarin derivative amicoumacin-A (**55**) [65] and the polyketide glycoside aurantinin B (**64**) [66]. Inhibitory activities against colon cancer cell line HCT116 are reported for some surfactin-like lipopeptides (**79**,**82**,**84**,**86**) [67]. Bacillomycins (**61**–**63**) [68] and some cyclic lipopeptides (**65**,**66**,**68**–**72**) reportedly show antifungal properties [69] possibly relating to the observed fungicidal activity of CKG24-GYM. In combination with the high annotation rate (71%), these results render *Bacillus* sp. extract CKG24-GYM not favorable for future studies.

Among the fungi, *Trichoderma* spp. are the major source of peptaibols [70,71] and, accordingly, extracts of *Trichoderma* sp. strain CHG34 were dominated by this NP class (Figure 7 and Figure S6, Table S8). Moderate anticancer activities were observed in both *Trichoderma* sp. extracts (CHG34-CAG, -PDA; Table 2), which may originate from the putatively identified peptaibols neoatroviridin B–D (**149**,**156**,**160**) [72]. Antibacterial activity was only detected in the PDA extract of *Trichoderma* sp. strain CHG34 (Table 1), but none of the compounds exclusive to this extract (**90**,**92**,**111**) could be putatively identified as annotation rates remained low for *Trichoderma* sp. strain CHG34 (27%). Two new lipopeptide structures (**103**,**105**) were proposed based on the predicted amino acid sequences from the product ions. Future efforts will encompass isolation and confirmation of the putative structures and also exploration of this strain for other novel MNPs for the treatment of infectious diseases.

As outlined above, *Fusarium* sp. extracts showed different bioactivities and metabolomes (Figure 7, Figure S7, Table 1, Table 2 and Table S9). The antimicrobial and anticancer properties of *Fusarium* sp. extract CHG38-PDA may originate from the putatively annotated xanthone derivative griseoxanthone C (**178**) [73,74] and the naphthoquinone norjavanicin (**163**) [75]. In addition, the shared metabolite fusaristatin A (**182**) reportedly inhibits the proliferation of lung cancer cells [76]. *Fusarium* spp. are also prominent producers of mycotoxins, including the macrolide zearalenone (**176**) detected in this study [77]. Zearalenone (**176**) shows antifungal but no antibacterial activities [78]. Hence, antibacterial activities of *Fusarium* sp. extract CHG38-CAG remain unresolved, since none of its putatively annotated compounds have been shown to possess antibacterial activities. Accordingly, extract CHG38-CAG is highlighted for future chemical investigations. 

Detailed LC-MS/MS analyses of the *Penicillium* sp. isolate CKG23 revealed distinct chemical profiles of its CAG and PDA extracts (Figure 5a, Figure 7, Figures S8 and S12, Table S10). Anticancer activities of CKG23-CAG may be attributed to the putatively identified indole alkaloid communesin B (**219**) and the cytochalasan chaetoglobosin A (**212**) that were shown to inhibit the proliferation of cancer cell lines A549 (**212**,**219**) and HCT116 (**219**) [79,80]. In addition, the meroterpenoid andrastin A (**222**) putatively annotated in CKG23-CAG is a farnesyltransferase inhibitor that prevents correct functioning of, e.g., RAS proteins (common oncogenes), rendering it a promising anticancer lead compound [81]. Antiproliferative activity of CKG23-PDA and observed antibacterial activities (including Gram-negative pathogens) of both *Penicillium* sp. extracts could not be linked to any of the dereplicated compounds. Moreover, extract CKG23-PDA had the most distinct chemistry of all *Penicillium* sp. extracts (Figure 5a), indicating that the PDA extract of *Penicillium* sp. CKG23 is worth pursuing in future studies.

In this study, we showed that the cultivable fraction of the gut-associated microbiota of *C. intestinalis* is diverse and specific. The majority of the yet unexplored gut-associated cultivable microorganisms showed antimicrobial and/or anticancer activities, suggesting a significant potential for discovery of new drug leads. Computationally assisted metabolome mining of nine prioritized bioactive crude extracts led to the putative identification of 94 metabolites assigned to 24 different chemical families. Nevertheless, most compounds and molecular clusters remained unannotated and may therefore be novel MNPs. Several extracts containing putatively novel and bioactive metabolites were highlighted, in particular *Streptomyces* extract CHG48-CAG, *Trichoderma* sp. extracts CHG34-CAG and CHG34-PDA, *Fusarium* sp. extract CHG38-CAG, and *Penicillium* sp. extract CKG23-PDA. This emphasizes the benefit of working with marine microorganisms obtained from previously unexplored sources and is in line with the strongly increasing contribution of microorganisms in MNP discovery [9]. It is widely accepted that MNPs have higher success rates in drug discovery compared to their terrestrial counterparts [82]. Being evolved and prevalidated by natural pressures and adaptation processes for millions of years, NPs represent “privileged scaffolds in drug discovery” [83], whose bioactivity and structural diversity surpass any synthetic compound prepared in the laboratory [84]. To conclude, the obtained cultivable gut-associated microbial community of *C. intestinalis* delivered several promising microorganisms that seem to harbor an unexplored chemical space. Putatively novel compounds and their bioactivities need to be verified in future isolation and structure elucidation studies.

## 4. Materials and Methods 

### 4.1. Sampling and Isolation of Microorganisms

*Ciona intestinalis* was sampled in September 2017 at two locations, the German North Sea island Helgoland ( Germany, 54.177102, 7.893053) and Kiel Fjord (Kiel, Germany, Baltic Sea, 54.382062, 10.162059). Samples were collected in local harbors from a pontoon (Helgoland, <1 m depth) or from a mussel cultivation basket (Kiel Fjord, approximately 3 m depth). Dissection and inoculation of microbiological samples were conducted at the same day. 

Six solid media (1.8% agar) were used to isolate a diverse bacterial and fungal community from the ascidian’s gut: two *C. intestinalis* media adjusted to Baltic (CB) and North Sea salinity (CN; [31]), MB (3.74% Marine Broth 2216), potato dextrose agar (PDA [85]), TSB (0.3% trypticase soy broth, 1% sodium chloride) and modified Wickerham medium (WSP [86]). Agar (bacteriology grade) and sodium chloride were purchased from AppliChem (Darmstadt, Germany), malt extract, Marine Broth, and trypticase soy broth from Becton Dickinson (Sparks, MD, USA), glucose, peptone (from soymeal), and granulated yeast extract from Merck (Darmstadt, Germany), and potato infusion powder was ordered at Sigma Aldrich (Steinheim, Germany). 

The gut (*n* = 2 biological replicates per sampling site) was carefully dissected and placed into a sterile 1.5 mL reaction tube. The dissected gut was diluted 1:1 with sterile artificial seawater (1.8% (K) or 3% (H) Instant ocean, Blacksburg, VA, USA) and gently homogenized with a sterile pestle. Agar media were inoculated with 100 µL aliquots and their 1:10 and 1:100 dilutions. Petri dishes were checked for growth of fungi and bacteria after incubation for one and three weeks in the dark at 22 °C. Microbial colonies showing distinct macroscopic phenotypes were transferred to fresh agar plates until pure cultures were obtained. Microbial strains were cryo-conserved at −80 °C using the ready-to-use Microbank^TM^ system (Richmond Hill, ON, Pro Lab Diagnostics, Canada).

### 4.2. DNA Extraction and Identification of Microbial Isolates

DNA was extracted by applying a freeze and thaw protocol (bacteria) or by mechanical lysis (fungi) as described elsewhere [31,87]. If subsequent PCR amplification of the target fragment failed, DNA extraction was repeated with the DNeasy Plant Mini Kit (Qiagen, Hilden, Germany) according to the manufacturer’s instructions. Some modifications of the protocol were applied as previously described [31].

Molecular identification was performed following established protocols for amplification of the 16S rRNA gene (bacteria) or the ITS1-5.8S-ITS2 region (fungi) [87]. The 18S and 28S rRNA gene were additionally amplified for fungal strains with ambiguous identification (for protocols see [88,89]). PCR conditions for amplifying the large ribosomal subunit of the rRNA were modified as previously described [31]. Sanger sequencing [90] of successfully amplified DNA fragments was conducted at LGC Genomics GmbH (Berlin, Germany). FASTA files of quality checked and trimmed DNA sequences were searched against the NCBI (National Center for Biotechnology Information) nucleotide database using BLAST (Basic Local Alignment Search Tool [91]). One bacterial isolate (CKG60) could only be identified at family level. Application of Naive Bayesian rRNA Classifier v2.11 of the Ribosomal Database Project using the 16S rRNA training set at a 95% confidence threshold [92] resulted in identification of this isolate to genus level. Bacterial and fungal DNA sequences were deposited in GenBank under the accession numbers MW065489-549 (gut-associated bacteria), MW064137-74 (gut-associated fungi, ITS), and MW064175-6 (gut-associated fungi, 18S; Table S1).

### 4.3. Cultivation and Extraction of Gut-Associated Microbial Strains

Out of 101 gut-derived microbial isolates, 29 were excluded from cultivation due to laboratory safety concerns based on the Technical Rules for Biological Agents (TRBA 460, TRBA 466). Fifteen additional strains were excluded, since they were affiliated to the same species as another strain isolated from the same sampling site, which led to the final selection of 27 bacterial and 30 fungal strains for cultivation (*n* = 57; Table S2). Microbial isolates were cultured in duplicate (i.e., two biological replicates) on two different media. Bacterial isolates were cultured on the solid agar media glucose–yeast–malt (GYM [93]) and MB. Solid casamino acids–glucose (CAG [94]) and PDA media were used for growing fungal strains. Ingredients not listed in Section 4.1. were purchased from Carl Roth (Karlsruhe, Germany). CAG, GYM and PDA were chosen due to their excellent potential to trigger production of novel and bioactive compounds (see [31] and references therein). MB medium was chosen to ensure sufficient growth of all bacterial isolates. For cultivation of precultures, solid media were inoculated with cryo-conservation beads and incubated in the dark at 22 °C until the agar was completely overgrown. Main cultures were inoculated by transferring microbial colonies or an overgrown piece of agar with an inoculation loop to the respective agar medium. Fungi were inoculated on five agar plates and bacteria on ten plates in parallel. As outlined above, each strain was cultivated on two respective media in duplicate yielding 20 (fungi) or 40 (bacteria) petri dishes per strain. Microbial cultures were grown in the dark for seven (bacteria) or 21 (fungi) days at 22 °C. Eleven bacterial strains did not grow on GYM medium, hence were only cultivated on MB medium.

For solvent extractions, the agar was cut with a flat spatula and mixed with 200 mL (fungi) or 400 mL (bacteria) ethyl acetate (EtOAc; VWR International, Leuven, Belgium) in a glass bottle. The mixture was homogenized with a T25 basic Ultra Turrax (IKA-Werke, Staufen, Germany; 30 s at 13,000 rpm), which was followed by maceration overnight (120 rpm, 22 °C). The solvent was decanted and washed with an equal volume of ultra-purified water to remove salts (Arium Lab water systems, Sartorius, Goettingen, Germany) in a liquid–liquid partitioning experiment. The EtOAc phase was transferred to a round bottom flask. The extraction process was repeated in the same manner, with the only exception that the extraction process was reduced to 15 min in an ultrasonic bath. Combined EtOAc extracts were evaporated to dryness and re-solubilized in 4 mL methanol (MeOH; ULC-MS grade, Biosolve Chimie, Dieuze, France). Extracts were filtered through a 0.2 μm PTFE (polytetrafluoroethylene) filter (VWR International, Darmstadt, Germany), dried again in pre-weighed vials to determine their extract weight, and stored at −20 °C. Media blanks were prepared as controls by using the same protocol.

### 4.4. Biological Assays

Dried crude extracts were dissolved in dimethyl sulfoxide (Carl Roth) at a concentration of 20 mg/mL. Microbial pathogens included the ESKAPE panel (*Enterococcus faecium*, Efm, DSM 20477; methicillin-resistant *Staphylococcus aureus*, MRSA, DSM 18827; *Klebsiella pneumoniae*, Kp, DSM 30104; *Acinetobacter baumannii*, Ab, DSM 30007; *Pseudomonas aeruginosa*, Psa, DSM 1128; *Escherichia coli*, Ec, DSM 1576), as well as the pathogenic fungi *Candida albicans* (Ca, DSM 1386) and *Cryptococcus neoformans* (Ca, DSM 6973). The following four cancer cell lines were selected for anticancer assays: A375 (malignant melanoma cell line), A549 (lung carcinoma cell line), HCT116 (colon cancer cell line), and MDA-MB231 (human breast cancer line). Test organisms and cell lines were purchased from Leibniz Institute DSMZ-German Collection of Microorganisms and Cell Cultures (Braunschweig, Germany) and CLS Cell Lines Service (Eppelheim, Germany). Tests were performed in 96-well plates at a final concentration of 100 µg/mL as previously described [95,96]. For each extract, its two biological replicates were tested in duplicate each (i.e., two technical replicates). The antibiotics amphotericin (Cn), ampicillin (Efm), chloramphenicol (MRSA, Ec, Kp), doxycycline (Ab), nystatin (Ca), polymyxin B (Psa), and the cytostatic agent doxorubicin (cancer cell lines) served as positive controls. Prioritized extracts were additionally subjected to IC_50_ determinations (also two biological and two technical replicates) by applying a previously published protocol [95].

### 4.5. Metabolomic Analyses

#### 4.5.1. UPLC-QToF-MS/MS Measurements

All solvents used for UPLC-based metabolomic analyses were ordered at Biosolve Chimie or LGC Standards (Wesel, Germany) in ULC-MS grade. Crude extracts (two biological replicates each) re-dissolved in MeOH (final concentration of 1.0 mg/mL) were measured on an Acquity UPLC I-Class System connected to a Xevo G2-XS QToF Mass Spectrometer (Waters, Milford, MA, USA). Crude extracts were injected (0.3 µL) and separated on an Acquity UPLC HSS T3 column (High Strength Silica C18, 1.8 μm, 2.1 × 100 mm, Waters) operating at 40 °C. The binary mobile phase consisted of ultra-purified water (A) and acetonitrile (B), both spiked with 0.1% formic acid. The elution gradient pumped at a flow rate of 0.6 mL/min was as follows (% of A given): initial, 99%; 11.5 min, 1%; 14.5 min, 1%; 14.5–16 min 99%. LC-MS chromatograms and MS/MS fragmentation spectra were recorded as previously described [31]. The same settings were applied to analyze MeOH (solvent control) and media blanks.

#### 4.5.2. Pre-Processing of UPLC-MS/MS Data and Statistics

The ProteoWizard tool msconvert 3.0.20010 was used to transform acquired spectra to mzXML format [97]. Quality filtering and removal of media and contaminant peaks were carried out in MZmine 2.53 [98]. Briefly, mass lists were compiled for compounds with a retention time (R_t_) between 1 and 12 min with an intensity above 30,000 (MS) or 50 (MS/MS). Chromatograms were built at a minimum peak height of 60,000 and an *m*/*z* tolerance of 0.005 Da or 15 ppm. Deconvolution of chromatograms was performed with the baseline cut-off algorithm using the same noise level and peak height as above. Isotope grouping and alignment of peaks was performed with an *m*/z tolerance of 0.001 Da or 10 ppm and an R_t_ tolerance of 0.5 min. The alignment of peaks was conducted with the join aligner method by using an *m*/*z* to R_t_ ratio of 75:25. Finally, peak lists were filtered with an *m*/*z* range of 150–1200 Da. Media and solvent control peaks were detected using the same approach (noise level: 1000; peak height: 3000) and subsequently removed from the filtered peak list. Filtered peak lists were subjected to statistical analyses to assess the chemical distinctiveness of the microbial extracts. Therefore, PCoA plots reflecting the metabolomic (dis)similarities of the selected extracts and ANOSIM scores (Euclidean distance) were calculated in Past v3.12 [99].

#### 4.5.3. Feature-Based Molecular Networking and Dereplication

Processed MS/MS data of prioritized extracts (see Section 2.3.) were submitted in MGF format to the FBMN workflow [32] available at the open access platform GNPS [34]. FBMNs were compiled as previously described [31] and visualized with Cytoscape v3.7.1 [100]. 

Compounds showing distinct peaks in the LC-MS chromatograms above the noise threshold were subjected to a dereplication workflow combining automated and manual dereplication tools. The prediction of putative molecular formulae was performed in MassLynx v4.1 (Waters). Four NP databases, i.e., Dictionary of Natural Products (http://dnp.chemnetbase.com), MarinLit (http://pubs.rsc.org/marinlit/), The Natural Products Atlas (https://www.npatlas.org/joomla/index.php/search/basic-search [101]) and Reaxys (https://www.reaxys.com), were inspected for putative hits for the predicted molecular formulae. In parallel, pre-processed MS/MS data were subjected to the dereplication workflow of GNPS [34]. The same dataset was also automatically dereplicated by applying the in silico MS/MS database of the Universal Natural Product Database [33]. Putatively annotated NPs were verified by comparing the biological sources, R_t_, and MS/MS spectra (if detected), of which the latter was aided by the in-silico prediction tool CFM-ID 3.0 [102].

## Figures and Tables

**Table 1:** IC_50_ values (in µg/mL) of selected extracts against eight microbial human pathogens. MRSA: methicillin-resistant *S. aureus*; Efm: *E. faecium*; Ab: *A. baumannii*; Ec: *E. coli*; Kp: *K. pneumoniae*; Psa: *P. aeruginosa*; Ca: *C. albicans*; Cn: *C. neoformans*. Positive controls: chloramphenicol (MRSA, Ec, Kp), ampicillin (Efm), doxycycline (Ab), polymyxin B (Psa), nystatin (Ca), and amphotericin (Cn).

**Extract**	**Identification**	**MRSA**	**Efm**	**Ab**	**Ec**	**Kp**	**Psa**	**Ca**	**Cn**
CHG48-GYM	*Streptomyces* sp.	5.0	4.5	>100	>100	>100	>100	12.9	13.1
CKG20-GYM	*Micromonospora* sp.	10.3	0.1	>100	>100	>100	>100	>100	>100
CKG24-GYM	*Bacillus* sp.	0.4	2.0	>100	>100	>100	>100	>100	17.1
CHG34-CAG	*Trichoderma* sp.	>100	>100	>100	>100	>100	>100	>100	>100
CHG34-PDA	*Trichoderma* sp.	36.8	32.0	>100	>100	>100	>100	3.7	58.8
CHG38-CAG	*Fusarium* sp.	4.0	3.0	>100	>100	>100	>100	9.9	20.9
CHG38-PDA	*Fusarium* sp.	10.8	7.6	>100	>100	>100	>100	11.4	>100
CKG23-CAG	*Penicillium* sp.	19.8	29.8	4.9	15.6	8.9	15.8	>100	>100
CKG23-PDA	*Penicillium* sp.	8.3	18.1	42.5	41.0	31.4	42.6	>100	>100
Positive control		3.1	0.4	0.02	1.4	0.4	0.4	1.3	0.1

**Table 2:** IC_50_ values (µg/mL) of selected extracts against four cancer cell lines. A375: malignant melanoma cell line; A549: lung carcinoma cell line; HCT116: colon cancer cell line; MB231: breast cancer cell line. Positive control: doxorubicin.

**Extract**	**Identification**	**A375**	**A549**	**HCT116**	**MB231**
CHG48-GYM	*Streptomyces* sp.	5.8	0.02	21.4	22.1
CKG20-GYM	*Micromonospora* sp.	0.8	1.6	1.3	1.4
CKG24-GYM	*Bacillus* sp.	70.7	67.9	86.6	>100
CHG34-CAG	*Trichoderma* sp.	67.9	70.1	71.9	69.7
CHG34-PDA	*Trichoderma* sp.	19.3	31.1	24.1	32.8
CHG38-CAG	*Fusarium* sp.	35.9	70.1	62.1	>100
CHG38-PDA	*Fusarium* sp.	92.3	>100	>100	>100
CKG23-CAG	*Penicillium* sp.	2.0	4.9	8.5	2.5
CKG23-PDA	*Penicillium* sp.	5.2	17.5	27.6	9.0
Positive control		0.8	1.3	13.3	2.3

**Table 3:** Summary of the chemical inventory of nine selected crude extracts explored by untargeted FBMN-based metabolomics. Each extract is given with the number of nodes detected in the global bacterial (Figure 6) or fungal (Figure 7) FBMNs. In addition, putatively identified chemical families and annotation rates are indicated for each strain.

**Extract**	**Identification**	**Nodes**	**Putatively Annotated Chemical Families**	**Annotation Rate (%)**
CHG48-GYM	*Streptomyces* sp.	73	Acetamides, diterpenoid glycosides, linear polyketides, nonactic acid polyketides, oxazolidone alkaloids	24
CKG20-GYM	*Micromonospora* sp.	61	Cyclic depsipeptides, phenazine alkaloids, glycerophospholipids	40
CKG24-GYM	*Bacillus* sp.	89	Cyclic lipopeptides, isocoumarins, polyketide glycosides	71
CHG34-CAG	*Trichoderma* sp.	173	Ergosterols, peptaibols, sorbicillinoids	27
CHG34-PDA	*Trichoderma* sp.	171		
CHG38-CAG	*Fusarium* sp.	53	Chromones, cyclic lipopeptides, isocoumarins, naphthoquinones, xanthones, zearalenones	38
CHG38-PDA	*Fusarium* sp.	54		
CKG23-CAG	*Penicillium* sp.	29	Cytochalasans, indole alkaloids, mero- and sesquiterpenoids, styrylpyrones, tetrapeptides, xanthones, zearalenones	48
CKG23-PDA	*Penicillium* sp.	108		

## Data Availability

The sequencing data presented in this study are openly available in GenBank at NCBI (accession numbers: MW065489-549, MW064137-74, MW064175-6). The metabolomics data presented in this study are available on request from the corresponding author. The data are not publicly available due to ongoing research on the novel chemistry of the presented microbial strains.

## Supplementary Materials

The following are available online at https://www.mdpi.com/1660-3397/19/1/6/s1, Figure S1: Venn diagram of exclusive and shared peaks of *N. prasina* and *Streptomyces* sp. extracts. Figure S2: Structures of putatively identified compounds. Figures S3–S8: FBMNs of prioritized crude extracts. Figures S9–S11: MS/MS spectra of putatively annotated compounds. Figure S12: Comparative metabolome analyses of *Penicillium* sp. isolate CKG23. Table S1: Taxonomic classification of microbial strains isolated from the gut of *C. intestinalis* sampled in Helgoland and Kiel Fjord. Table S2: Antimicrobial and anticancer activities of microbial crude extracts. Tables S3–S4: Statistical comparison of chemically distinct microbial crude extracts. Tables S5–S10: Putatively identified compounds in prioritized microbial crude extracts.
