# Supplementary material for: Diversity, Bioactivity Profiling and Untargeted Metabolomics of the Cultivable Gut Microbiota of Ciona intestinalis
Source: Mar Drugs. 2020 Dec 24;19(1):6. doi: 10.3390/md19010006 (PMC7824411; doi:10.3390/md19010006)
Supplement: Supplementary file 1 [file marinedrugs-19-00006-s001.pdf]

Supplementary Information

Diversity, Bioactivity Profiling and Untargeted Metabolomics of the Cultivable Gut Microbiota of *Ciona intestinalis*

Caroline Utermann [1], Vivien A. Echelmeyer [1], Ernest Oppong-Danquah [1], Martina Blümel [1], and Deniz Tasdemir [1,2*]

[1] GEOMAR Centre for Marine Biotechnology (GEOMAR-Biotech), Research Unit Marine Natural Products Chemistry, GEOMAR Helmholtz Centre for Ocean Research Kiel, Am Kiel-Kanal 44, 24106 Kiel, Germany
[2] Faculty of Mathematics and Natural Sciences, Kiel University, Christian-Albrechts-Platz 4, 24118 Kiel, Germany
* Corresponding author (dtasdemir@geomar.de)

Supplementary Figures

Figure S1. Venn diagram of exclusive and shared peaks of three *Streptomyces* sp. extracts (CHG40-GYM, CHG48-GYM, CHG64-GYM) and one *N. prasina* extract (CKG58-GYM).
Figure S2. Putatively identified compounds detected in crude extracts of microorganisms associated with the gut of *C. intestinalis*.
Figure S3. FBMN of *Streptomyces* sp. extract CHG48-GYM.
Figure S4. FBMN of *Micromonospora* sp. extract CKG20-GYM.
Figure S5. FBMN of *Bacillus* sp. extract CKG24-GYM.
Figure S6. FBMN of *Trichoderma* sp. extracts CHG34-CAG and CHG34-PDA.
Figure S7. FBMN of *Fusarium* sp. extracts CHG38-CAG and CHG38-PDA.
Figure S8. FBMN of *Penicillium* sp. extracts CKG23-CAG and CKG23-PDA.
Figure S9. Experimental (black) and library (green) MS/MS spectra of bonactin (**14**), putatively identified in *Streptomyces* sp. extract CHG48-GYM.
Figure S10. Experimental (black) and library (green) MS/MS spectra of homononactyl homononactate (**18**), putatively identified in *Streptomyces* sp. extract CHG48-GYM.
Figure S11. Annotated MS/MS spectra of putatively novel lipopeptides detected in *Trichoderma* sp. extracts CHG34-CAG and CHG34-PDA.
Figure S12. Comparative metabolome analyses of *Penicillium* sp. extracts CKG23-CAG and CKG23-PDA.

Supplementary Tables

Table S1. Taxonomic classification of microbial strains isolated from the gut of *C. intestinalis* sampled in Helgoland and Kiel Fjord.
Table S2. Antimicrobial and anticancer activities (% inhibition at a test concentration of 100 µg/mL) of microbial crude extracts.
Table S3. Statistical comparison of chemically distinct bacterial crude extracts.
Table S4. Statistical comparison of chemically distinct fungal crude extracts.
Table S5. Putatively identified compounds produced by *Streptomyces* sp. extract CHG48-GYM.
Table S6. Putatively identified compounds produced by *Micromonospora* sp. extract CKG20-GYM.
Table S7. Putatively identified compounds produced by *Bacillus* sp. extract CKG24-GYM.
Table S8. Putatively identified compounds produced by *Trichoderma* sp. extracts CHG34-CAG and CHG34-PDA.
Table S9. Putatively identified compounds produced by *Fusarium* sp. extracts CHG38-CAG and CHG38-PDA.
Table S10. Putatively identified compounds produced by *Penicillium* sp. extracts CKG23-CAG and CKG23-PDA.

Supplementary References 1-65

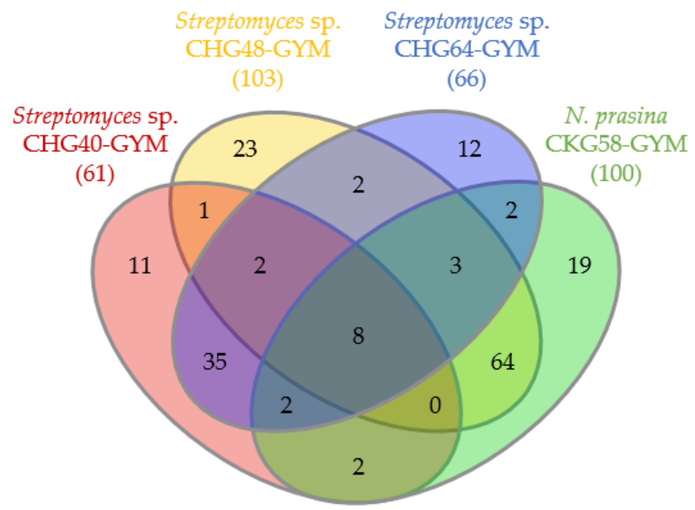

Figure S1. Venn diagram of exclusive and shared peaks of three *Streptomyces* sp. extracts (CHG40-GYM, CHG48-GYM, CHG64-GYM) and one *N. prasina* extract (CKG58-GYM).

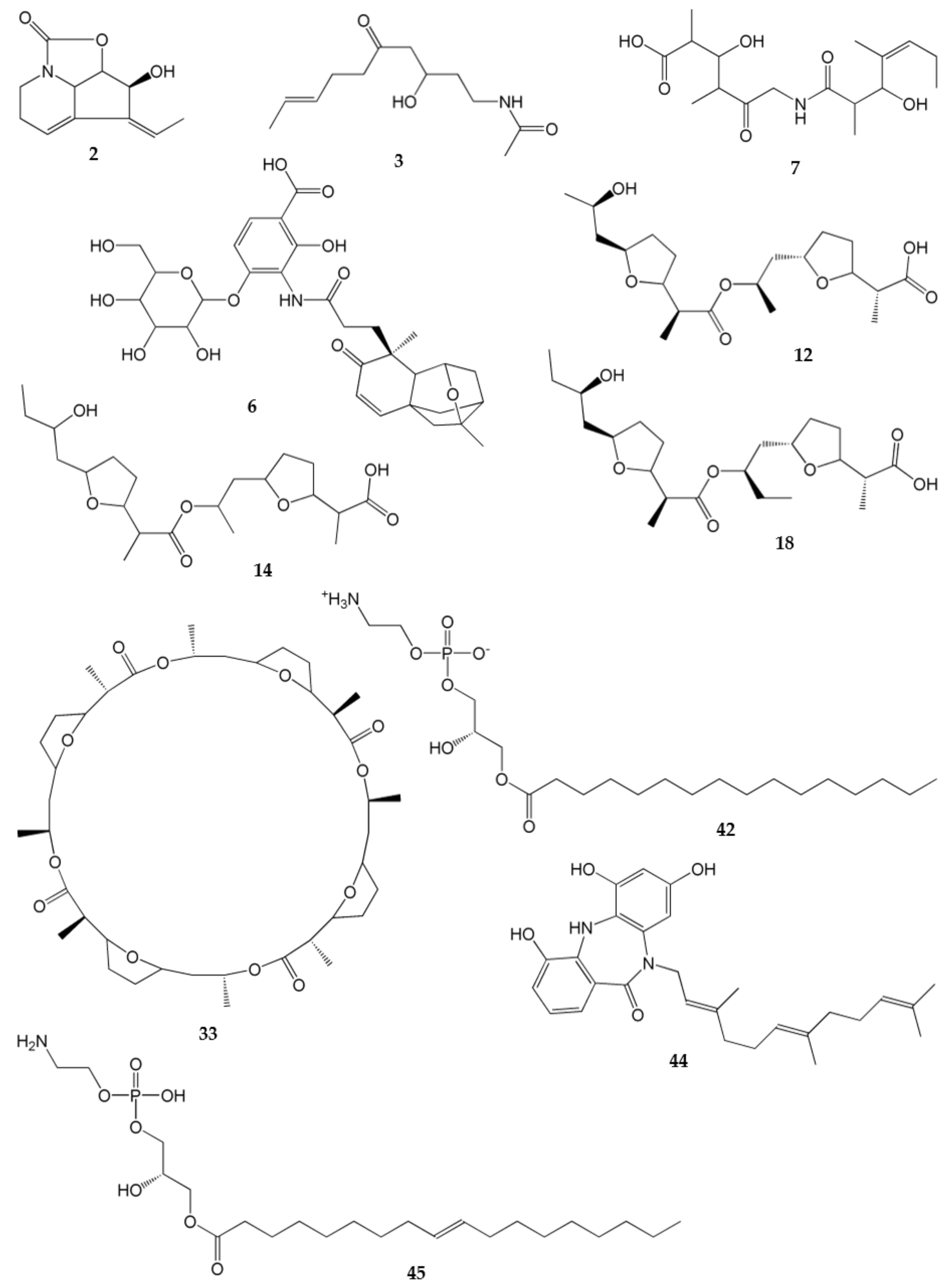

Figure S2. Putatively identified compounds detected in crude extracts of microorganisms associated with the gut of *C. intestinalis*. Chemical structures are labelled with their respective peak number (see Tables S5-S10).

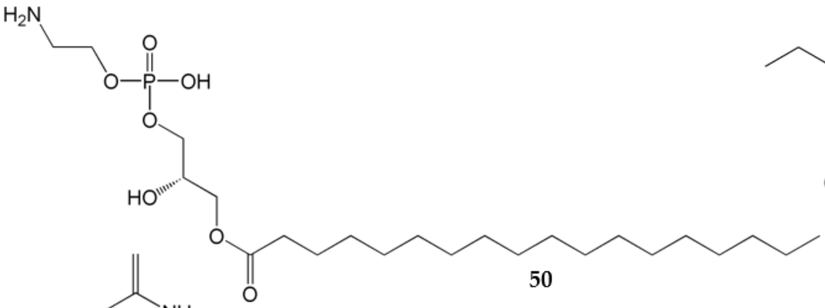

50

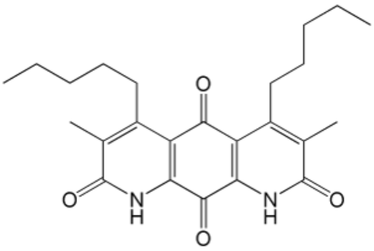

48

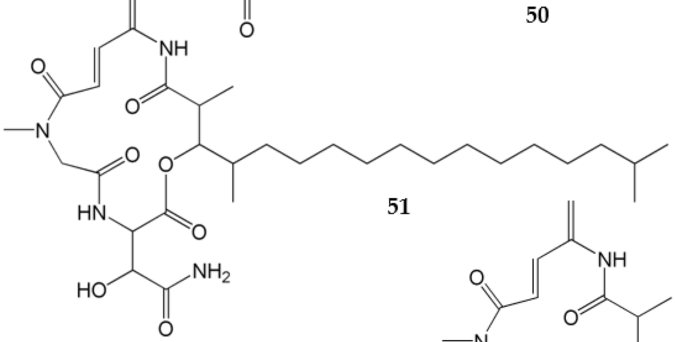

51

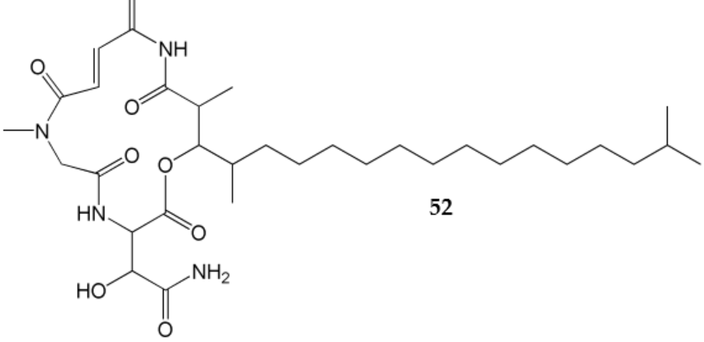

52

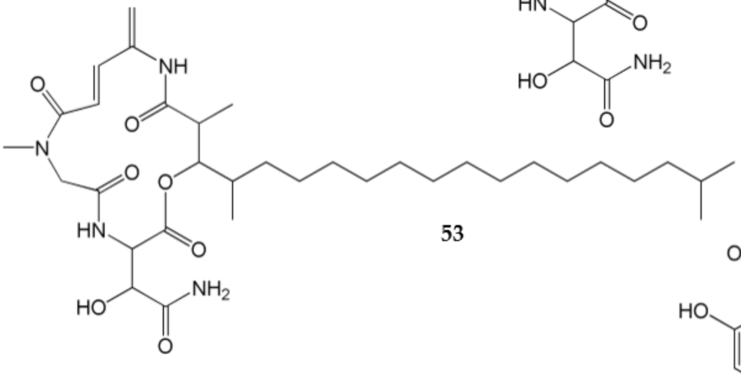

53

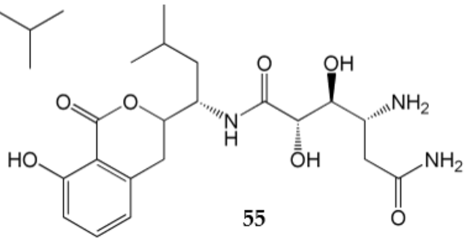

55

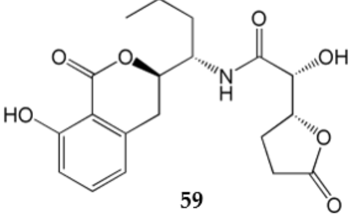

59

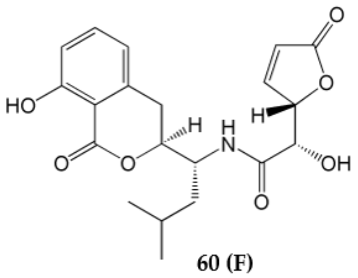

60 (F)

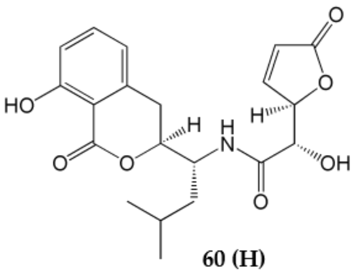

60 (H)

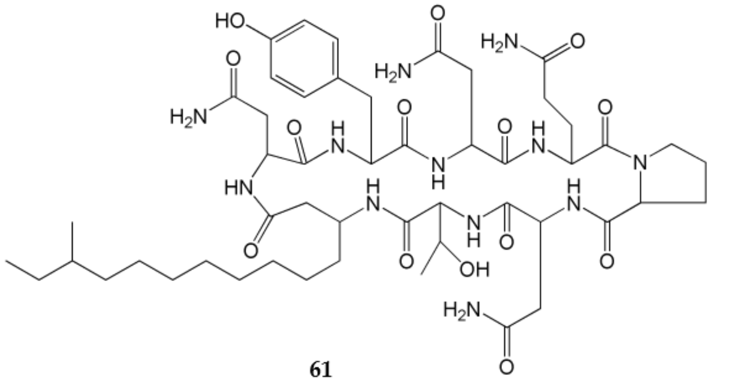

61

Figure S2. (continued)

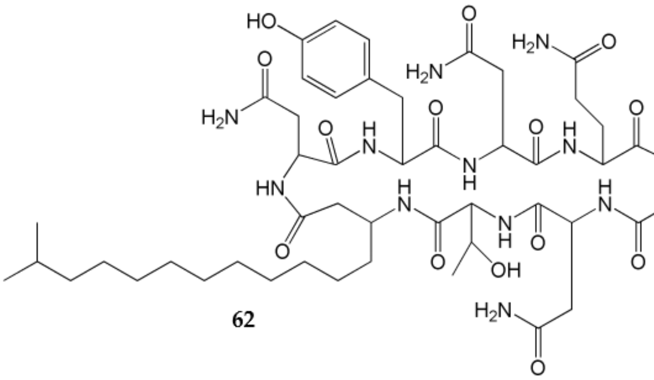

62

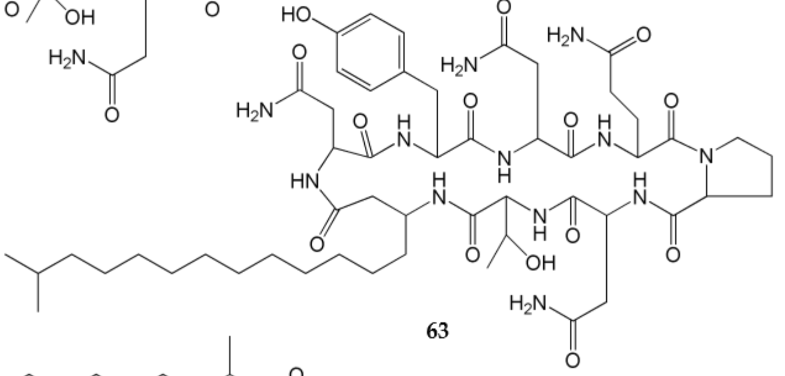

63

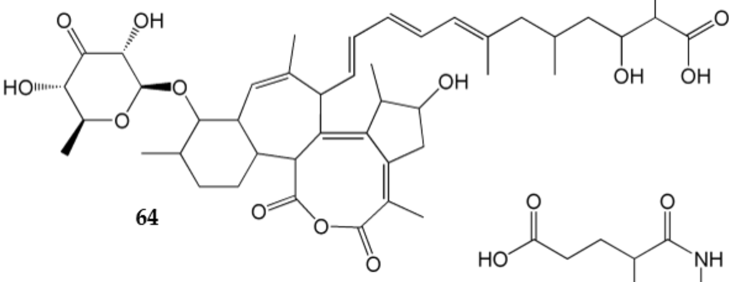

64

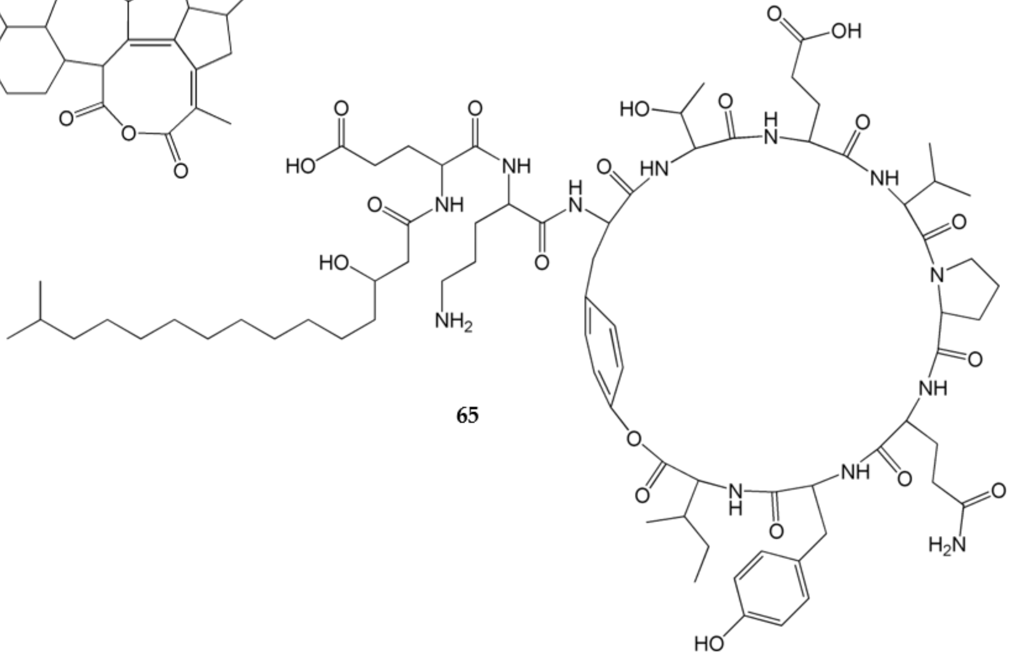

65

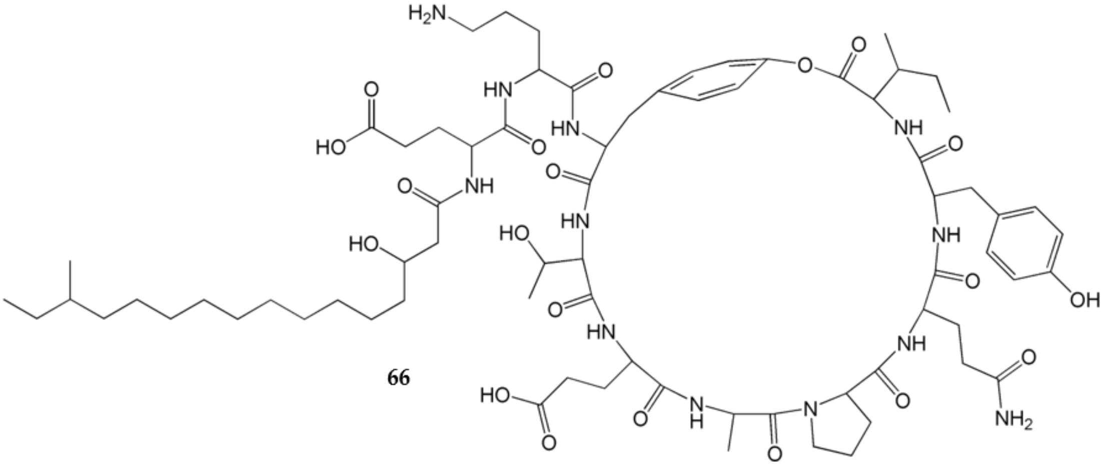

66

Figure S2. (continued)

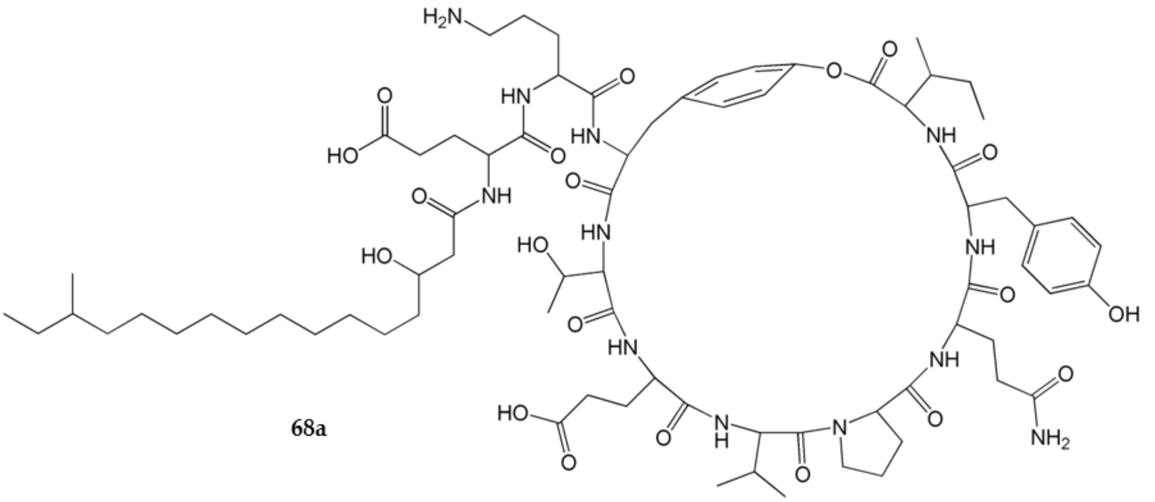

68a

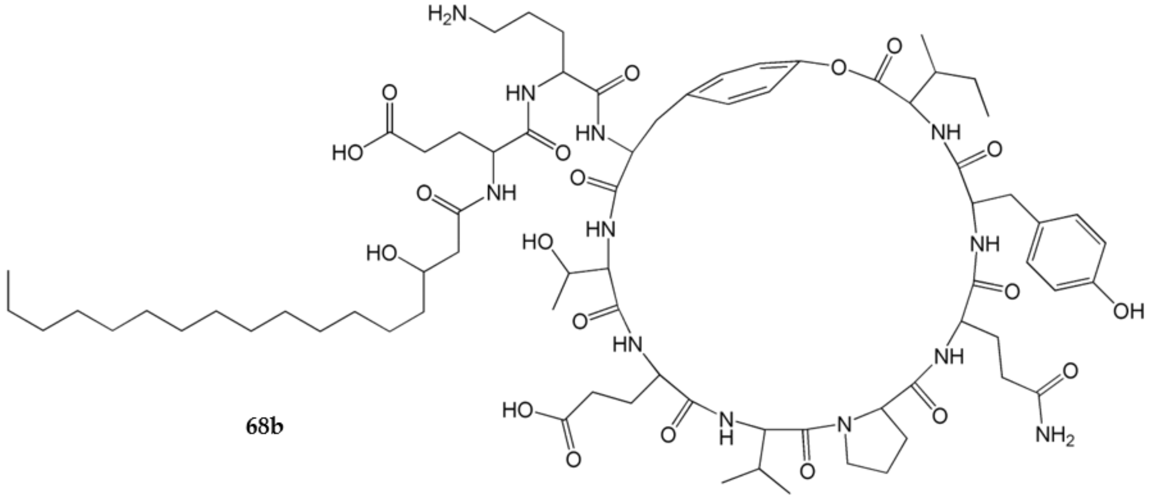

68b

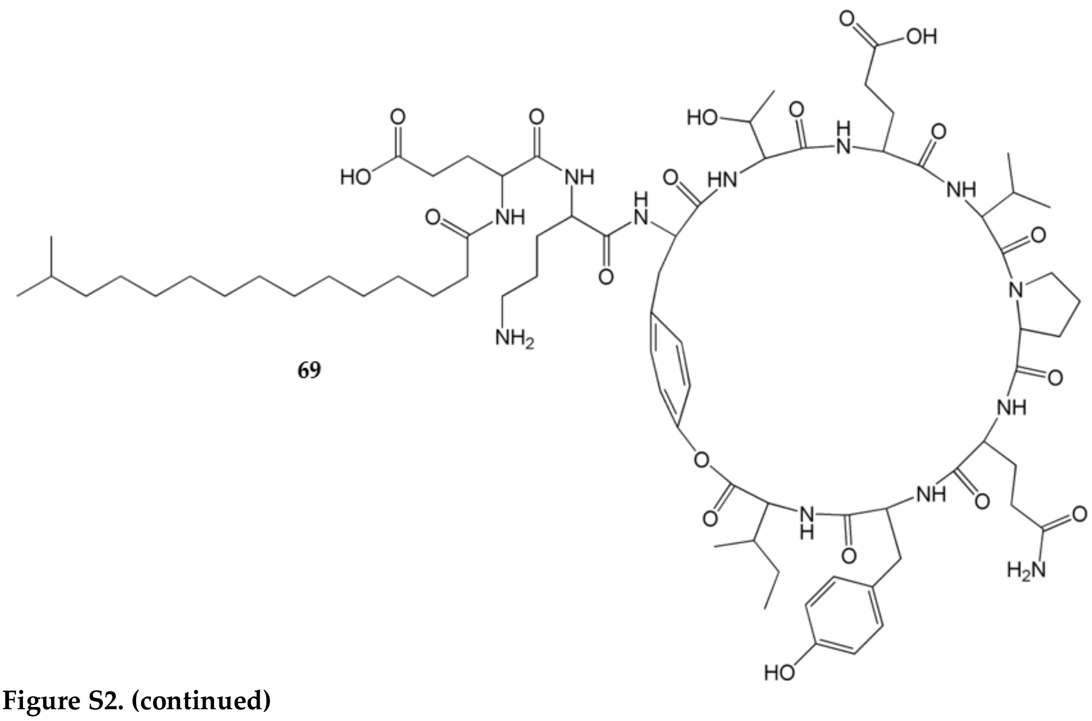

69

Figure S2. (continued)

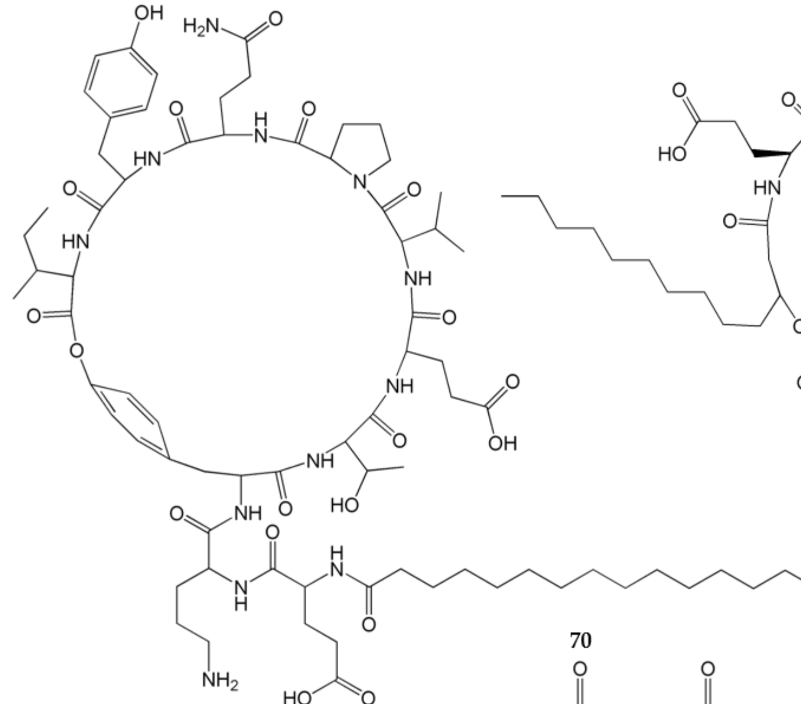

70

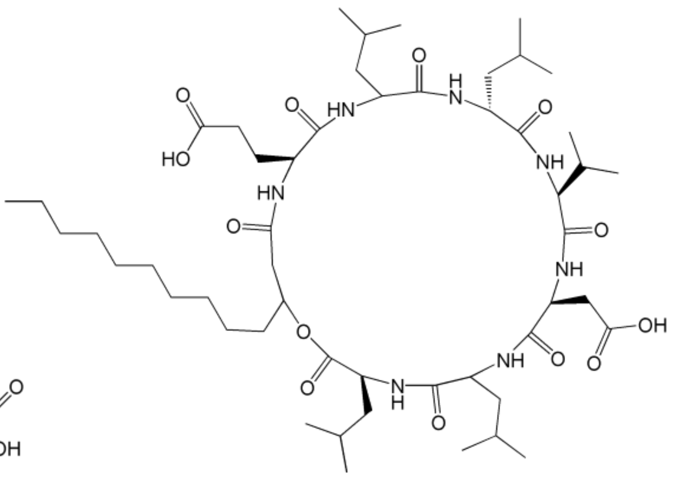

74

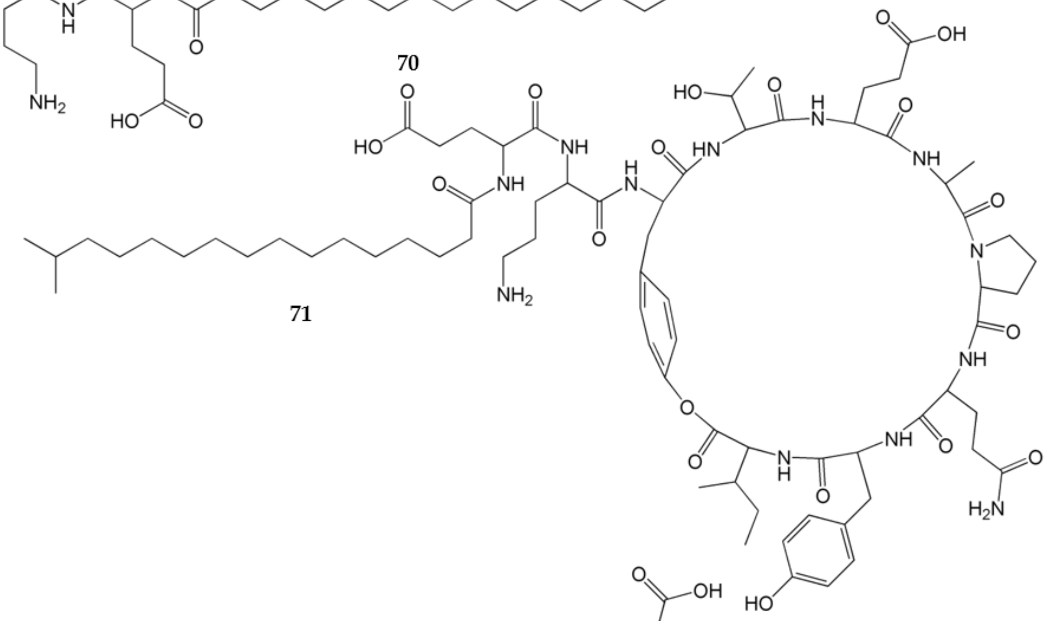

71

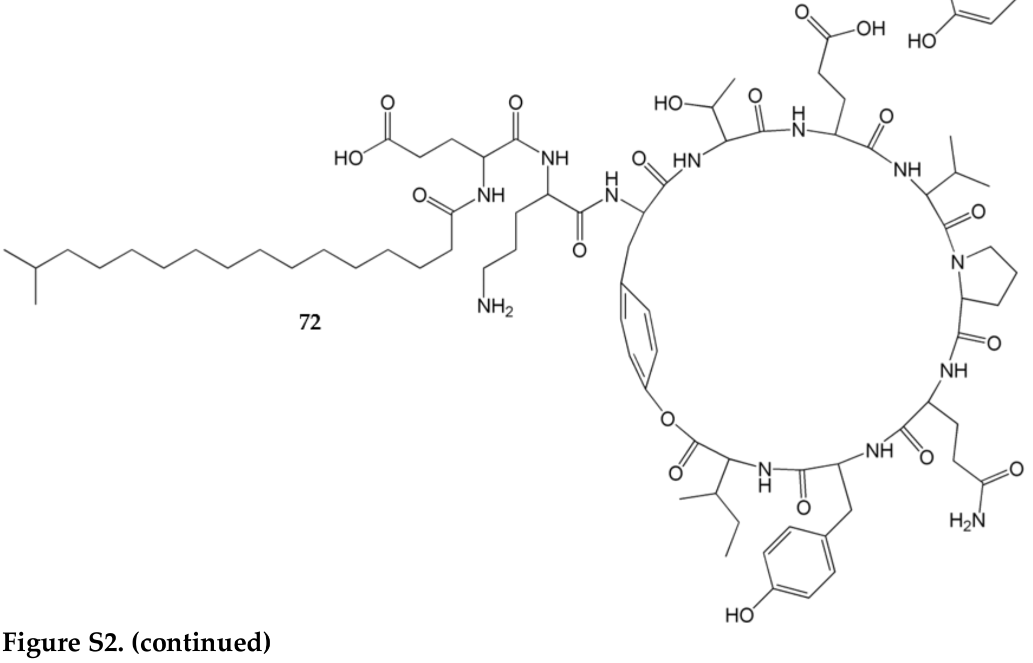

72

Figure S2. (continued)

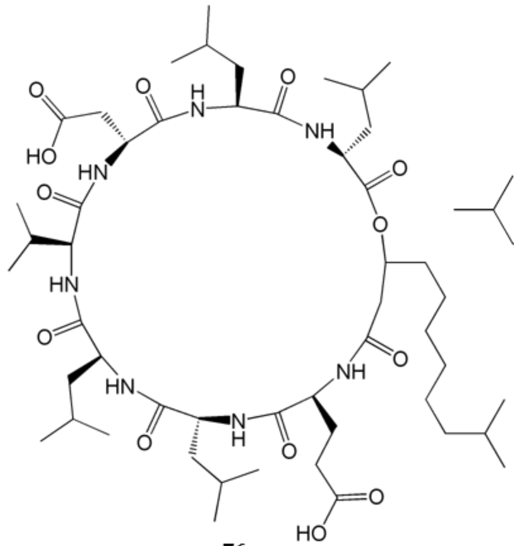

76

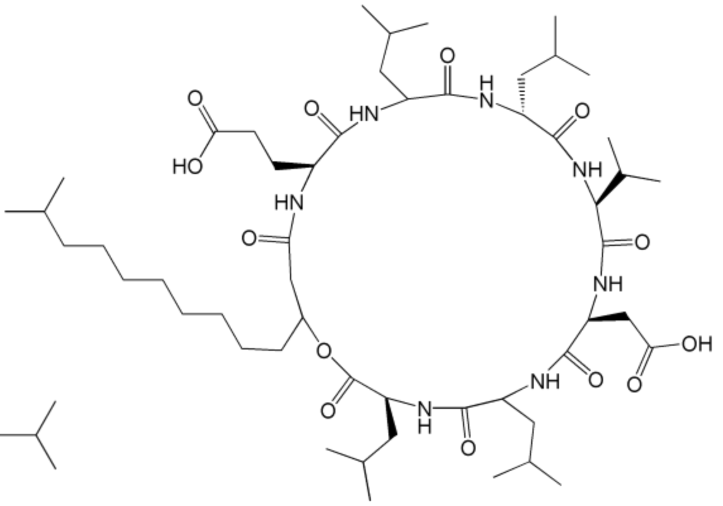

77

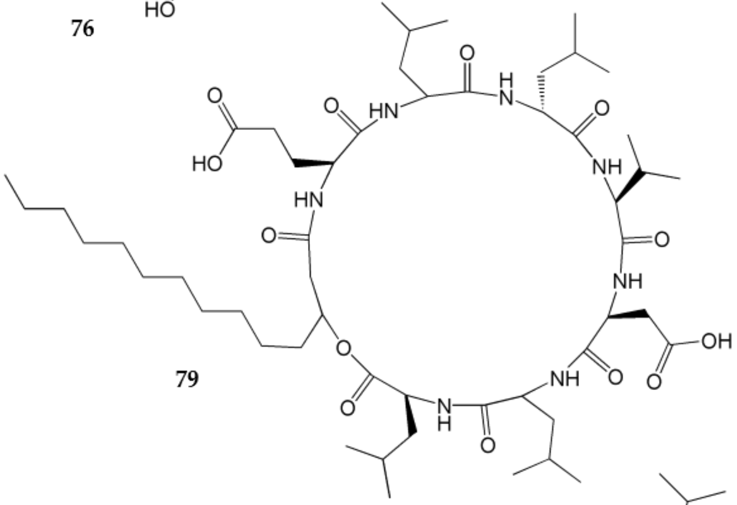

79

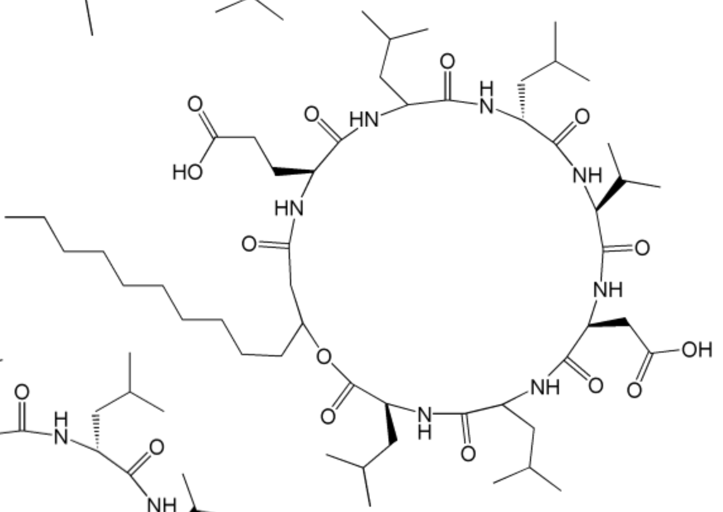

81

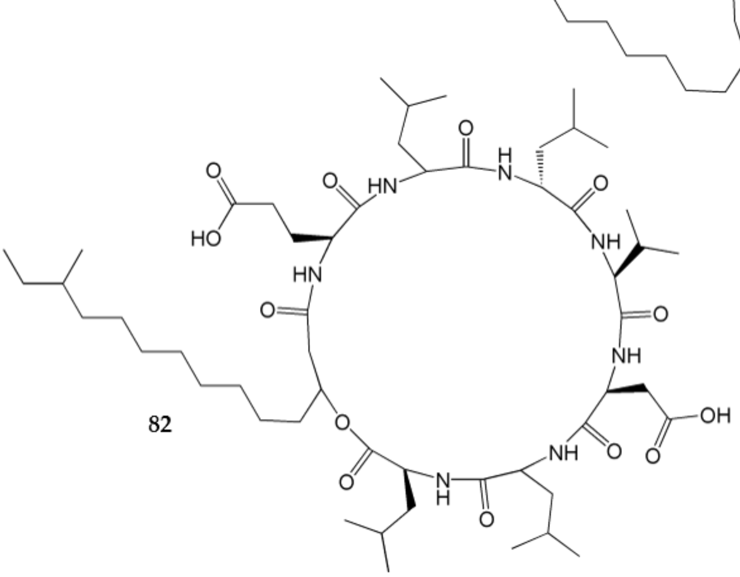

82

Figure S2. (continued)

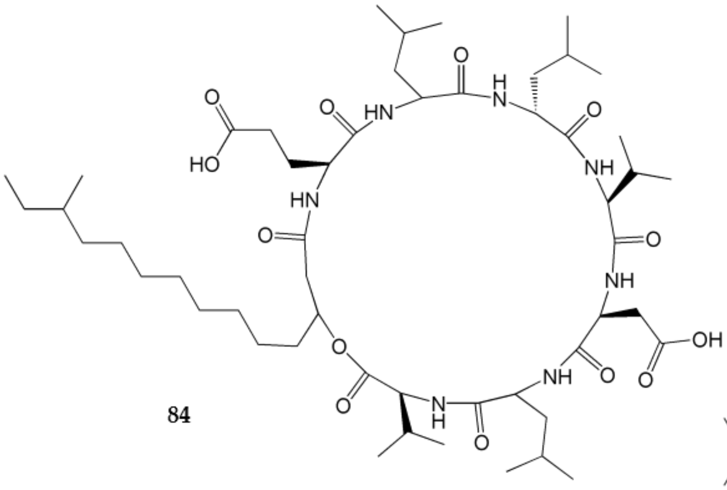

84

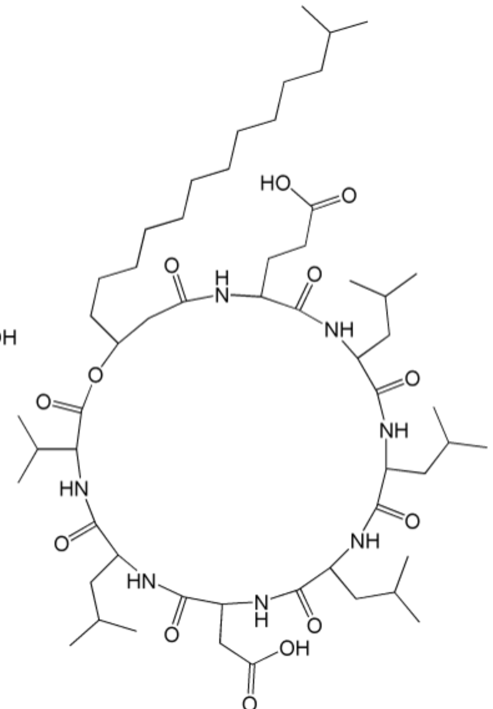

87

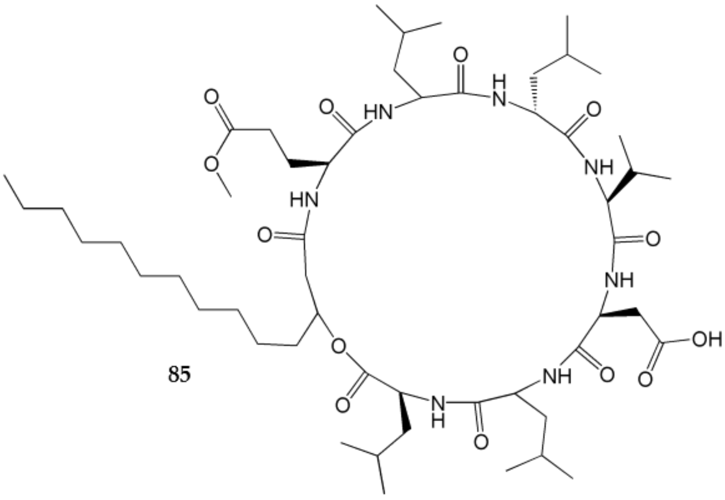

85

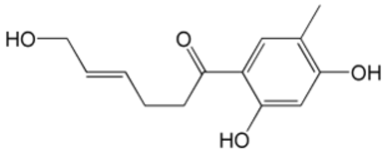

88

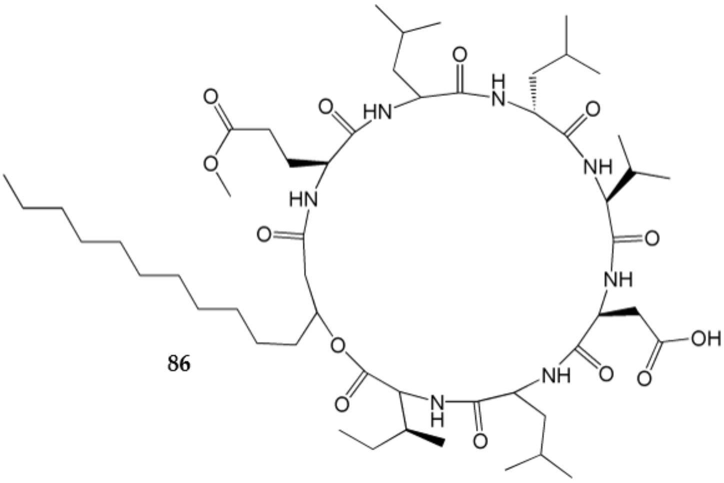

86

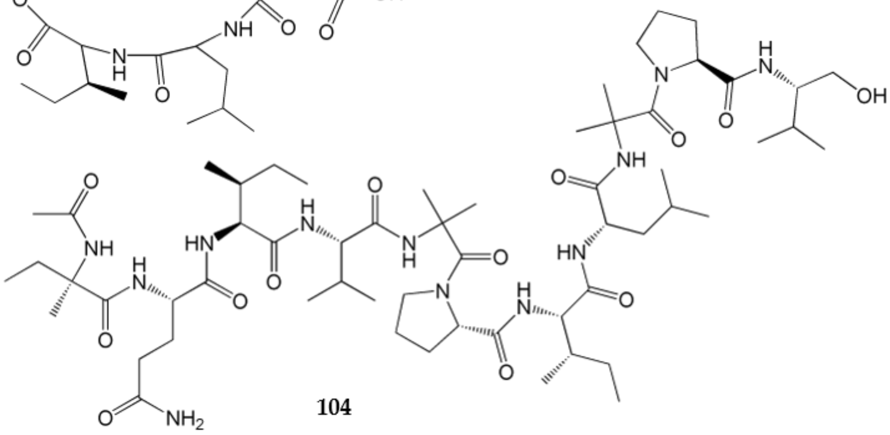

104

Figure S2. (continued)

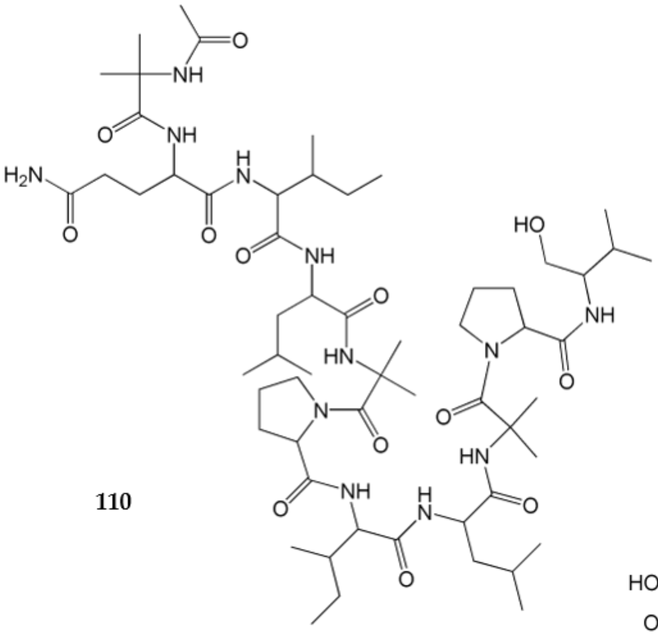

110

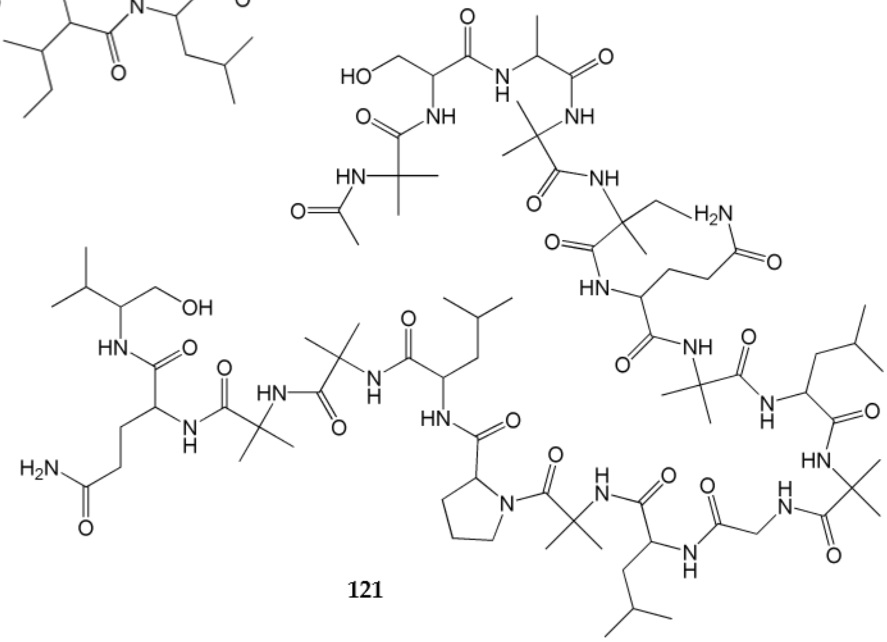

121

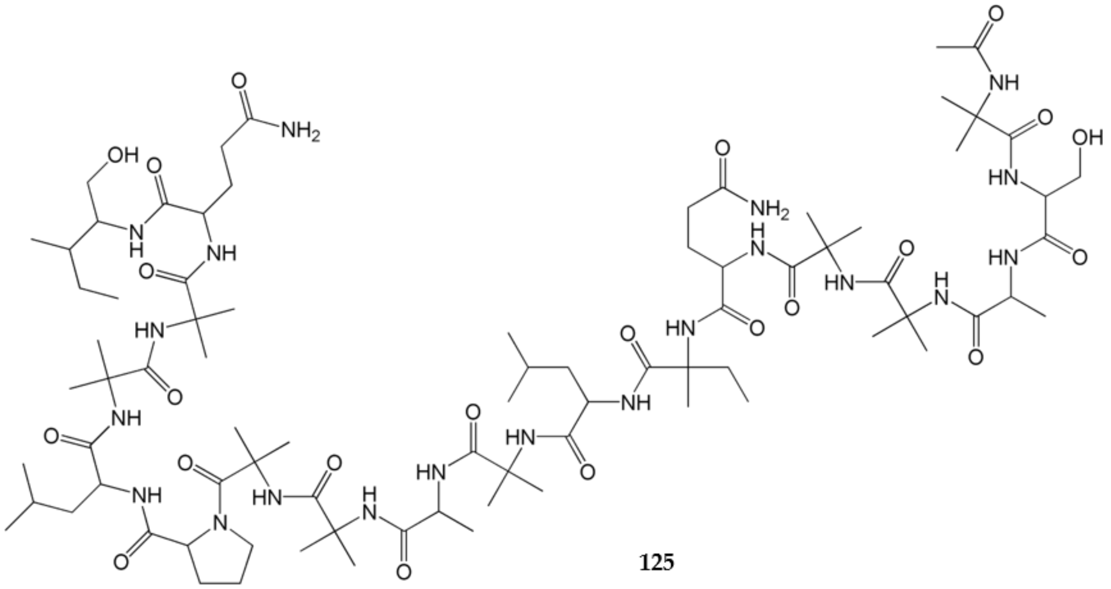

125

Figure S2. (continued)

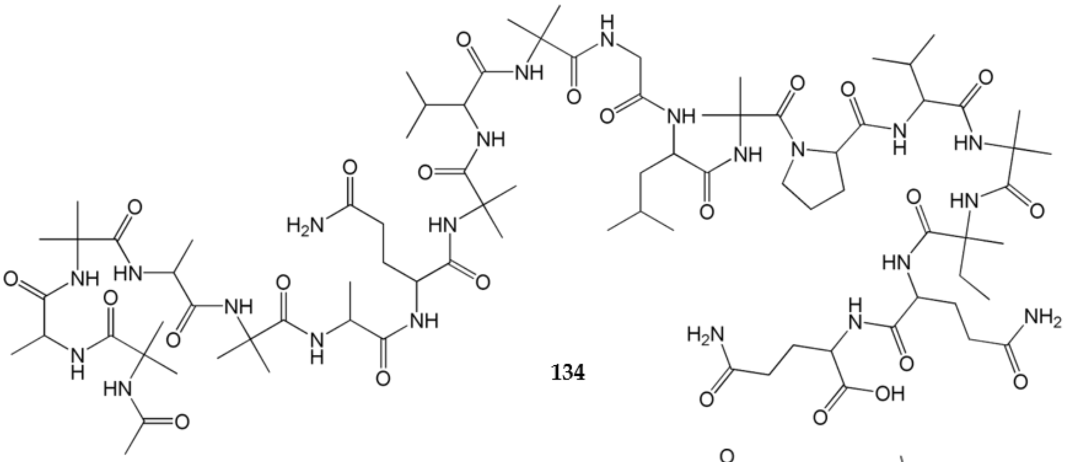

134

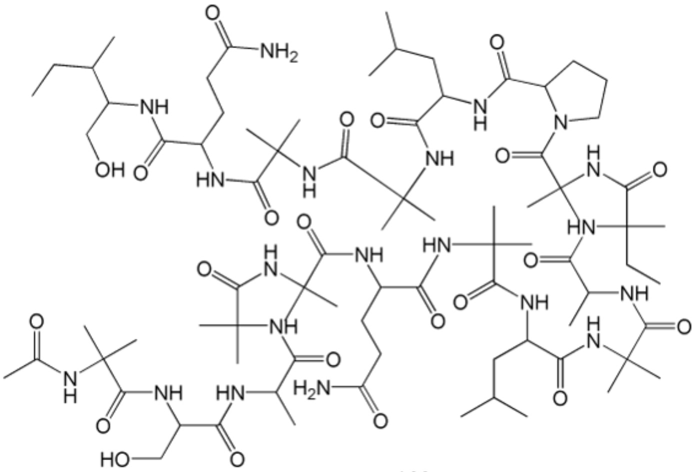

144

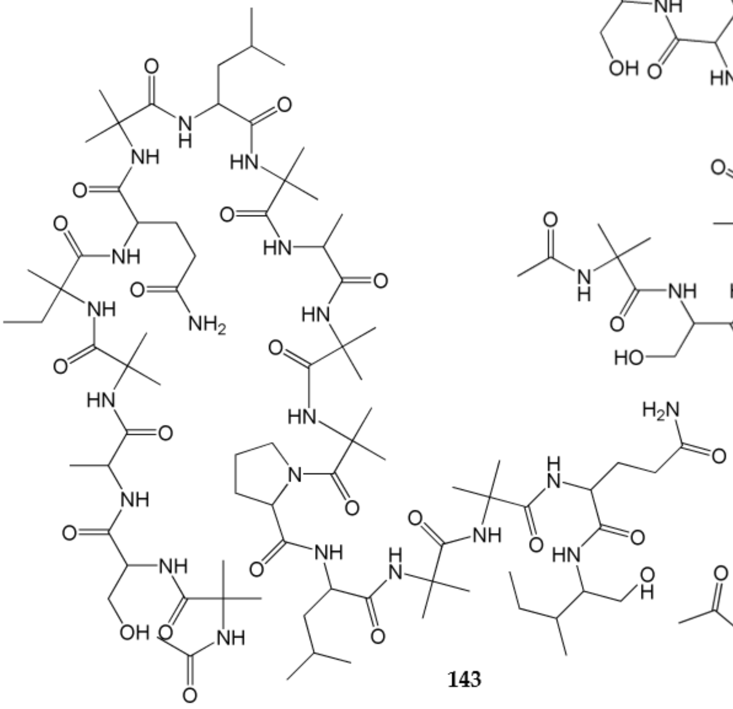

143

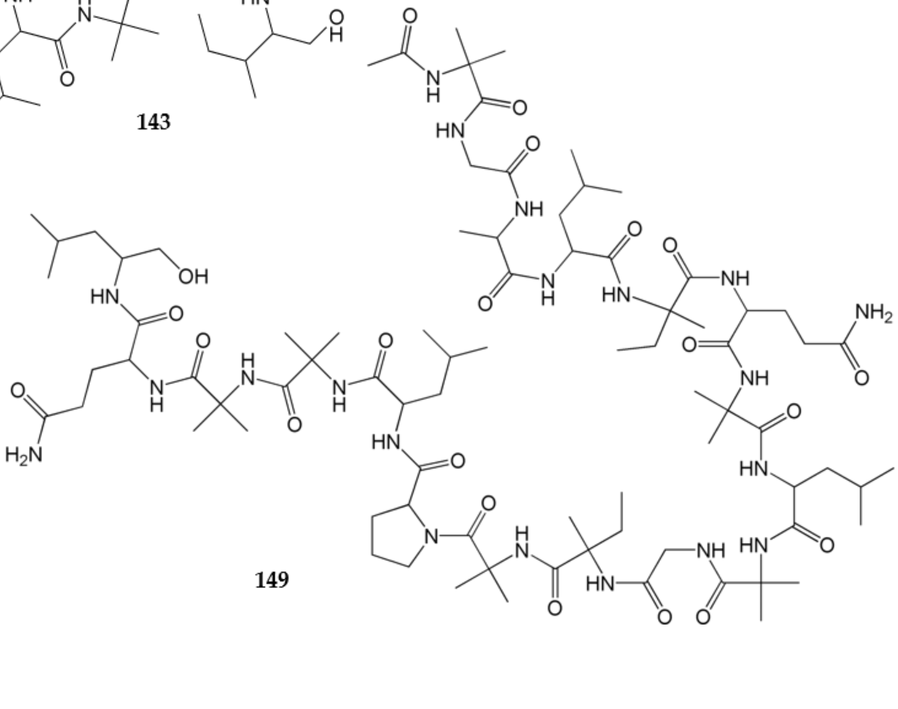

149

Figure S2. (continued)

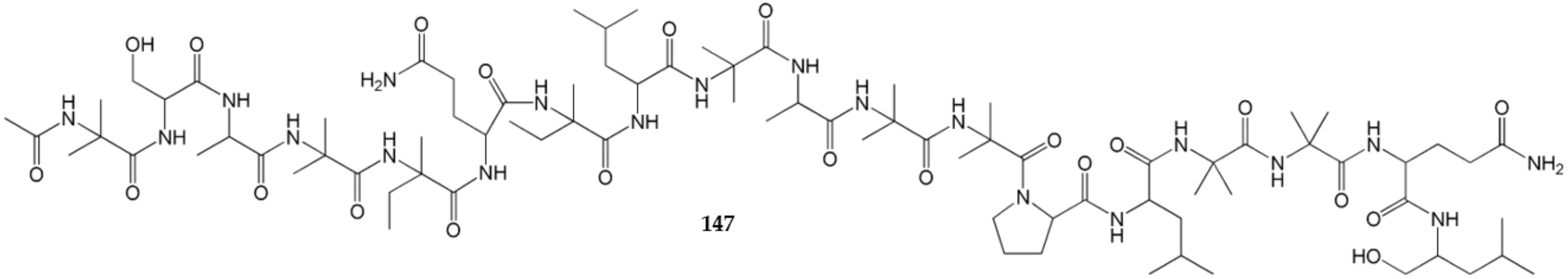

147

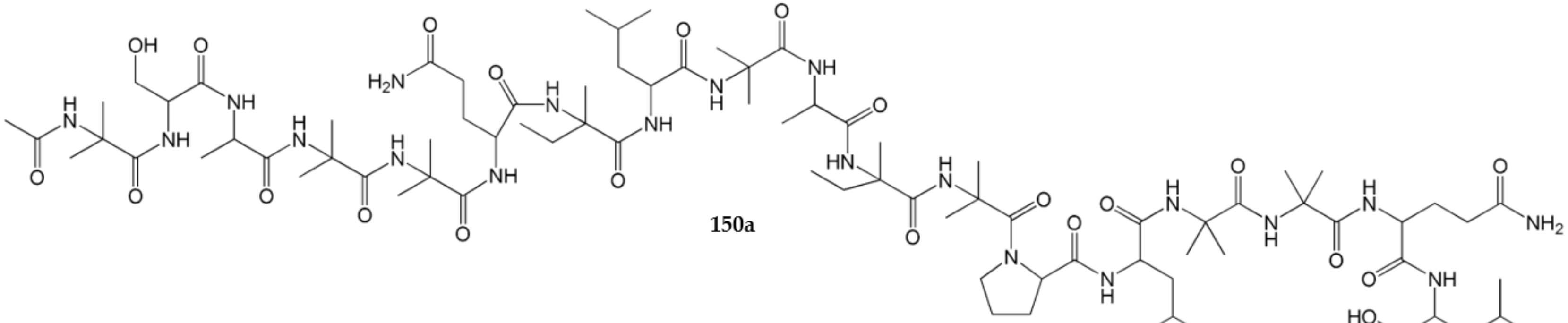

150a

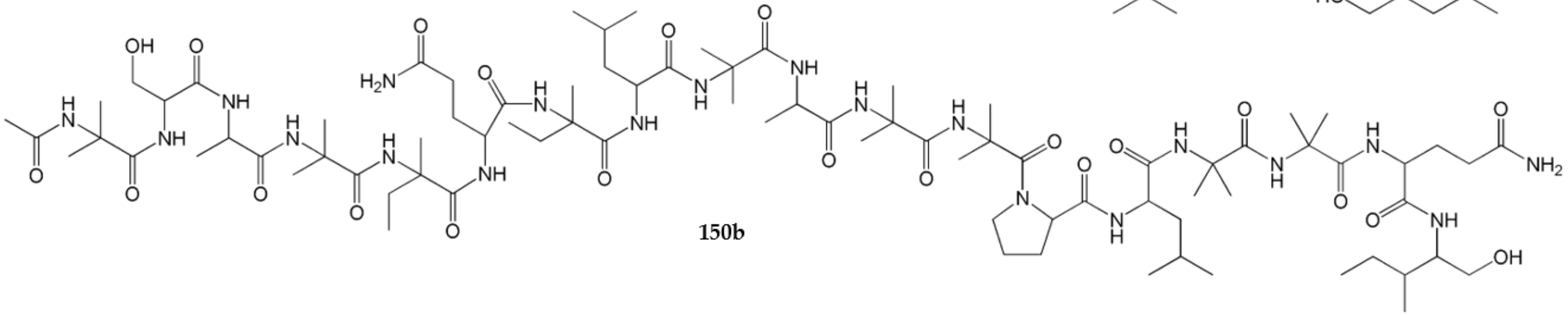

150b

Figure S2. (continued)

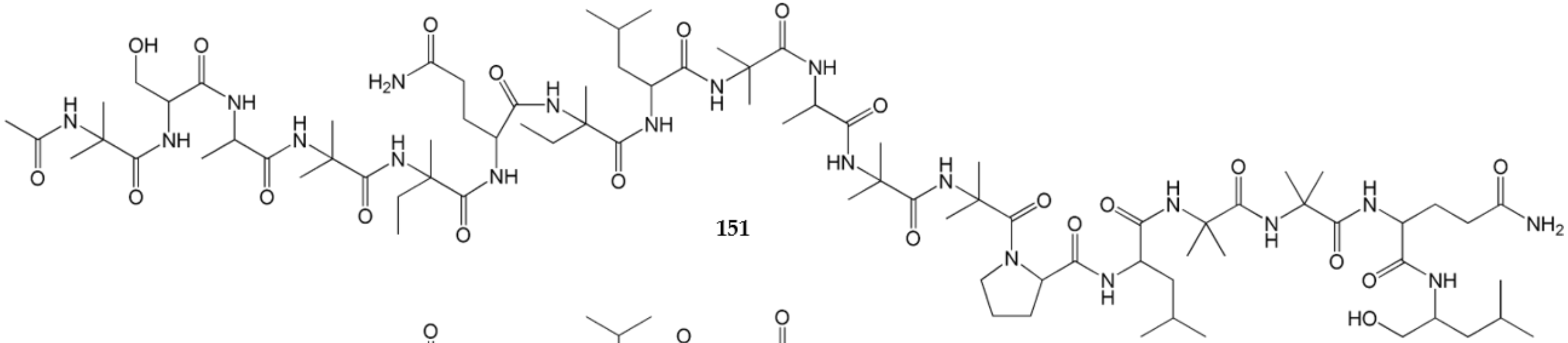

151

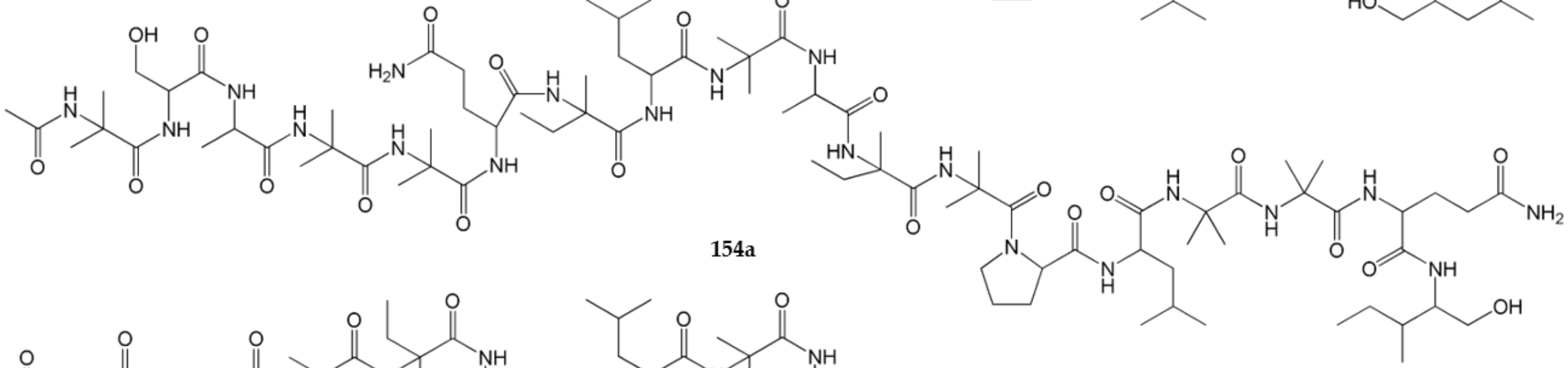

154a

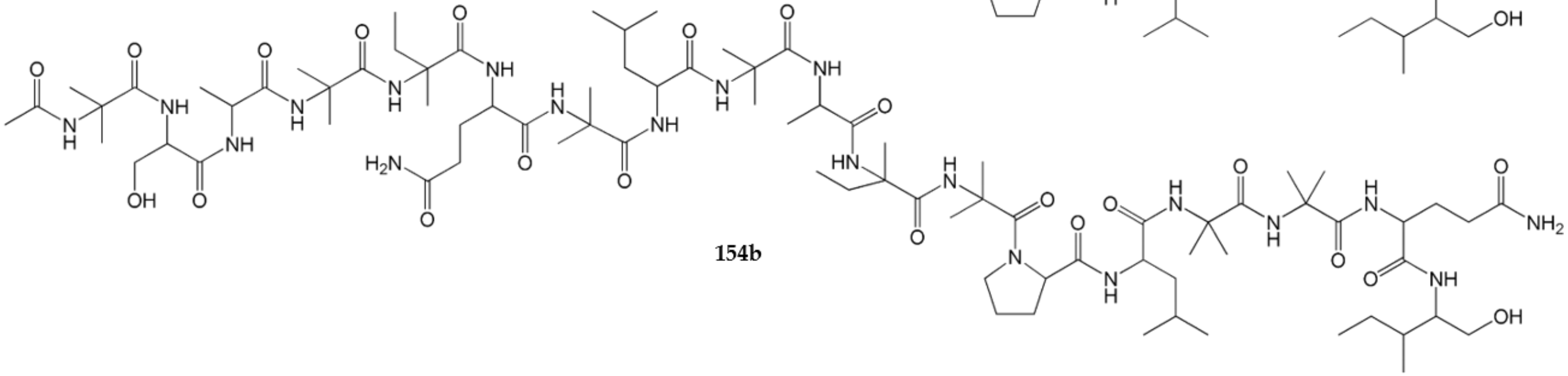

154b

Figure S2. (continued)

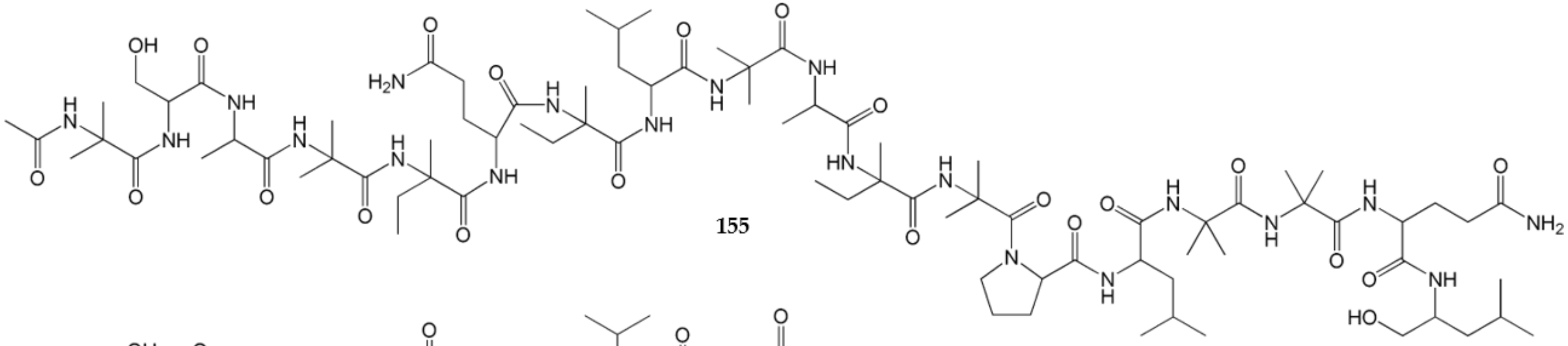

155

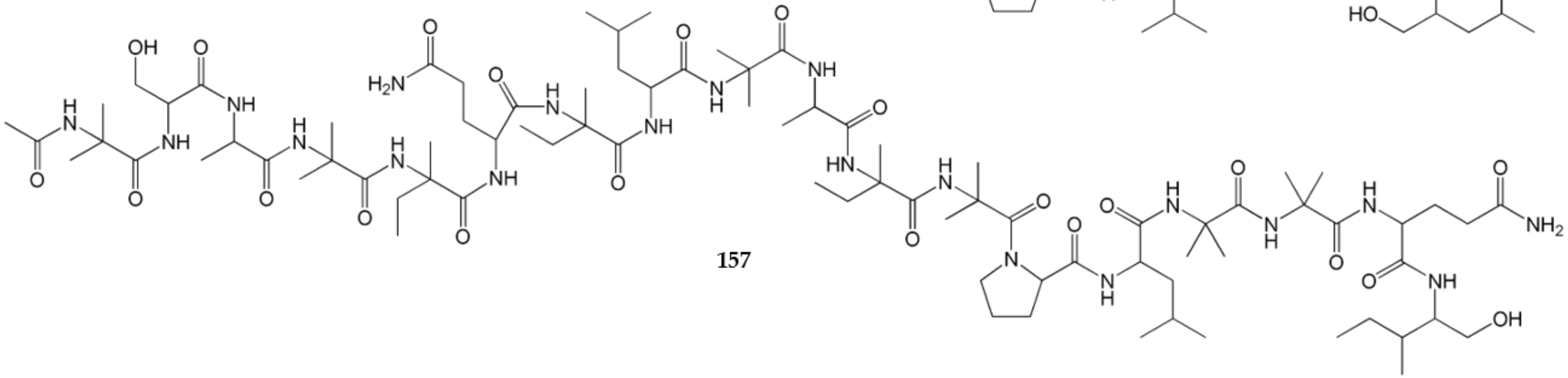

157

Figure S2. (continued)

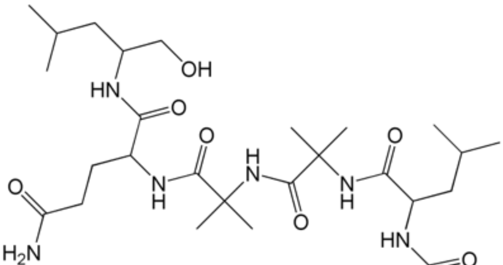

156

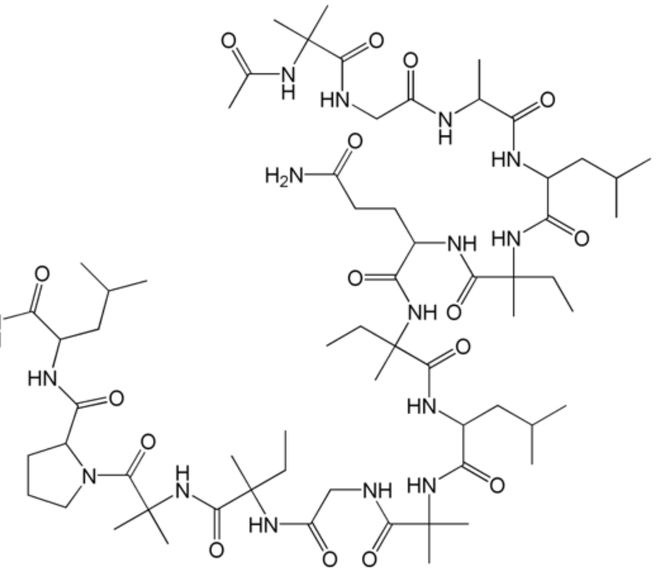

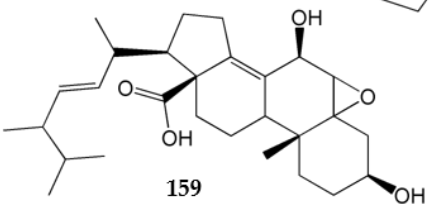

159

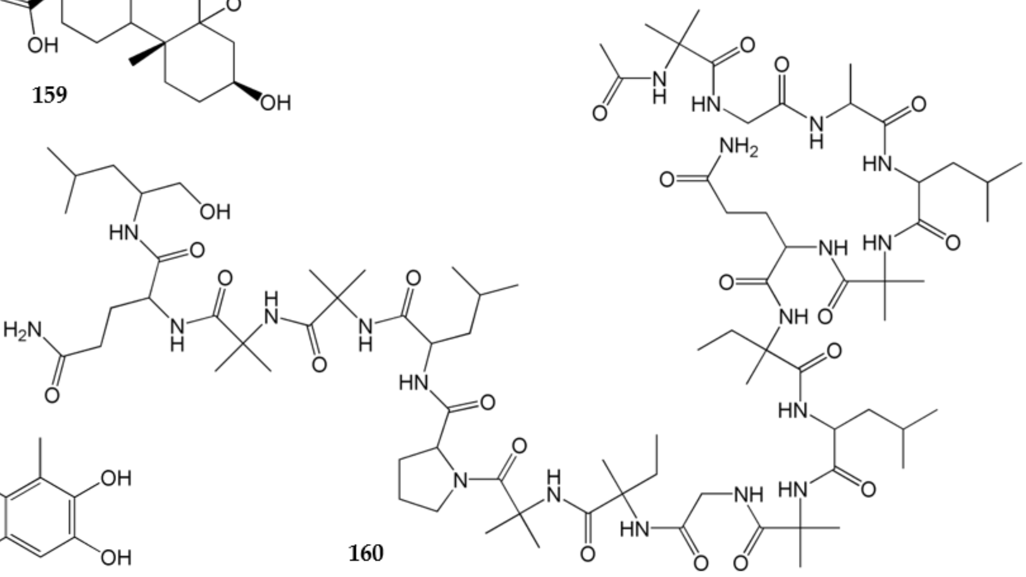

160

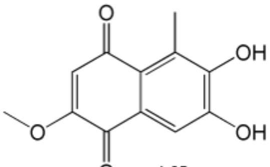

162

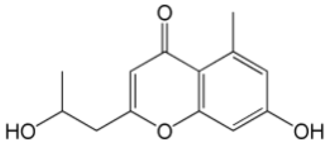

163

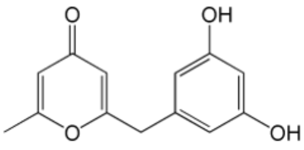

164

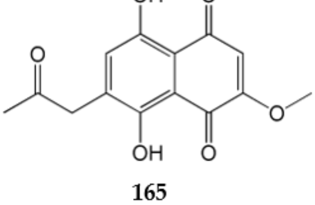

165

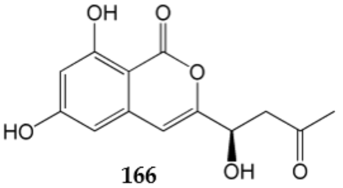

166

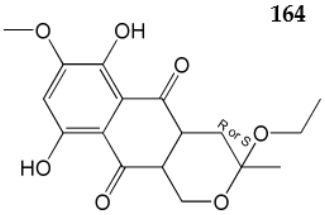

168

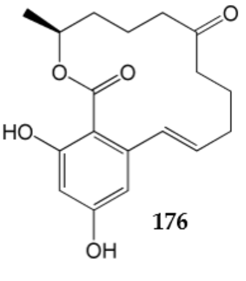

176

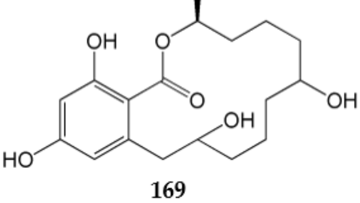

169

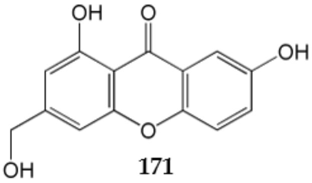

171

Figure S2. (continued)

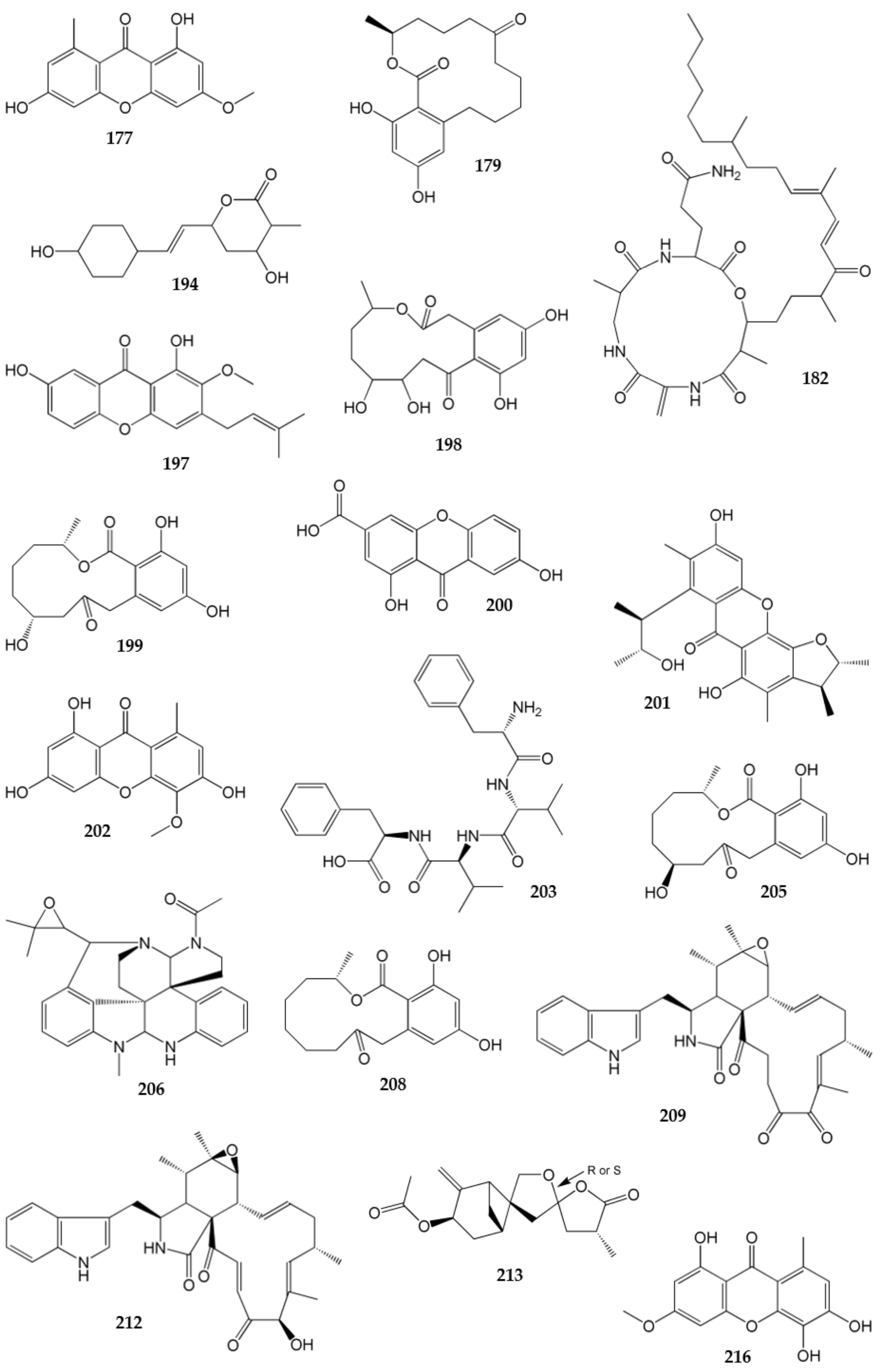

Figure S2. (continued)

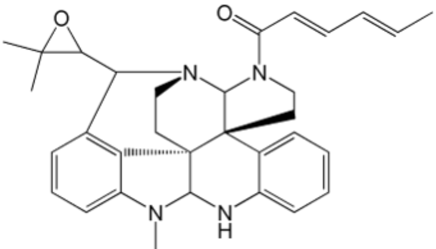

219

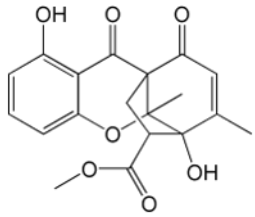

221

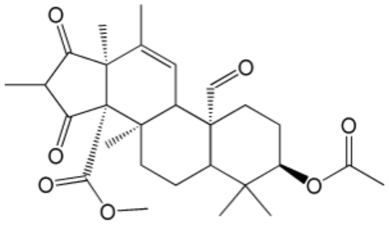

222

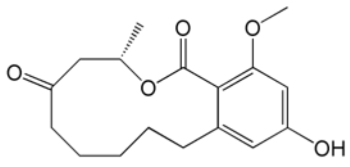

224a

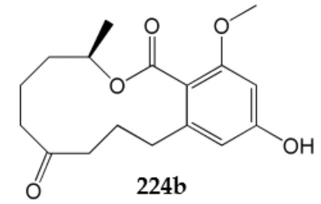

224b

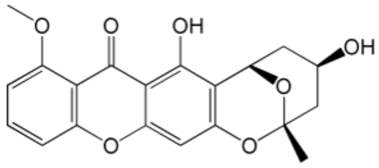

232

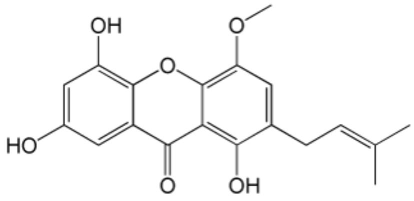

236

Figure S2. (continued)

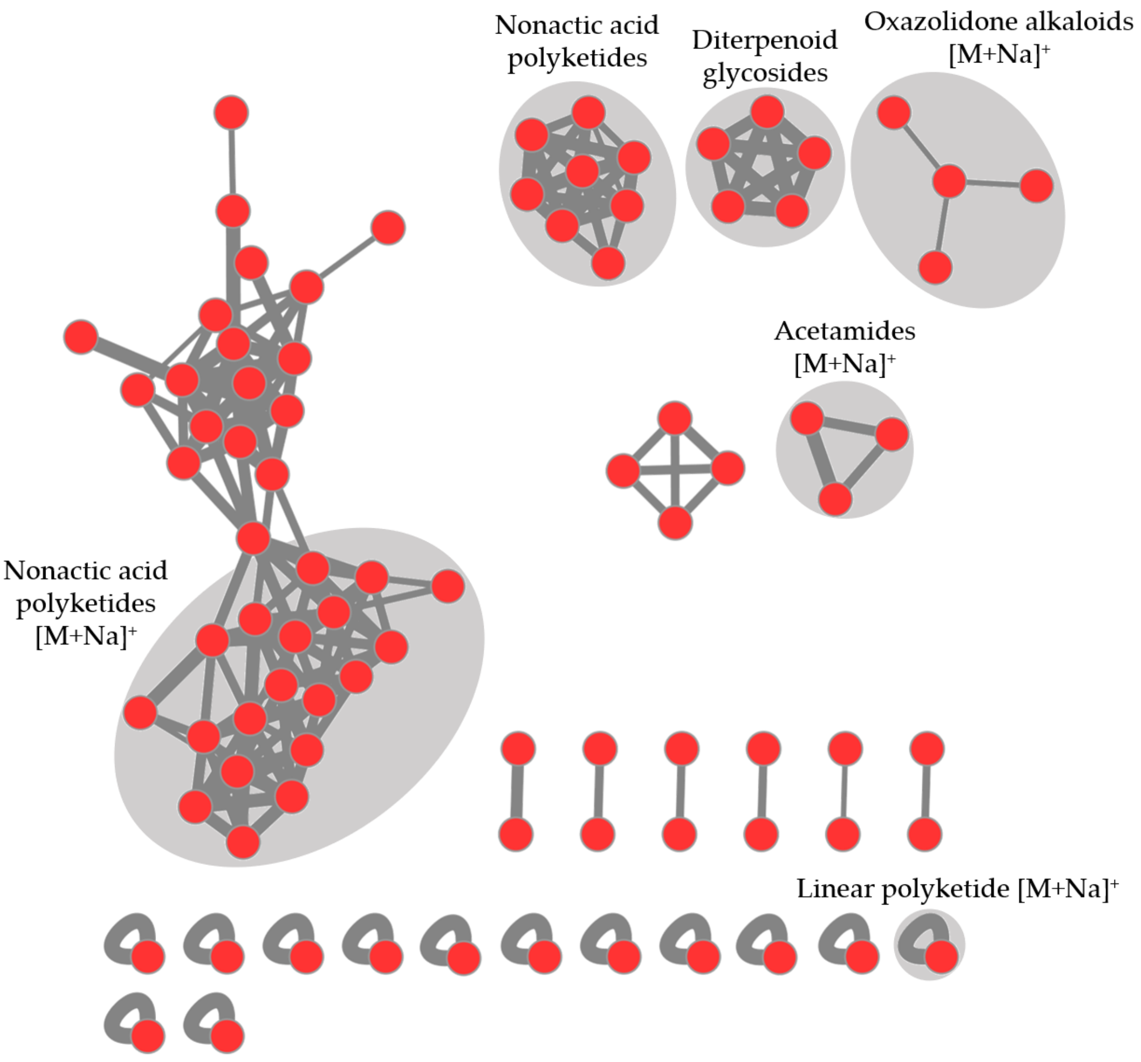

Figure S3. FBMN of *Streptomyces* sp. extract CHG48-GYM. The width of edges represents the cosine similarity between two nodes. See Table S5 for putatively annotated compounds.

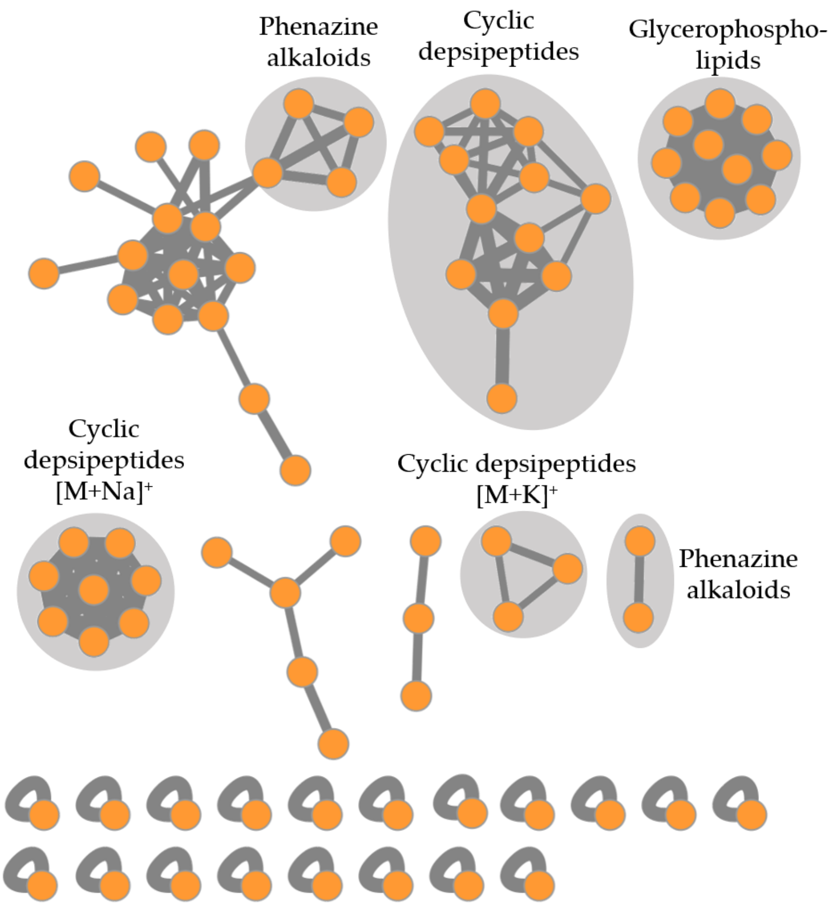

Figure S4. FBMN of *Micromonospora* sp. extract CKG20-GYM. The width of edges represents the cosine similarity between two nodes. The FBMN was generated with edges having cosine score above 0.8. See Table S6 for putatively annotated compounds.

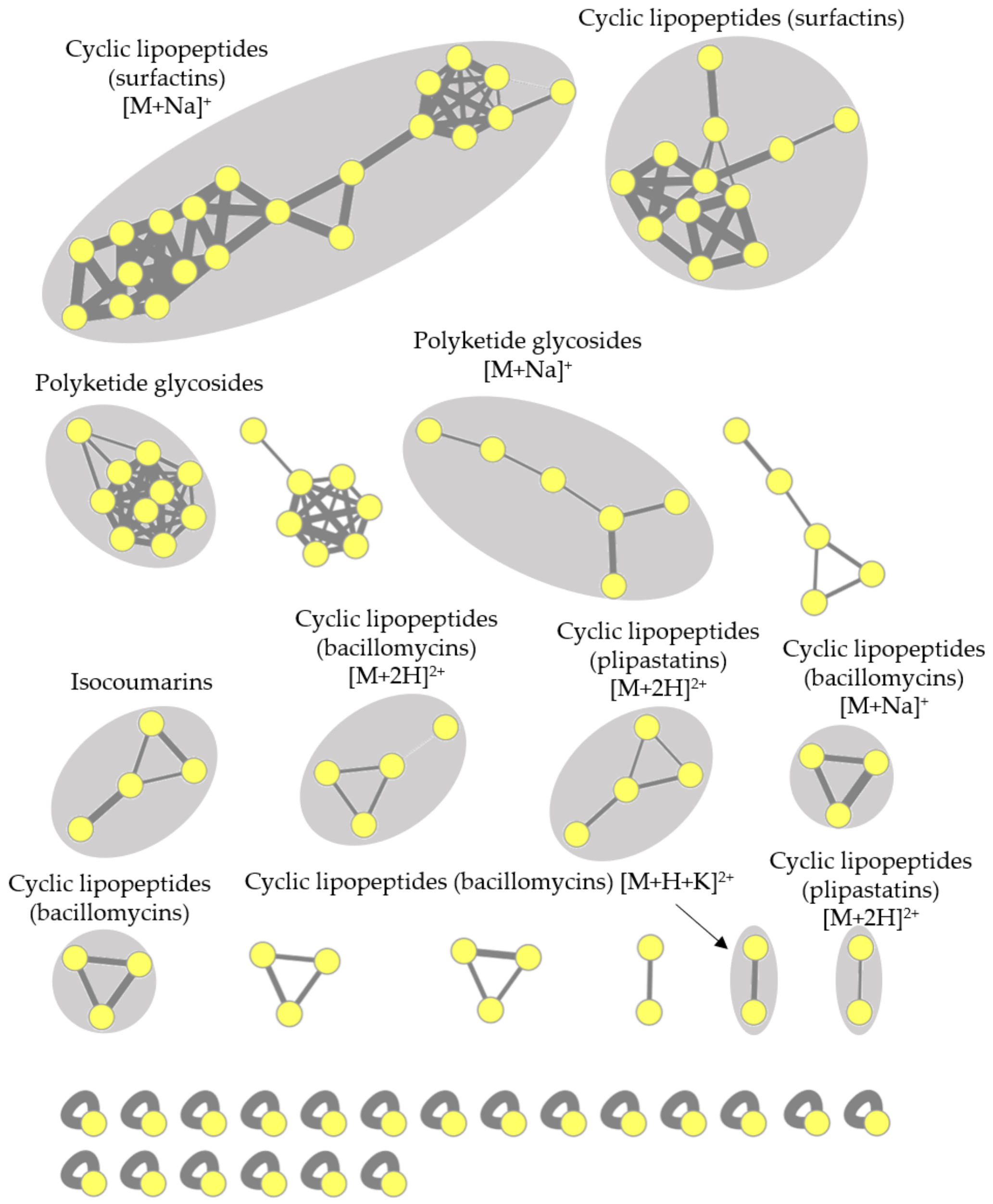

Figure S5. FBMN of *Bacillus* sp. extract CKG24-GYM. The width of edges represents the cosine similarity between two nodes. See Table S7 for putatively annotated compounds.

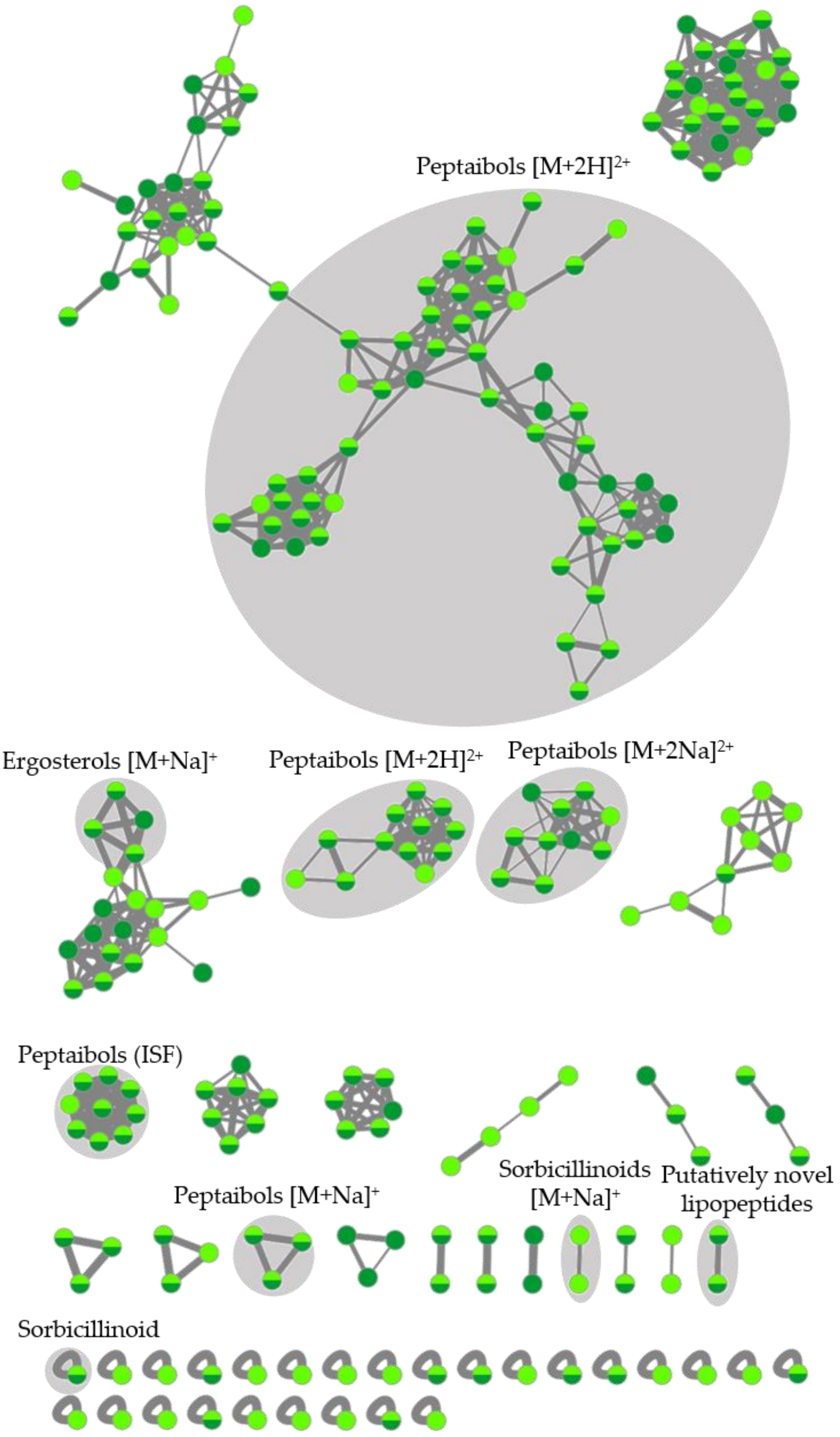

Figure S6. FBMN of *Trichoderma* sp. extracts CHG34-CAG and CHG34-PDA. The width of edges represents the cosine similarity between two nodes. Nodes are color-coded by the respective extracts: light green: CHG34-CAG, dark green: CHG34-PDA. ISF: abundant in source fragments of detected metabolites. See Table S8 for putatively annotated compounds.

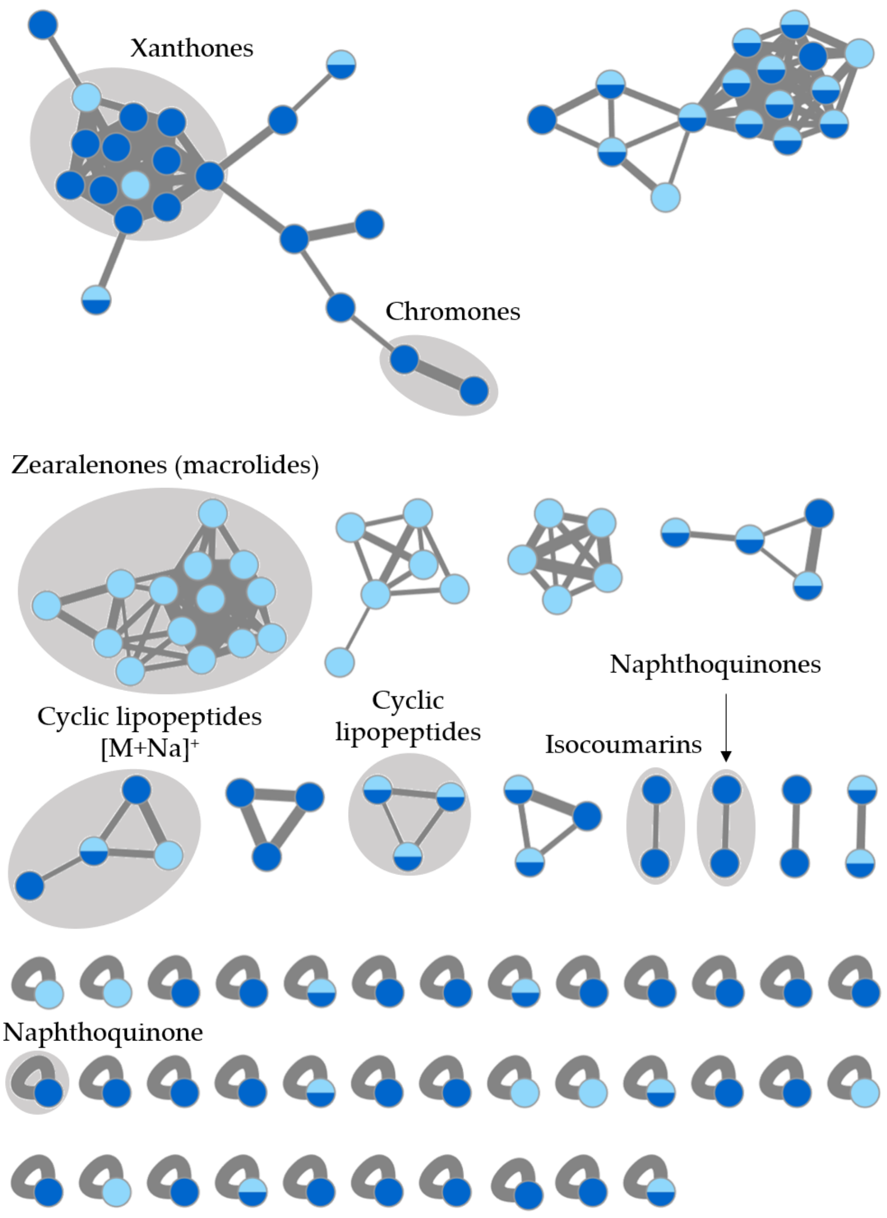

Figure S7. FBMN of *Fusarium* sp. extracts CHG38-CAG and CHG38-PDA. The width of edges represents the cosine similarity between two nodes. Nodes are color-coded by the respective extracts: light blue: CHG38-CAG, dark blue: CHG38-PDA. See Table S9 for putatively annotated compounds.

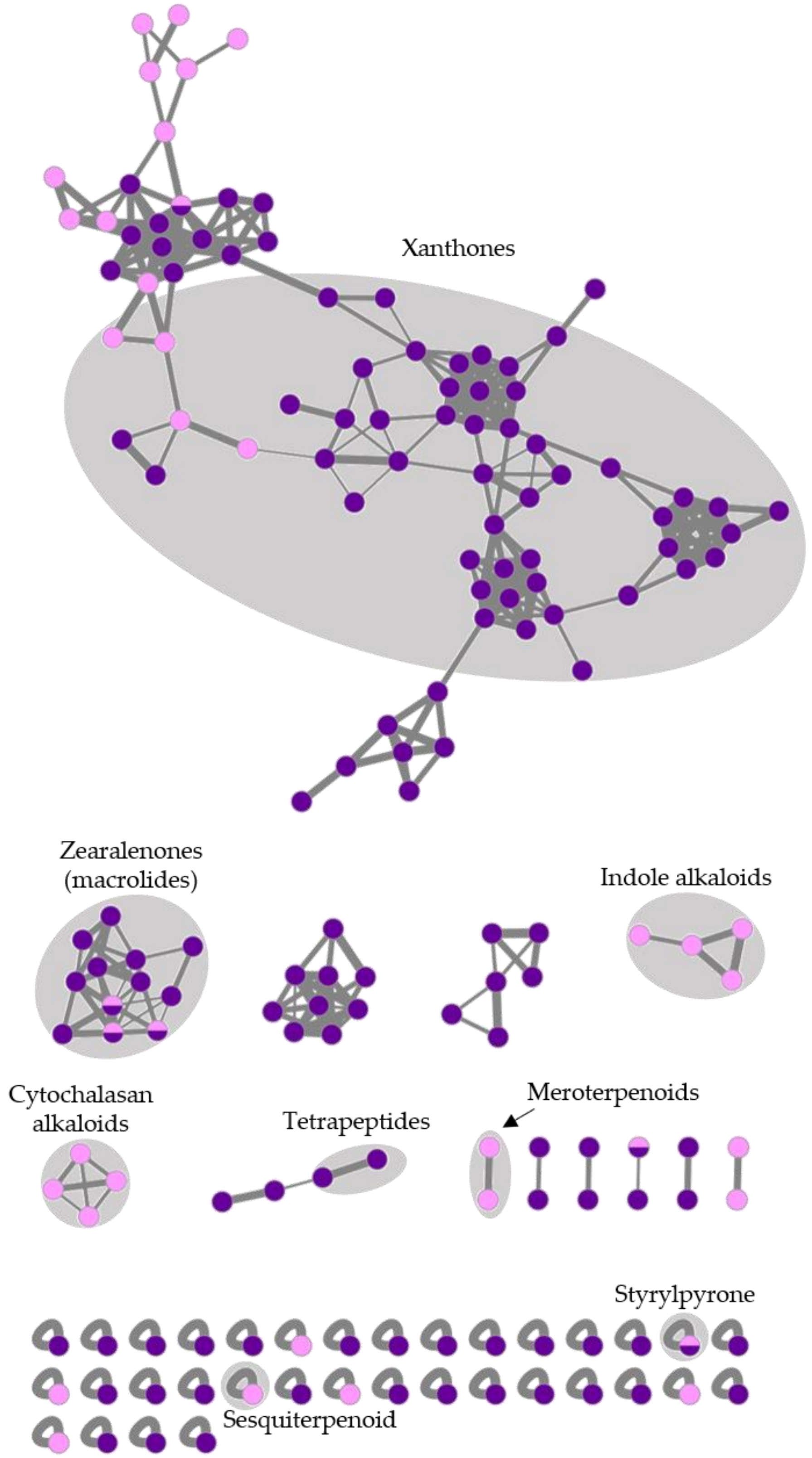

Figure S8. FBMN of *Penicillium* sp. extracts CKG23-CAG and CKG23-PDA. The width of edges represents the cosine similarity between two nodes. Nodes are color-coded by the respective extracts: light purple: CKG23-CAG, dark purple: CKG23-PDA. See Table S10 for putatively annotated compounds.

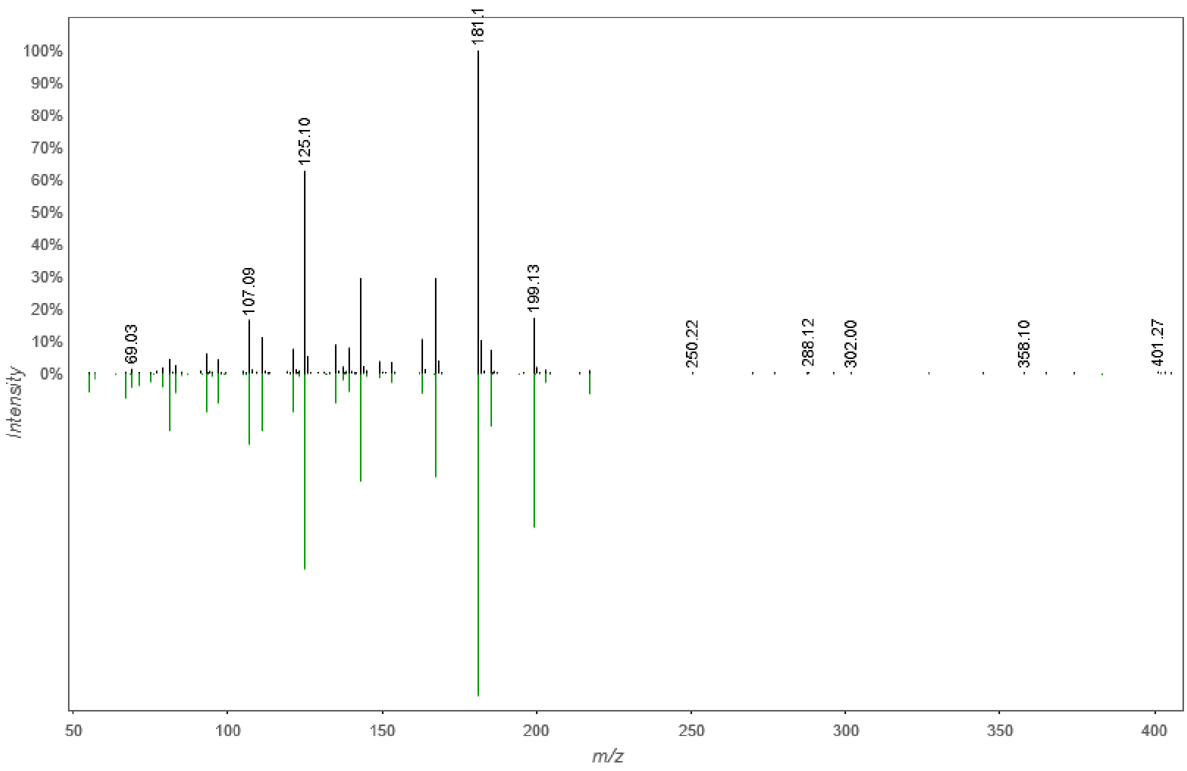

Figure S9. Experimental (black) and library (green) MS/MS spectra of bonactin (**14**), putatively identified in *Streptomyces* sp. extract CHG48-GYM. The spectral match was generated by the online platform GNPS [1].

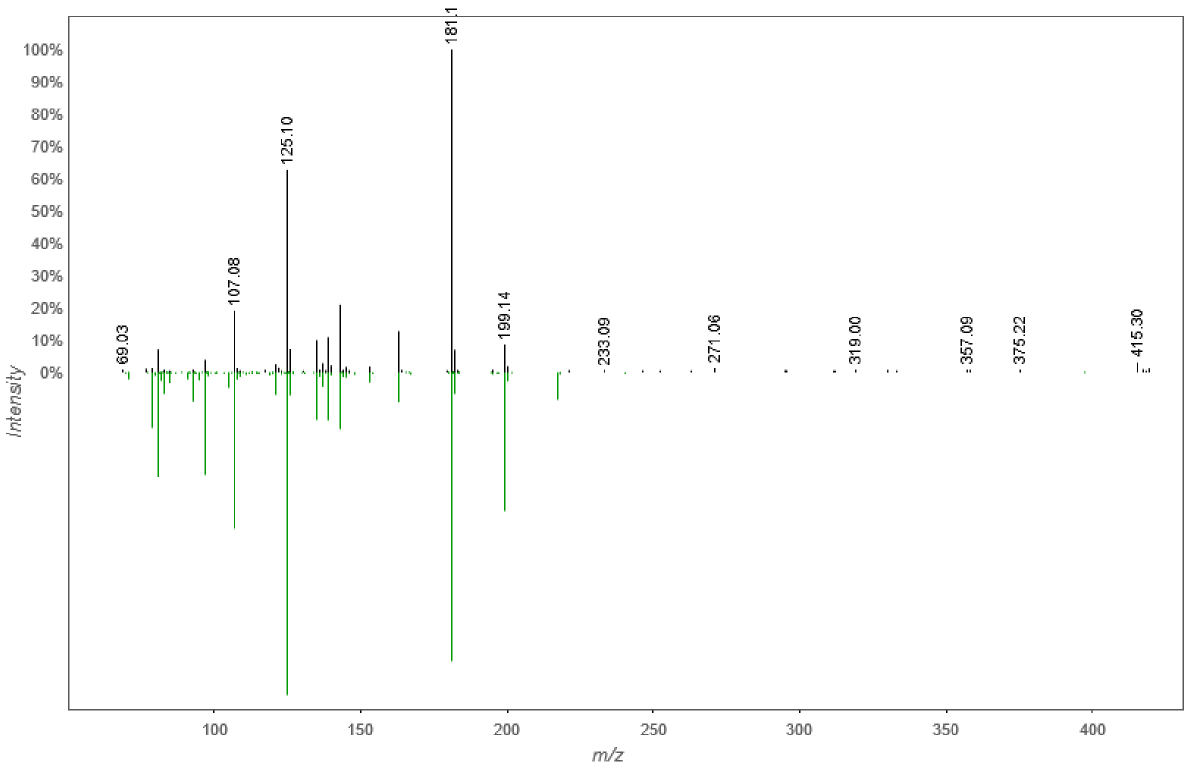

Figure S10. Experimental (black) and library (green) MS/MS spectra of homononactyl homononactate (**18**), putatively identified in *Streptomyces* sp. extract CHG48-GYM. The spectral match was generated by the online platform GNPS [1].

CHG34-PDA: MS/MS spectrum *m/z* 770.5386 $[M+H]^+$ (**103**)

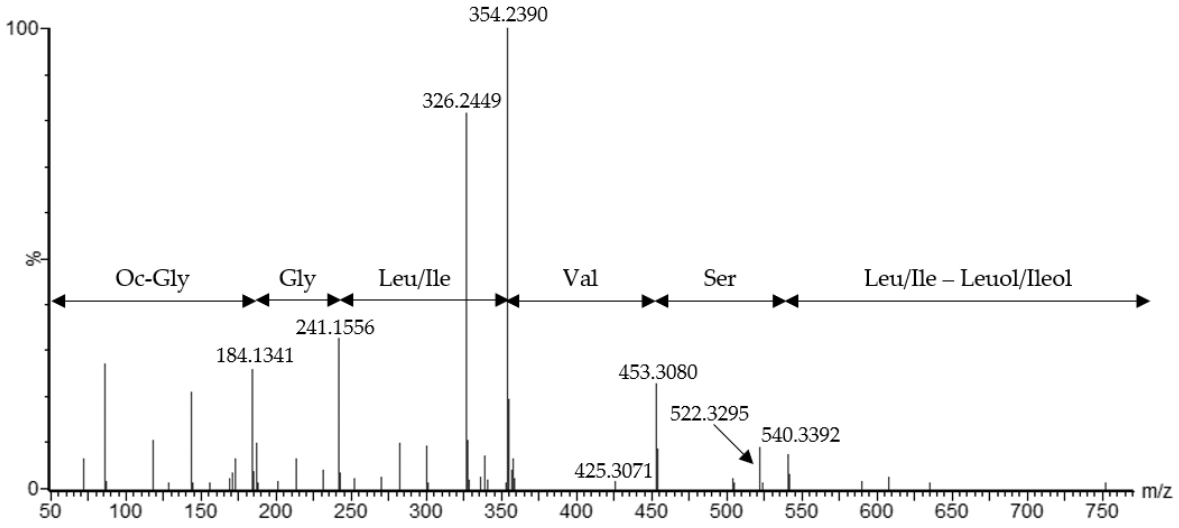

CHG34-PDA: MS/MS spectrum *m/z* 754.5424 $[M+H]^+$ (**105**)

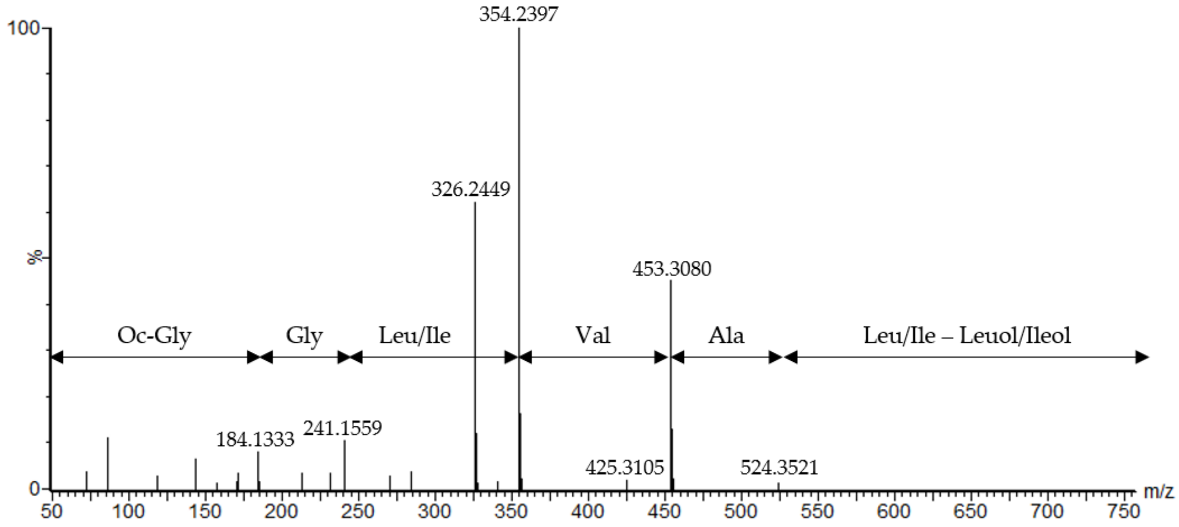

Figure S11. Annotated MS/MS spectra of putatively novel lipopeptides detected in *Trichoderma* sp. extracts CHG34-CAG and CHG34-PDA. The putative amino acid sequences were predicted based on the experimentally determined MS/MS fragmentation pattern of *m/z* 770.5386 $[M+H]^+$ (**103**; top) and *m/z* 754.5423 $[M+H]^+$ (**105**; bottom; here shown for extract CHG34-PDA). Ala: alanine, Gly: glycine, Leu/Ile: (iso)leucine, Leuol/Ileol: (iso)leucinol, Oc: octanoyl, Ser: serine, Val: valine.

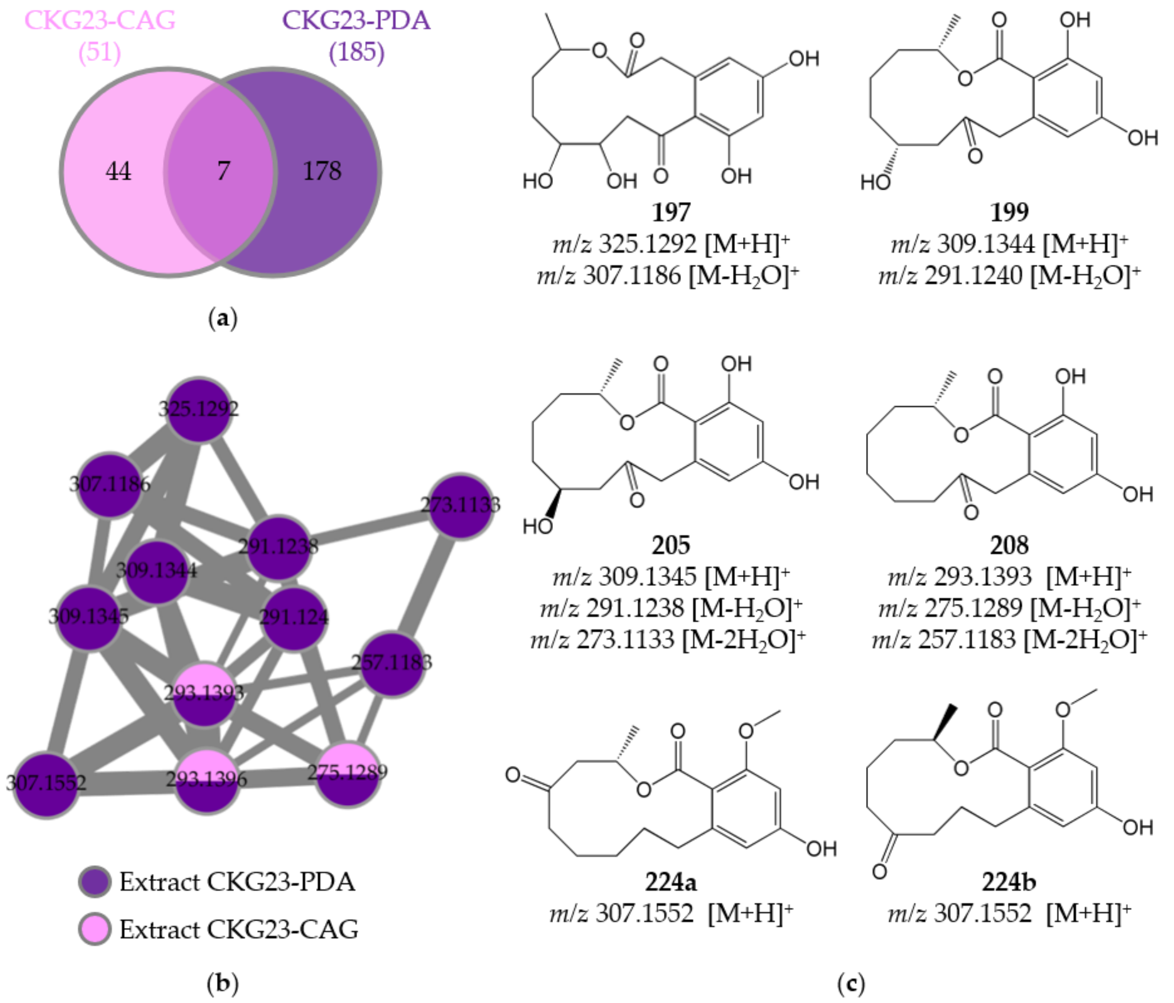

Figure S12. Comparative metabolome analyses of *Penicillium* sp. extracts CKG23-CAG and CKG23-PDA. (**a**) Venn diagram of shared and exclusive peaks detected in extract CKG23-CAG (light purple) and CKG23-PDA (dark purple). (**b**) Zearalenone cluster produced by *Penicillium* sp. isolate CKG23 (see Figure S8 for full FBMN). The width of edges represents the cosine similarity between two nodes. (**c**) Putatively annotated metabolites of the zearalenone cluster are displayed with their *m/z* values and observed adducts. Compound numbers are in accordance with Table S10.

Table S1. Taxonomic classification of microbial strains isolated from the gut of *C. intestinalis* sampled in Helgoland and Kiel Fjord. Strain codes are based on the respective sampling location and sampled tissue (CHG = *C. intestinalis* from Helgoland, gut; CKG = *C. intestinalis* from Kiel Fjord, gut). The three closest related strains are given according to the BLAST search [2]. RG = risk group (according to TRBA 460 and TRBA 466). Acc. no.: Accession number.

Strain code	Isolation medium	Acc. no.	Amplicon	Closest related species (BLAST)	Acc. no. closest related species	Lowest taxonomic classification (order)	RG
CHG1	MB	MW065489	16S	Uncultured *Vibrio* sp.<br>Uncultured *Vibrio* sp.<br>*Vibrio owensii*	MG554543.1<br>MG554505.1<br>CP025797.1	*Vibrio* sp. (Vibrionales)	1
CHG2	MB	MW065490	16S	*Ruegeria* sp.<br>*Ruegeria* sp.<br>*Ruegeria atlantica*	KY363633.1<br>KX833139.1<br>JN128252.1	*Ruegeria atlantica* (Rhodobacterales)	1
CHG3	MB	MW065491	16S	Bacterium b1cb16<br>*Shewanella* sp.<br>*Shewanella piezotolerans*	EF207071.1<br>MF975607.1<br>CP000472.1	*Shewanella* sp. (Alteromonadales)	1
CHG4	MB	MW065492	16S	*Citrobacter amalonaticus*<br>*Citrobacter farmeri*<br>*Citrobacter* sp.	MG238567.1<br>CP022695.1<br>LT556085.1	*Citrobacter* sp. (Enterobacterales)	2
CHG5	MB	MW065493	16S	*Shewanella woodyi*<br>*Shewanella woodyi*<br>*Shewanella woodyi*	NR_114412.1<br>NR_074846.1<br>CP000961.1	*Shewanella woodyi* (Alteromonadales)	1
CHG6	TSB	MW065494	16S	*Shewanella schlegeliana*<br>*Shewanella sairae*<br>*Shewanella schlegeliana*	MG388302.1<br>AB274769.1<br>AB274767.1	*Shewanella* sp. (Alteromonadales)	1
CHG7	TSB	MW065495	16S	*Shewanella putrefaciens*<br>*Shewanella hafniensis*<br>*Shewanella xiamenensis*	MG976650.1<br>MF612155.1<br>MF033476.1	*Shewanella* sp. (Alteromonadales)	2
CHG8	TSB	MW065496	16S	*Shewanella* sp.<br>*Shewanella* sp.<br>*Shewanella algae*	KJ748462.1<br>KJ748460.1<br>KX218308.1	*Shewanella* sp. (Alteromonadales)	2

Strain code	Isolation medium	Acc. no.	Amplicon	Closest related species (BLAST)	Acc. no. closest related species	Lowest taxonomic classification (order)	RG
CHG9	TSB	MW065497	16S	*Escherichia coli*<br>*Escherichia coli*<br>*Escherichia coli*	CP025753.1<br>CP025747.1<br>CP025739.1	*Escherichia coli* (Enterobacterales)	2
CHG10	CMN	MW065498	16S	*Vibrio alginolyticus*<br>*Vibrio alginolyticus*<br>*Vibrio alginolyticus*	KY229785.1<br>JN188414.1<br>KY684259.1	*Vibrio* sp. (Vibrionales)	2
CHG11	CMN	MW065499	16S	*Ruegeria* sp.<br>*Ruegeria* sp.<br>*Ruegeria atlantica*	KY363633.1<br>KX833139.1<br>HQ908680.1	*Ruegeria atlantica* (Rhodobacterales)	1
CHG12	CMN	MW065500	16S	*Shewanella kaireitica*<br>Uncultured bacterium clone SanDiego_a2585<br>Uncultured bacterium clone SanDiego_a2547	KX078089.1<br>KF799676.1<br>KF799675.1	*Shewanella* sp. (Alteromonadales)	1
CHG16	WSP	MW065501	16S	*Vibrio* sp.<br>*Vibrio* sp.<br>*Vibrio owensii*	MK533523.1<br>MK533517.1<br>CP033138.1	*Vibrio* sp. (Vibrionales)	1
CHG19	WSP	MW064137	ITS	Fungal sp. isolate whc1<br>*Arthrinium arundinis*<br>*Arthrinium sacchari*	MH465392.1<br>KX778674.1<br>KX778672.1	*Arthrinium* sp. (Xylariales)	1
CHG20	WSP	MW065502	16S	*Citrobacter freundii*<br>*Citrobacter freundii complex*<br>*Citrobacter freundii*	CP027849.1<br>CP026231.1<br>CP011657.1	*Citrobacter freundii* (Enterobacterales)	2
CHG21	WSP	MW065503	16S	*Citrobacter braakii*<br>*Citrobacter* sp.<br>*Citrobacter braakii*	MH368419.1<br>MH368123.1<br>CP022049.2	*Citrobacter braakii* (Enterobacterales)	2
CHG22	WSP	MW064138	ITS	Uncultured *Penicillium* clone WPW-OTU-32<br>*Penicillium hoeksii*<br>*Penicillium hoeksii*	KT581734.1<br>NR_137913.1<br>KY305048.1	*Penicillium hoeksii* (Eurotiales)	1

Strain code	Isolation medium	Acc. no.	Amplicon	Closest related species (BLAST)	Acc. no. closest related species	Lowest taxonomic classification (order)	RG
CHG24	WSP	MW064139	ITS	*Aspergillus niger*<br>Fungal sp. SNB-LAP1-7-61<br>*Aspergillus niger*	MG647867.1<br>KU977335.1<br>MH050790.1	*Aspergillus* sp. (Eurotiales)	2
CHG25	WSP	MW064140	ITS	*Penicillium expansum*<br>*Penicillium* sp.<br>*Penicillium ulaiense*	MF303721.1<br>KP403971.1<br>LN871568.1	*Penicillium* sp. (Eurotiales)	1
CHG26	WSP	MW064141	ITS	Uncultured *Galactomyces* clone P71B<br>*Geotrichum candidum*<br>*Geotrichum candidum*	MG193553.1<br>MH680587.1<br>MF782775.1	*Galactomyces candidum* (Saccharomycetales)	1
CHG28	WSP	MW064142	ITS	*Trichoderma harzianum*<br>*Trichoderma* sp.<br>*Trichoderma harzianum*	MF871539.1<br>MH285106.1<br>KY750434.1	*Trichoderma* sp. (Hypocreales)	1
CHG29	PDA	MW064143	ITS	*Phoma* sp.<br>Uncultured *Didymella* clone 191_K9ov<br>*Stagonosporopsis cucurbitacearum*	KF525844.1<br>KY430454.1<br>KU168143.1	*Phoma* sp. (Pleosporales)	1
CHG32	TSB	MW065504	16S	*Vibrio rumoiensis*<br>*Vibrio* sp.<br>*Vibrio owensii*	AP018685.1<br>MG262453.1<br>MH368433.1	*Vibrio* sp. (Vibrionales)	1
CHG34	WSP	MW064144	ITS	*Trichoderma* sp.<br>*Trichoderma harzianum*<br>*Trichoderma harzianum*	MH285106.1<br>MF871539.1<br>KY750434.1	*Trichoderma* sp. (Hypocreales)	1
CHG35	PDA	MW064145	ITS	*Penicillium* sp.<br>*Penicillium antarcticum*<br>*Penicillium atrovenetum*	KY401117.1<br>KP016829.1<br>KF679753.1	*Penicillium* sp. (Eurotiales)	1
CHG38	WSP	MW064146	ITS	*Fusarium graminearum*<br>*Fusarium graminearum*<br>*Fusarium graminearum*	MK079896.1<br>MK079895.1<br>MK079894.1	*Fusarium* sp. (Hypocreales)	1
CHG39	WSP	MW065505	16S	*Bacillus subtilis*<br>*Bacillus subtilis*<br>*Bacillus velezensis*	MG977677.1<br>MG976620.1<br>MG970354.1	*Bacillus* sp. (Bacillales)	1

Strain code	Isolation medium	Acc. no.	Amplicon	Closest related species (BLAST)	Acc. no. closest related species	Lowest taxonomic classification (order)	RG
CHG40	TSB	MW065506	16S	*Streptomyces* sp.<br>*Streptomyces parvus*<br>*Streptomyces lavendulae*	KY613504.1<br>KY213676.1<br>KY213666.1	*Streptomyces* sp. (Streptomycetales)	1
CHG41	TSB	MW065507	16S	*Bacillus licheniformis*<br>Uncultured bacterium clone OTU3<br>*Bacillus licheniformis*	KY886241.1<br>KP975259.1<br>KC522129.1	*Bacillus licheniformis* (Bacillales)	1
CHG42	TSB	MW065508	16S	*Shewanella algae*<br>*Shewanella algae*<br>*Shewanella haliotis*	NR_114236.1<br>NR_117771.1<br>NR_117770.1	*Shewanella* sp. (Alteromonadales)	2
CHG43	MB	MW065509	16S	*Vibrio splendidus*<br>*Vibrio* sp.<br>*Vibrio anguillarum*	MH010050.1<br>MG788349.1<br>CP023433.1	*Vibrio* sp. (Vibrionales)	2
CHG44	MB	MW064147	ITS	*Aspergillus oryzae*<br>*Aspergillus oryzae*<br>*Aspergillus flavus*	MH746006.1<br>MH625703.1<br>MH578599.1	*Aspergillus* sp. (Eurotiales)	2
CHG47	WSP	MW064148	ITS	*Arthrinium* sp.<br>*Arthrinium* sp.<br>*Arthrinium arundinis*	MH059547.1<br>MH059539.1<br>MK256947.1	*Arthrinium* sp. (Xylariales)	1
CHG48	WSP	MW065510	16S	*Streptomyces* sp.<br>*Streptomyces* sp.<br>*Streptomyces pratensis*	MG637270.1<br>MG637268.1<br>MK484235.1	*Streptomyces* sp. (Streptomycetales)	1
CHG49	PDA	MW064175	18S	Uncultured fungus clone nco40d10c1<br>Uncultured fungus clone nco40a09c1<br>*Pyrenophora tritici-repentis*	KC670836.1<br>KC670799.1<br>U42486.1	Pleosporaceae (Pleosporales)	1
CHG52	WSP	MW064149	ITS	*Penicillium cosmopolitanum*<br>*Penicillium cosmopolitanum*<br>*Penicillium cosmopolitanum*	MH864385.1<br>MH864384.1<br>MH864377.1	*Penicillium* sp. (Eurotiales)	1
CHG53	PDA	MW064150	ITS	*Trichoderma* sp.<br>*Trichoderma* sp.<br>*Trichoderma koningii*	MH794211.1<br>MH284929.1<br>KX343123.1	*Trichoderma* sp. (Hypocreales)	1

Strain code	Isolation medium	Acc. no.	Amplicon	Closest related species (BLAST)	Acc. no. closest related species	Lowest taxonomic classification (order)	RG
CHG56	TSB	MW064151	ITS	*Aspergillus nidulans*<br>Fungal sp. isolate *Aspergillus quadrilineatus*<br>*Aspergillus nidulans*	MG459155.1<br>MH041155.1<br>MG991576.1	*Aspergillus* sp. (Eurotiales)	1
CHG59	PDA	MW064152	ITS	*Tamaricicola* sp.<br>*Tamaricicola* sp.<br>*Comoclathris spartii*	MG977427.1<br>MG977425.1<br>KU714703.1	*Tamaricicola* sp. (Pleosporales)	1
CHG60	TSB	MW064153	ITS	*Peroneutypa* sp.<br>*Eutypella scoparia*<br>*Eutypella* sp.	MF359647.1<br>EU436688.1<br>JQ922161.1	*Peroneutypa* sp. (Xylariales)	1
CHG61	TSB	MW065511	16S	Uncultured bacterium clone FRA_187_C04_2008-07-09<br>Uncultured *Vibrio* sp.<br>*Vibrio anguillarum*	FN434816.1<br>MG554532.1<br>CP022468.1	*Vibrio* sp. (Vibrionales)	2
CHG64	TSB	MW065512	16S	*Streptomyces microflavus*<br>*Streptomyces* sp.<br>*Streptomyces* sp.	MG855947.1<br>MH251131.1<br>MH250821.1	*Streptomyces* sp. (Streptomycetales)	1
CKG1	MB	MW065513	16S	*Pseudomonas* sp.<br>Uncultured marine bacterium isolate TGGE gel band 22TGGE1<br>*Pseudomonas anguilliseptica*	KX621130.1<br>KJ814609.1<br>DQ005209.1	*Pseudomonas* sp. (Pseudomonadales)	1
CKG2	MB	MW065514	16S	*Rhodococcus* sp.<br>*Rhodococcus antrifimi*<br>*Rhodococcus antrifimi*	MG515722.1<br>LN867321.1<br>NR_145614.1	*Rhodococcus* sp. (Corynebacteriales)	2
CKG3	MB	MW065515	16S	*Shewanella* sp.<br>*Shewanella* sp.<br>*Shewanella aestuarii*	FR821223.1<br>FR744880.1<br>KX271676.1	*Shewanella* sp. (Alteromonadales)	1
CKG4	MB	MW065516	16S	*Vibrio* sp.<br>*Vibrio anguillarum*<br>*Vibrio anguillarum*	MG788349.1<br>CP023433.1<br>CP023293.1	*Vibrio* sp. (Vibrionales)	2

Strain code	Isolation medium	Acc. no.	Amplicon	Closest related species (BLAST)	Acc. no. closest related species	Lowest taxonomic classification (order)	RG
CKG5	MB	MW065517	16S	*Nocardiopsis alba*<br>*Nocardiopsis alba*<br>*Nocardiopsis alba*	MH843138.1<br>MH843137.1<br>MH843136.1	*Nocardiopsis* sp. (Streptosporangiales)	1
CKG6	MB	MW065518	16S	*Shewanella aestuarii*<br>*Shewanella aestuarii*<br>*Shewanella aestuarii*	KX271676.1<br>KX271675.1<br>KX271674.1	*Shewanella aestuarii* (Alteromonadales)	1
CKG7	MB	MW065519	16S	*Vibrio owensii*<br>*Vibrio owensii*<br>*Vibrio owensii*	LC369696.1<br>MG896198.1<br>MG896189.1	*Vibrio* sp. (Vibrionales)	1
CKG8	MB	MW065520	16S	*Vibrio* sp.<br>*Vibrio anguillarum*<br>*Vibrio anguillarum*	MG788349.1<br>CP023433.1<br>CP023293.1	*Vibrio* sp. (Vibrionales)	2
CKG9	CMN	MW065521	16S	*Vibrio* sp.<br>*Vibrio anguillarum*<br>*Vibrio anguillarum*	MG788349.1<br>CP023433.1<br>CP023293.1	*Vibrio* sp. (Vibrionales)	2
CKG10	CMN	MW065522	16S	Uncultured *Vibrio* sp. clone HH101354<br>Uncultured *Vibrio* sp. clone HH101351<br>*Vibrio anguillarum*	MG554532.1<br>MG554529.1<br>CP022468.1	*Vibrio* sp. (Vibrionales)	2
CKG11	CMN	MW065523	16S	*Shewanella aestuarii*<br>*Shewanella aestuarii*<br>*Shewanella aestuarii*	KX271676.1<br>KX271675.1<br>KX271674.1	*Shewanella aestuarii* (Alteromonadales)	1
CKG12	TSB	MW065524	16S	*Vibrio owensii*<br>*Vibrio campbellii*<br>*Vibrio* sp.	MG896198.1<br>CP026321.1<br>KY655411.1	*Vibrio* sp. (Vibrionales)	1
CKG13	TSB	MW065525	16S	Uncultured marine bacterium isolate TGGE gel band 22TGGE1<br>*Pseudomonas* sp.<br>*Pseudomonas peli*	KJ814609.1<br>JQ012964.1<br>MG687270.1	*Pseudomonas* sp. (Pseudomonadales)	1
CKG14	TSB	MW065526	16S	*Shewanella* sp.<br>*Shewanella* sp.<br>*Shewanella aestuarii*	FR821223.1<br>FR744880.1<br>KX271676.1	*Shewanella* sp. (Alteromonadales)	1

Strain code	Isolation medium	Acc. no.	Amplicon	Closest related species (BLAST)	Acc. no. closest related species	Lowest taxonomic classification (order)	RG
CKG15	TSB	MW065527	16S	*Shewanella* sp.<br>*Shewanella* sp.<br>*Shewanella colwelliana*	MF045123.1<br>KU647920.1<br>KC534404.1	*Shewanella colwelliana* (Alteromonadales)	1
CKG16	TSB	MW064154	ITS	*Eutypa lata*<br>*Amphisphaeria umbrina*<br>Uncultured *Eutypella* clone G17312	MF359647.1<br>KY962999.1<br>JQ922161.1	*Eutypa lata* (Xylariales)	1
CKG19	TSB	MW065528	16S	*Bacillus pumilus*<br>*Bacillus pumilus*<br>*Bacillus zhangzhouensis*	CP027034.1<br>KY623354.1<br>MG937731.1	*Bacillus* sp. (Bacillales)	2
CKG20	TSB	MW065529	16S	*Micromonospora* sp.<br>*Micromonospora* sp.<br>*Micromonospora aurantiaca*	EU437811.1<br>LC383890.1<br>MH333275.1	*Micromonospora* sp. (Micromonosporales)	1
CKG21	CMN	MW065530	16S	*Bacillus hwajinpoensis*<br>*Bacillus hwajinpoensis*<br>*Bacillus hwajinpoensis*	MG651497.1<br>MG651463.1<br>MG651074.1	*Bacillus hwajinpoensis* (Bacillales)	1
CKG22	PDA	MW064155	ITS	*Penicillium antarcticum*<br>*Penicillium* sp.<br>*Penicillium* sp.	MH828228.1<br>KY401122.1<br>KY401117.1	*Penicillium* sp. (Eurotiales)	1
CKG23	WSP	MW064156	ITS	*Penicillium antarcticum*<br>*Penicillium* sp.<br>*Penicillium* sp.	MH828228.1<br>KY401122.1<br>KY401117.1	*Penicillium* sp. (Eurotiales)	1
CKG24	WSP	MW065531	16S	*Bacillus subtilis*<br>*Bacillus amyloliquefaciens*<br>*Bacillus subtilis*	KC428745.1<br>JX517210.1<br>GQ280056.1	*Bacillus* sp. (Bacillales)	1
CKG25	WSP	MW064157	ITS	*Geotrichum candidum*<br>*Galactomyces candidum*<br>*Geotrichum candidum*	KF713521.1<br>KF298070.1<br>KY009607.1	*Galactomyces candidum* (Saccharomycetales)	1
CKG27	WSP	MW065532	16S	*Bacillus muralis*<br>*Bacillus* sp.<br>*[Brevibacterium] frigoritolerans*	MF506797.1<br>MG062899.2<br>MF467864.1	*Bacillus* sp. (Bacillales)	2

Strain code	Isolation medium	Acc. no.	Amplicon	Closest related species (BLAST)	Acc. no. closest related species	Lowest taxonomic classification (order)	RG
CKG29	WSP	MW065533	16S	*Bacillus amyloliquefaciens*<br>*Bacillus amyloliquefaciens*<br>*Bacillus subtilis*	MG136848.1<br>MG136846.1<br>MG977677.1	*Bacillus* sp. (Bacillales)	1
CKG30	WSP	MW065534	16S	*Bacillus pumilus*<br>*Bacillus pumilus*<br>*Bacillus zhangzhouensis*	CP027034.1<br>KY623354.1<br>MG937731.1	*Bacillus* sp. (Bacillales)	2
CKG31	PDA	MW065535	16S	*Vibrio* sp.<br>*Shewanella* sp.<br>*Shewanella kaireitica*	LC416561.1<br>MH333258.1<br>KX078089.1	*Shewanella kaireitica* (Alteromonadales)	1
CKG32	WSP	MW064158	ITS	*Fusarium graminearum*<br>*Fusarium graminearum*<br>*Fusarium graminearum*	MF800908.1<br>KY466827.1<br>KY466825.1	*Fusarium* sp. (Hypocreales)	1
CKG33	WSP	MW064159	ITS	*Mucor hiemalis*<br>*Mucor hiemalis*<br>*Mucor hiemalis*	HQ845045.1<br>HM037964.1<br>HM037963.1	*Mucor hiemalis* (Mucorales)	1
CKG36	TSB	MW065536	16S	*Bacillus* sp.<br>*Bacillus cereus*<br>*Bacillus cereus*	CP020437.2<br>MG977683.1<br>MG966498.1	*Bacillus* sp. (Bacillales)	2
CKG37	CMN	MW064160	ITS	*Sarocladium strictum*<br>*Sarocladium strictum*<br>*Sarocladium strictum*	MH880255.1<br>LC314675.1<br>MF663649.1	*Sarocladium strictum* (Hypocreales)	1
CKG38	CMB	MW065537	16S	*Pseudomonas* sp.<br>*Pseudomonas* sp.<br>*Pseudomonas anguilliseptica*	KT710819.1<br>KT710818.1<br>JX177684.1	*Pseudomonas anguilliseptica* (Pseudomonadales)	1
CKG39	TSB	MW065538	16S	*Bacillus licheniformis*<br>*Bacillus licheniformis*<br>*Bacillus licheniformis*	MG980062.1<br>MG280960.1<br>MG189544.1	*Bacillus* sp. (Bacillales)	1
CKG40	WSP	MW065539	16S	Uncultured *Klebsiella* sp. clone JXS1-28<br>*Klebsiella* sp.<br>*Raoultella ornithinolytica*	JN873189.1<br>KM873628.1<br>CP010557.1	*Klebsiella* sp. (Enterobacterales)	2

Strain code	Isolation medium	Acc. no.	Amplicon	Closest related species (BLAST)	Acc. no. closest related species	Lowest taxonomic classification (order)	RG
CKG42	WSP	MW064161	ITS	*Elaphocordyceps* sp.<br>*Elaphocordyceps* sp.<br>*Tolypocladium* sp.	KC237381.1<br>KC237380.1<br>KX034386.1	*Elaphocordyceps* sp. (Hypocreales)	1
CKG43	WSP	MW065540	16S	*Bacillus subtilis*<br>*Bacillus siamensis*<br>*Bacillus siamensis*	MG928427.1<br>KY962351.1<br>KY962340.1	*Bacillus* sp. (Bacillales)	1
CKG44	WSP	MW064162	ITS	*Neonectria coccinea*<br>Uncultured *Neonectria* clone AEW3_110<br>*Neonectria coccinea*	KJ022022.1<br>KF823598.1<br>KC660506.1	*Neonectria coccinea* (Hypocreales)	1
CKG45	WSP	MW064163	ITS	*Purpureocillium lilacinum*<br>*Purpureocillium lilacinum*<br>*Purpureocillium lilacinum*	KY007618.1<br>MH865347.1<br>MH865301.1	*Purpureocillium lilacinum* (Hypocreales)	2
CKG46	TSB	MW065541	16S	*Nocardiopsis* sp.<br>*Nocardiopsis prasina*<br>*Nocardiopsis prasina*	MK045298.1<br>MF594115.1<br>MF170851.1	*Nocardiopsis prasina* (Streptosporangiales)	1
CKG47	CMN	MW065542	16S	*Vibrio* sp.<br>*Vibrio anguillarum*<br>*Vibrio anguillarum*	MG788349.1<br>CP023433.1<br>CP023293.1	*Vibrio* sp. (Vibrionales)	2
CKG49	WSP	MW064164	ITS	*Mucor circinelloides*<br>*Mucor circinelloides*<br>*Mucor circinelloides*	MH911362.1<br>KC329629.1<br>JX241658.1	*Mucor circinelloides* (Mucorales)	1
CKG50	CMN	MW065543	16S	*Sporosarcina* sp.<br>*Sporosarcina* sp.<br>*Sporosarcina aquimarina*	KX108967.1<br>KT368976.1<br>KF800793.1	*Sporosarcina* sp. (Bacillales)	1
CKG51	CMN	MW064165	ITS	*Penicillium polonicum*<br>*Penicillium polonicum*<br>*Penicillium* sp.	KY978579.1<br>KY993979.1<br>KY092668.1	*Penicillium* sp. (Eurotiales)	1
CKG52	CMN	MW065544	16S	*Vibrio* sp.<br>*Vibrio anguillarum*<br>*Vibrio anguillarum*	MG788349.1<br>CP023433.1<br>CP023293.1	*Vibrio* sp. (Vibrionales)	2

Strain code	Isolation medium	Acc. no.	Amplicon	Closest related species (BLAST)	Acc. no. closest related species	Lowest taxonomic classification (order)	RG
CKG53	CMN	MW065545	16S	*Streptomyces* sp.<br>*Streptomyces* sp.<br>*Streptomyces lividans*	MK134635.1<br>MK134629.1<br>MG856044.1	*Streptomyces* sp. (Streptomycetales)	2
CKG54	CMB	MW064176	18S	*Cordyceps farinosa*<br>Fungal sp. J271<br>*Isaria farinosa*	MH857775.1<br>KC242715.1<br>KC242708.1	*Cordyceps farinosa* (Hypocreales)	1
CKG55	MB	MW065546	16S	*Nocardiopsis alba*<br>*Nocardiopsis alba*<br>*Nocardiopsis alba*	MH333283.1<br>MF321814.1<br>MF321809.1	*Nocardiopsis alba* (Streptosporangiales)	1
CKG57	CMB	MW064166	ITS	*Aaosphaeria arxii*<br>*Arthopyrenia* sp.<br>*Massarina igniaria*	MH861193.1<br>KU747910.1<br>KR534712.1	*Arthopyrenia* sp. (Pleosporales)	1
CKG58	CMB	MW065547	16S	*Nocardiopsis* sp.<br>*Nocardiopsis prasina*<br>*Nocardiopsis prasina*	MK045298.1<br>MF594115.1<br>MF170851.1	*Nocardiopsis prasina* (Streptosporangiales)	1
CKG60	MB	MW065548	16S	*Enterobacter* sp.<br>*Citrobacter gillenii*<br>*Citrobacter gillenii*	MF429589.1<br>MH392488.1<br>MG757538.1	*Citrobacter* sp. (Enterobacterales)	2
CKG62	PDA	MW064167	ITS	*Trichoderma* sp.<br>*Trichoderma lixii*<br>*Trichoderma harzianum*	MK290992.1<br>MK288146.1<br>MK209008.1	*Trichoderma* sp. (Hypocreales)	1
CKG63	WSP	MW064168	ITS	*Penicillium psychrosexualis*<br>*Penicillium psychrosexualis*<br>*Penicillium psychrosexualis*	MH864839.1<br>MH864838.1<br>MH864787.1	*Penicillium* sp. (Eurotiales)	1
CKG64	CMB	MW064169	ITS	*Penicillium polonicum*<br>*Penicillium polonicum*<br>*Penicillium polonicum*	MK271277.1<br>MK267441.1<br>MK077720.1	*Penicillium* sp. (Eurotiales)	1
CKG66	CMB	MW064170	ITS	Uncultured fungus clone ZB042802405(86)<br>Fungal sp. strain PS14<br>*Acrostalagmus luteoalbus*	MF962944.1<br>MH456880.1<br>KT824244.1	*Acrostalagmus luteoalbus* (Hypocreales)	1

Strain code	Isolation medium	Acc. no.	Amplicon	Closest related species (BLAST)	Acc. no. closest related species	Lowest taxonomic classification (order)	RG
CKG67_I	CMB	MW064171	ITS	*Purpureocillium lilacinum*<br>*Purpureocillium lilacinum*<br>*Purpureocillium lilacinum*	LC413751.1<br>MF996819.1<br>KF706346.1	*Purpureocillium lilacinum* (Hypocreales)	2
CKG67_II	WSP	MW065549	16S	*Nocardiopsis alba*<br>*Nocardiopsis alba*<br>*Nocardiopsis alba*	MH333283.1<br>MH071379.1<br>MF321814.1	*Nocardiopsis alba* (Streptosporangiales)	1
CKG68	WSP	MW064172	ITS	*Purpureocillium lilacinum*<br>*Purpureocillium lilacinum*<br>*Purpureocillium lilacinum*	MH865347.1<br>MH865301.1<br>MH865154.1	*Purpureocillium lilacinum* (Hypocreales)	2
CKG70	TSB	MW064173	ITS	*Plectosphaerella cucumerina*<br>Fungal sp. strain S255T<br>Fungal sp. strain S255S	MH791266.1<br>KU839553.1<br>KU839552.1	*Plectosphaerella cucumerina* (Glomerellales)	1
CKG71	TSB	MW064174	ITS	*Sarocladium strictum*<br>Fungal sp. strain S254T<br>Fungal sp. strain S254S	KY465763.1<br>KU839539.1<br>KU839538.1	*Sarocladium strictum* (Hypocreales)	1

Table S2. Antimicrobial and anticancer activities (% inhibition at a test concentration of 100 µg/mL) of microbial crude extracts. Inhibition values are given as average values of the two biological and two technical replicates. Some bacterial isolates were only cultivated on MB medium, since they did not grow on GYM medium. Extracts selected by the bioactivity selection criterion (see Section 2.3.) are highlighted in blue. MRSA: Methicillin-resistant *Staphylococcus aureus*, Efm: *Enterococcus faecium*, Ab: *Acinetobacter baumannii*, Ec: *Escherichia coli*, Kp: *Klebsiella pneumoniae*, Psa: *Pseudomonas aeruginosa*, Ca: *Candida albicans*, Cn: *Cryptococcus neoformans*, A375: Malignant melanoma, A549: Lung carcinoma, HCT116: Colon cancer, MB231: Breast cancer; "-": Inhibition ≤20%; in bold: Inhibition values ≥80%; **AC**: extract was selected based on high **anticancer** activity (inhibition ≥80%); **AM**: extract was selected based on high **antimicrobial** activity (inhibition ≥80%).

Strain	Identification	Medium	MRSA	Efm	Ab	Ec	Kp	Psa	Ca	Cn	A375	A549	HCT116	MB231	Selected?
CHG2	*Ruegeria atlantica*	MB	73	-	-	-	-	-	-	-	29	-	-	36	
CHG3	*Shewanella* sp.	MB	**92**	52	-	-	-	-	-	-	-	-	-	-	
CHG5	*Shewanella woodyi*	MB	**95**	78	-	-	-	-	-	-	-	-	-	-	
CHG6	*Shewanella* sp.	GYM	**100**	**92**	-	-	-	-	-	-	-	-	-	-	
		MB	**95**	**80**	-	40	-	-	-	-	-	-	-	-	
CHG12	*Shewanella* sp.	MB	**88**	**100**	-	-	-	-	-	-	-	-	-	26	
CHG16	*Vibrio* sp.	MB	**98**	**100**	-	-	-	-	-	-	-	-	-	-	
CHG19	*Arthrinium* sp.	CAG	-	-	-	-	-	65	-	-	71	78	75	78	
		PDA	-	-	-	-	-	-	-	-	26	53	25	44	
CHG22	*Penicillium hoeksii*	CAG	**85**	-	-	-	-	-	-	-	25	31	25	29	
		PDA	**84**	-	-	-	-	-	-	-	63	48	59	48	
CHG25	*Penicillium* sp.	CAG	**99**	**100**	**98**	**100**	**100**	**100**	-	-	**98**	**93**	**98**	**92**	Yes (AC)
		PDA	**98**	**84**	**97**	**100**	**100**	**99**	-	-	**99**	**98**	**99**	**95**	Yes (AC)
CHG26	*Galactomyces candidum*	CAG	**96**	33	-	-	-	-	-	-	-	-	-	-	
		PDA	54	-	-	-	-	-	-	-	-	-	-	-	
CHG29	*Phoma* sp.	CAG	-	-	-	-	-	-	-	-	-	-	-	-	
		PDA	-	-	-	-	-	-	-	-	-	-	-	-	
CHG32	*Vibrio* sp.	MB	79	-	-	-	-	-	-	-	-	-	-	-	
CHG34	*Trichoderma* sp.	CAG	41	-	-	-	-	-	24	-	**97**	**96**	**95**	**93**	Yes (AC)
		PDA	**94**	**100**	-	-	-	-	**100**	**92**	**98**	**99**	**99**	**98**	Yes (AC & AM)
CHG35	*Penicillium* sp.	CAG	**100**	**100**	**99**	**99**	**100**	**84**	-	21	72	**93**	**95**	64	Yes (AC)

Strain	Identification	Medium	MRSA	Efm	Ab	Ec	Kp	Psa	Ca	Cn	A375	A549	HCT116	MB231	Selected?
		PDA	**98**	74	**98**	**98**	**100**	**77**	-	-	**89**	**98**	**98**	72	Yes (AC)
CHG38	*Fusarium* sp.	CAG	**100**	**100**	-	-	-	-	**92**	**96**	**98**	65	**93**	40	Yes (AC & AM)
		PDA	**100**	**100**	-	-	-	-	**88**	-	56	-	24	-	Yes (AM)
CHG39	*Bacillus* sp.	GYM	**97**	**100**	-	-	-	-	-	-	69	73	48	69	
		MB	**96**	**94**	-	-	-	-	-	-	30	29	-	31	
CHG40	*Streptomyces* sp.	GYM	**100**	**100**	-	-	-	-	61	37	**98**	**99**	**98**	**99**	Yes (AC)
		MB	**100**	**87**	-	-	-	-	-	-	51	51	55	61	
CHG41	*Bacillus licheniformis*	GYM	69	-	-	-	-	-	-	-	62	33	38	51	
		MB	66	-	-	-	-	-	-	-	25	33	-	33	
CHG48	*Streptomyces* sp.	GYM	**100**	**100**	-	-	-	-	**100**	**97**	**98**	**92**	**88**	**93**	Yes (AC & AM)
		MB	**100**	**100**	-	-	-	-	-	72	71	69	71	74	
CHG49	Pleosporaceae	CAG	**99**	**84**	**97**	**100**	**100**	**100**	38	53	**99**	**95**	**98**	**94**	Yes (AC)
		PDA	**98**	68	**98**	**100**	**100**	**82**	28	-	**97**	**97**	**98**	53	Yes (AC)
CHG52	*Penicillium* sp.	CAG	**92**	**88**	-	-	-	-	-	-	30	-	33	-	
		PDA	**95**	**96**	-	-	-	-	-	-	-	-	20	-	
CHG53	*Trichoderma* sp.	CAG	-	-	-	-	-	-	-	-	-	-	-	-	
		PDA	-	-	-	-	-	-	-	-	-	-	-	-	
CHG56	*Aspergillus* sp.	CAG	67	-	-	-	-	-	-	-	22	26	34	46	
		PDA	70	-	-	-	-	-	-	-	30	37	34	59	
CHG59	*Tamaricicola* sp.	CAG	**92**	-	-	36	-	-	-	-	-	-	-	-	
		PDA	**91**	-	-	37	-	-	-	-	-	-	-	-	
CHG60	*Peroneutypa* sp.	CAG	35	-	-	33	-	-	-	-	-	47	35	46	
		PDA	32	-	-	-	-	-	-	-	-	33	36	25	
CHG64	*Streptomyces* sp.	GYM	**100**	**97**	-	-	-	-	-	-	**99**	**98**	**99**	**98**	Yes (AC)
		MB	**100**	**98**	-	-	-	-	-	-	-	58	23	50	
CKG5	*Nocardiopsis* sp.	GYM	-	46	-	-	-	-	-	-	29	-	48	-	
		MB	**84**	**94**	-	-	-	-	-	-	22	-	70	-	
CKG6	*Shewanella aestuarii*	MB	**100**	**94**	-	-	-	-	-	-	-	-	-	-	

Strain	Identification	Medium	MRSA	Efm	Ab	Ec	Kp	Psa	Ca	Cn	A375	A549	HCT116	MB231	Selected?
CKG7	*Vibrio* sp.	MB	34	-	-	-	-	-	-	-	-	-	-	-	
CKG12	*Vibrio* sp.	MB	**100**	74	-	-	-	-	-	-	-	-	-	-	
CKG13	*Pseudomonas* sp.	MB	35	-	-	-	-	-	-	-	-	-	-	-	
CKG15	*Shewanella colwelliana*	MB	**100**	**100**	-	-	-	-	-	-	-	-	-	-	
CKG16	*Eutypa lata*	CAG	46	-	-	31	-	-	-	-	-	26	34	33	
		PDA	-	-	-	29	-	-	-	-	-	33	26	32	
CKG20	*Micromonospora* sp.	GYM	**100**	**100**	-	-	-	-	-	-	**98**	**100**	**99**	**100**	Yes (AC)
		MB	53	-	-	-	-	-	-	-	-	-	-	-	
CKG21	*Bacillus hwajinpoensis*	GYM	65	-	-	-	-	-	-	-	-	20	-	-	
		MB	**100**	**100**	-	-	-	-	-	-	-	-	-	-	
CKG23	*Penicillium* sp.	CAG	**100**	**98**	**100**	**100**	**100**	**89**	-	-	**97**	**98**	**98**	**97**	Yes (AC)
		PDA	**100**	**100**	**98**	**100**	**100**	75	-	41	**95**	**98**	77	**92**	Yes (AC)
CKG24	*Bacillus* sp.	GYM	**100**	**100**	-	-	-	-	-	**94**	72	**98**	63	34	Yes (AC & AM)
		MB	**100**	**100**	-	-	-	-	-	-	51	54	43	59	
CKG25	*Galactomyces candidum*	CAG	**100**	**100**	**100**	**100**	**100**	**85**	-	-	**96**	**99**	**92**	**82**	Yes (AC)
		PDA	**99**	**99**	**100**	**100**	**100**	**93**	-	-	**98**	**99**	**99**	**93**	Yes (AC)
CKG31	*Shewanella kaireitica*	GYM	**100**	**100**	34	-	-	-	-	-	-	27	26	25	
		MB	**100**	**100**	-	-	-	-	-	-	-	20	-	-	
CKG32	*Fusarium* sp.	CAG	**100**	**100**	-	-	-	-	**89**	40	61	20	29	-	Yes (AM)
		PDA	**99**	**86**	-	-	-	-	60	-	21	-	-	-	
CKG33	*Mucor hiemalis*	CAG	62	-	-	-	-	-	-	-	-	27	27	48	
		PDA	77	-	-	-	-	-	-	-	-	-	-	-	
CKG37	*Sarocladium strictum*	CAG	**100**	**94**	-	38	-	-	-	-	-	-	-	-	
		PDA	**95**	47	-	41	-	-	-	-	78	48	58	56	
CKG38	*Pseudomonas anguilliseptica*	GYM	**100**	**100**	-	-	-	-	**85**	49	68	24	**91**	**88**	Yes (AC & AM)
		MB	**100**	**97**	-	-	-	-	**80**	73	-	-	-	-	Yes (AM)
CKG39	*Bacillus* sp.	GYM	-	-	-	-	-	-	-	-	26	-	-	-	
		MB	37	-	-	-	-	-	-	-	31	-	-	-	

Strain	Identification	Medium	MRSA	Efm	Ab	Ec	Kp	Psa	Ca	Cn	A375	A549	HCT116	MB231	Selected?
CKG42	*Elaphocordyceps* sp.	CAG	76	-	-	-	-	-	-	-	37	-	-	-	
		PDA	**82**	66	-	-	-	-	35	-	38	26	-	-	
CKG43	*Bacillus* sp.	GYM	**92**	**100**	-	-	-	-	-	-	38	-	24	-	
		MB	**94**	**100**	-	-	-	-	-	-	26	-	-	-	
CKG44	*Neonectria coccinea*	CAG	54	-	-	-	-	-	-	-	24	-	-	28	
		PDA	-	-	-	-	-	-	-	-	-	-	-	-	
CKG49	*Mucor circinelloides*	CAG	**89**	**98**	-	-	-	-	-	-	21	35	44	22	
		PDA	**100**	**100**	-	-	-	-	-	-	66	37	79	43	
CKG50	*Sporosarcina* sp.	GYM	**100**	**96**	-	-	-	-	-	-	-	-	-	-	
		MB	**100**	**98**	-	-	-	-	-	-	-	-	20	-	
CKG54	*Cordyceps farinosa*	CAG	**98**	**95**	-	-	-	-	-	-	56	-	30	-	
		PDA	**99**	**97**	-	-	-	-	-	-	52	24	42	-	
CKG57	*Arthopyrenia* sp.	CAG	66	-	-	-	-	-	-	-	-	-	-	-	
		PDA	52	-	-	-	-	-	-	-	-	-	-	-	
CKG58	*Nocardiopsis prasina*	GYM	**81**	**98**	-	-	-	-	**81**	54	**99**	**99**	**99**	**98**	Yes (AC & AM)
		MB	**100**	**99**	-	-	-	-	26	-	38	-	61	-	
CKG62	*Trichoderma* sp.	CAG	30	-	-	31	-	-	**88**	-	28	-	-	-	
		PDA	70	**100**	-	31	-	-	**93**	74	**98**	**98**	**99**	**98**	Yes (AC & AM)
CKG63	*Penicillium* sp.	CAG	**96**	25	26	-	-	21	36	-	45	50	30	64	
		PDA	76	22	**84**	71	**82**	59	-	-	51	**80**	49	77	Yes (AC)
CKG64	*Penicillium* sp.	CAG	-	-	-	-	37	-	-	-	-	21	-	37	
		PDA	70	-	-	-	-	-	-	-	-	34	30	45	
CKG66	*Acrostalagmus luteoalbus*	CAG	**100**	**100**	**86**	28	-	22	-	-	**87**	**89**	**83**	**89**	Yes (AC)
		PDA	**87**	51	-	-	-	-	-	-	25	-	70	-	
CKG70	*Plectosphaerella cucumerina*	CAG	56	-	-	-	-	-	-	-	-	-	-	-	
		PDA	69	-	-	-	-	-	-	-	-	-	-	-	

Table S3. Statistical comparison of chemically distinct bacterial crude extracts. ANOSIM was based on Euclidean distance. Group 1 included the following extracts: *Pseudomonas anguilliseptica* extracts CKG38-GYM and CKG38-MB and *Streptomyces* sp. extracts CHG40-GYM and CHG64-GYM.

Comparison	R value	p value
All	1	0.0001
Group 1 x Group 2 (CHG48-GYM, CKG58-GYM)	1	0.0022
Group 1 x Group 3 (CKG20-GYM)	1	0.0229
Group 1 x Group 4 (CKG24-GYM)	1	0.0232

Table S4. Statistical comparison of chemically distinct fungal crude extracts. ANOSIM was based on Euclidean distance. Group 1 included the following extracts: *Acrostalagmus luteoalbus* extract CKG66-CAG, *Galactomyces candidum* extracts CKG25-CAG and CKG25-PDA, *Penicillium* sp. extracts CHG25-CAG, CHG25-PDA, CHG35-CAG, CHG35-PDA, CKG23-CAG and CKG63-PDA and Pleosporaceae extracts CHG49-CAG and CHG49-PDA.

Comparison	R value	p value
All	0.8363	0.0001
Group 1 x Group 2 (CHG34-CAG, CHG34-PDA, CKG62-PDA)	0.9369	0.0001
Group 1 x Group 3 (CHG38-CAG, CHG38-PDA, CKG32-CAG)	0.6822	0.0001
Group 1 x Group 4 (CKG23-PDA)	0.9996	0.0055

Table S5. Putatively identified compounds produced by *Streptomyces* sp. extract CHG48-GYM. Putative annotations were based on the accurate mass, the predicted putative molecular formulae (MF), the retention time (R_t), the fragmentation pattern and the biological origin. *MF with best ppm error displayed; IC: Identification confidence level [3]; Nf: No fragmentation detected or below noise threshold ($5e^1$); Ref = reference(s).

No.	*m/z* value	Adduct	R_t (min)	ppm	Putative MF	Fragmentation pattern	IC	Putative identification	Chemical family	Biological origin	Ref
1	225.1104	$[M+H]^+$	3.07	1.8	$C_8H_{12}N_6O_2$	181.1201, 164.1114, 141.9593, 97.9700	4	n.a.			
2	208.0976	$[M+H]^+$	3.1	1	$C_{11}H_{13}NO_3$	190.0865, 166.0866, 164.1073, 146.0968, 135.0804, 131.0732, 122.0962, 118.0657	3	Streptazolin	Oxazolidone alkaloid	*Streptomyces viridochromogenes*	[4]
3	250.1422	$[M+Na]^+$	3.53	1.2	$C_{12}H_{21}NO_3$	138.053	3	Streptenol E	Acetamide	*Streptomyces* sp.	[5]
4	239.1261	$[M+H]^+$	3.72	2.1	$C_9H_{14}N_6O_2$	216.0758, 210.1890, 198.0604, 195.1371, 172.0880, 155.0949, 141.9587	4	n.a.			
5	620.235	$[M+H]^+$	3.96	1.1	$C_{30}H_{37}NO_{13}$	142.1230, 98.0971	4	n.a.			
6	604.2402	$[M+H]^+$	4.04	1.3	$C_{30}H_{37}NO_{12}$	572.0979, 142.1230, 124.1113, 98.0970, 79.0555	3	Platensimycin B4	Diterpenoid glycoside	*Streptomyces platensis*	[6]
7	366.1893	$[M+Na]^+$	4.12	0	$C_{17}H_{29}NO_6$	308.1821, 290.1734, 270.1647, 252.1608, 224.1649, 198.0766, 180.0642, 172.0989, 166.1217, 154.1225, 152.0704, 142.0490, 137.0591, 114.0552, 109.1009	3	Alpiniamide A	Linear polyketide	*Streptomyces* sp.	[7]
8	253.1415	$[M+H]^+$	4.28	0.8	$C_{10}H_{17}N_6O_2$	228.0977, 209.1505, 205.6684, 186.9429, 182.0986, 165.6180	4	n.a.			
9	253.142	$[M+H]^+$	4.44	2.8	$C_{10}H_{17}N_6O_2$	210.0668, 195.9134, 170.9937	4	n.a.			
10	308.1867	$[M-H_2O]^+$	4.76	1.6	$C_{17}H_{27}NO_5$	252.1580, 198.0758, 180.0665, 172.0976, 166.1222, 152.0706, 142.0513, 137.0609, 109.1020	4	n.a.			
11	713.2711	$[M+H]^+$	5.5	0.1	$C_{39}H_{40}N_2O_{11}$	142.1232, 98.0974	4	n.a.			
12	387.238	$[M+H]^+$	5.7	-0.8	$C_{20}H_{34}O_7$	165.1138, 167.1079, 143.0687, 125.0604, 121.1022, 111.0808, 93.0695	2	Nonactyl nonactoate	Nonactic acid polyketide	*Streptomyces* sp.	[8]
13	558.1771	$[M+H]^+$	6.01	-1.1	$C_{32}H_{23}N_5O_5$*	174.0918, 162.0919	4	n.a.			
14	401.254	$[M+H]^+$	6.24	0.2	$C_{21}H_{36}O_7$	199.1332, 181.1228, 167.1066, 143.0703, 125.0966, 111.0810, 107.0859	2	Bonactin	Nonactic acid polyketide	*Streptomyces* sp.	[9]
15	421.2201	$[M+Na]^+$	6.35	0.9	$C_{21}H_{34}O_7$	239.1254, 223.0950	4	n.a.			
16	413.2513	$[M+Na]^+$	6.42	-0.5	$C_{20}H_{38}O_7$*	227.1261, 209.1144	4	n.a.			
17	309.1684	$[M+H]^+$	6.62	2.9	$C_{13}H_{20}N_6O_3$	265.1174, 221.1151, 207.0990	4	n.a.			
18	415.2705	$[M+H]^+$	6.82	2.2	$C_{22}H_{38}O_7$	199.1374, 181.1226, 163.1117, 143.0700, 139.1125, 135.1168,125.0959, 107.0853, 81.0709	2	Homononactyl homononactate	Nonactic acid polyketide	*Streptomyces griseus*	[10]
19	321.1682	$[M+H]^+$	6.89	2.2	$C_{14}H_{20}N_6O_3$	221.1156	4	n.a.			

No.	*m/z* value	Adduct	R_t (min)	ppm	Putative MF	Fragmentation pattern	IC	Putative identification	Chemical family	Biological origin	Ref
20	312.1967	$[M+H]^+$	7.49	1	$C_{20}H_{25}NO_2$	216.1390, 200.1078, 188.1083, 172.1142, 162.0898, 151.1139	4	n.a.			
21	807.395	$[M+H]^+$	7.73	-2.4	$C_{46}H_{54}N_4O_9$	789.3856, 771.2729, 434.1707, 396.1556, 378.1438, 336.1355, 297.1229, 285.1239	4	n.a.			
22	289.175	$[M+Na]^+$	7.93	-1	$C_{12}H_{22}N_6O$	Nf	4	n.a.			
23	585.3634	$[M+H]^+$	8.12	-0.9	$C_{31}H_{52}O_{10}$	199.1380, 185.1173, 181.1227, 167.1063, 143.0731, 125.0962, 111.0792, 93.0702	4	n.a.			
24	352.1552	$[M+H]^+$	8.4	0.9	$C_{21}H_{21}NO_4$	174.0921, 162.0919	4	n.a.			
25	807.3981	$[M+H]^+$	8.56	1.5	$C_{46}H_{54}N_4O_9$	789.3876, 771.2750, 434.1710, 396.1559, 378.1448, 365.1138, 336.1154, 297.1230, 285.1233	4	n.a.			
26	621.3619	$[M+Na]^+$	8.6	0.6	$C_{32}H_{54}O_{10}$	423.2351, 419.2396, 225.1102, 221.1153	4	n.a.			
27	684.3649	$[M+H]^+$	8.87	0	$C_{40}H_{49}N_3O_7$*	273.1241, 174.0922, 162.0919	4	n.a.			
28	560.4683	$[M+H]^+$	9.06	0.7	$C_{35}H_{61}NO_2$	542.4562, 524.4463	4	n.a.			
29	546.4896	$[M+H]^+$	9.87	1.8	$C_{35}H_{63}NO_3$	528.4788, 510.4700	4	n.a.			
30	783.4903	$[M+H]^+$	10.09	1	$C_{42}H_{70}O_{13}$	199.1337, 185.1174, 181.1226, 167.1068, 143.0703, 125.0961, 111.0809	4	n.a.			
31	797.5042	$[M+H]^+$	10.48	-1.1	$C_{43}H_{72}O_{13}$	199.1342, 181.1236, 167.1073, 143.0718, 125.0973, 107.0860	4	n.a.			
32	811.5203	$[M+H]^+$	10.85	-0.6	$C_{44}H_{74}O_{13}$	Nf	4	n.a.			
33	737.4476	$[M+H]^+$	11.77	0	$C_{40}H_{64}O_{12}$	MS6: 185.1191, 167.1073, 149.0946, 143.0683, 121.1022, 111.0811	2	Nonactin	Nonactic acid polyketide	*Streptomyces* spp.	[11]

Table S6. Putatively identified compounds produced by *Micromonospora* sp. extract CKG20-GYM. Putative annotations were based on the accurate mass, the predicted putative molecular formulae (MF), the retention time (R_t), the fragmentation pattern and the biological origin. $^{\Delta}$Different isomers with same *m/z* value and molecular formula, which cannot be differentiated based on MS/MS data; IC: Identification confidence level [3]; Ref = reference(s).

No.	*m/z* value	Adduct	R_t (min)	ppm	Putative MF	Fragmentation pattern	IC	Putative identification	Chemical family	Biological origin	Ref
34	243.1348	$[M+H]^+$	2.47	1.2	$C_{11}H_{18}N_2O_4$	201.1239, 165.1032, 154.0877, 137.1081, 100.0399	4	n.a.			
35	218.1417	$[M+H]^+$	3.2	5	$C_{11}H_{15}N_5$	162.9784, 150.0785	4	n.a.			
36	280.124	$[M+H]^+$	4.17	-6.1	$C_8H_{17}N_5O_6$	262.1132, 196.0661	4	n.a.			
37	197.118	$[M+H]^+$	5.67	1	$C_{11}H_{16}O_3$	158.9618, 117.9348, 96.9611	4	n.a.			
38	420.3119	$[M-H_2O]^+$	6.8	1.2	$C_{25}H_{43}NO_5$	378.2987, 332.2931, 315.2679, 229.1942, 203.1753, 175.1504, 149.1352, 135.1159, 107.0873	4	n.a.			
39	369.218	$[M+H]^+$	7.48	0.5	$C_{22}H_{28}N_2O_3$	256.0851	4	n.a.			
40	479.2547	$[M+H]^+$	7.72	0.2	$C_{28}H_{34}N_2O_5$	287.0663, 275.0670, 259.0480	4	n.a.			
41	466.2933	$[M+H]^+$	7.85	-0.2	$C_{22}H_{44}NO_7P$	325.2744, 294.2796	4	n.a.			
42	454.2929	$[M+H]^+$	8.08	-1.1	$C_{21}H_{44}NO_7P$	313.2740, 282.2794	2	1-palmitoyl-2-hydroxy-sn-glycero-3-phosphoethanolamine	Glycero-phospholipid	In cell membranes of all organisms	
43	330.2435	$[M+H]^+$	8.11	0.6	$C_{21}H_{31}NO_2$	312.2322	4	n.a.			
44	463.2597	$[M+H]^+$	8.2	0	$C_{28}H_{34}N_2O_4$	271.0725, 259.0725, 243.0536	2	Diazepinomicin	Phenazine alkaloid	*Micromonospora* sp.	[12]
45	480.3098	$[M+H]^+$	8.38, 8.51$^{\Delta}$	1.7	$C_{23}H_{46}NO_7P$	339.2955, 308.2907	2	1-(9Z-octadecenoyl)-sn-glycero-3-phosphoethanolamine	Glycero-phospholipid	In cell membranes of all organisms	
46	479.2547	$[M+H]^+$	8.62	0.2	$C_{28}H_{34}N_2O_5$	258.0409, 240.0301, 146.0241, 112.0401	4	n.a.			
47	408.0832	$[M+H]^+$	8.78	0	$C_{20}H_{13}N_3O_7$	390.0728, 372.0609, 362.0768, 349.0473, 344.0648, 321.0529, 259.0353, 245.0565, 233.0556, 176.0353	4	n.a.			
48	411.2281	$[M+H]^+$	8.9	-0.7	$C_{24}H_{30}N_2O_4$	393.2157, 383.2320, 285.0869, 271.0714, 259.0710, 243.0767	3	Diazaquinomycin D	Phenazine alkaloid	*Streptomyces* sp.	[13]
49	619.4077	$[M+H]^+$	8.93	1	$C_{33}H_{54}N_4O_7$	427.3684, 400.3203, 376.3233, 305.2831, 297.1194, 280.0945, 279.1076, 262.0820, 252.0987, 224.1024, 220.0929, 208.1091, 202.0814, 193.0606, 167.0832, 149.0701, 123.0920, 121.0773, 109.1015, 96.0452	4	n.a.			

No.	*m*/*z* value	Adduct	R_t (min)	ppm	Putative MF	Fragmentation pattern	IC	Putative identification	Chemical family	Biological origin	Ref
50	482.325	$[M+H]^+$	9.09	0.8	$C_{23}H_{48}NO_7P$	341.3052, 310.3102	2	1-stearoyl-2-hydroxy-sn-glycero-3-phosphoethanolamine	Glycero-phospholipid	In cell membranes of all organisms	
51	607.4077	$[M+H]^+$	9.27	1	$C_{32}H_{54}N_4O_7$	388.3211, 297.1197, 293.2846, 280.0937, 252.0986, 220.0934, 202.0833, 167.0821, 149.0676, 121.0761	2	Rakicidin A	Cyclic depsipeptide	*Micromonospora* sp.	[14]
52	621.4227	$[M+H]^+$	9.73	0	$C_{33}H_{56}N_4O_7$	402.3372, 350.3409, 307.2997, 297.1193, 280.0944, 252.0985, 220.0929, 167.0816, 149.0691, 121.0767	2	Rakicidin B	Cyclic depsipeptide	*Micromonospora* sp.	
53	635.4383	$[M+H]^+$	10.19	-0.2	$C_{34}H_{58}N_4O_7$	416.3523, 364.3604, 321.3148, 297.1199, 280.0939, 252.0980, 220.0930, 202.0839, 167.0817, 149.0708, 121.0757	2	Rakicidin E	Cyclic depsipeptide	*Micromonospora* sp.	[15]

Table S7. Putatively identified compounds produced by *Bacillus* sp. extract CKG24-GYM. Putative annotations were based on the accurate mass, the predicted putative molecular formulae (MF), the retention time (R_t), the fragmentation pattern and the biological origin. ±Parent mass out of detection limit (>1200 Da) and therefore, the ppm error and fragmentation pattern were not determined; *MF with best ppm error displayed; IC: Identification confidence level [3]; Nf: No fragmentation detected or below noise threshold ($5e^1$); Ref = reference(s).

No.	*m/z* value	Adduct	R_t (min)	ppm	Putative MF	Fragmentation pattern	IC	Putative identification	Chemical family	Biological origin	Ref
54	367.0508	$[M+H]^+$	3.63	-1.4	$C_{12}H_{14}O_{13}$	287.0525, 258.0562, 241.9696, 238.0435, 188.0169, 168.9262, 164.9999, 146.9907	4	n.a.			
55	424.2082	$[M+H]^+$	3.71	-0.5	$C_{20}H_{29}N_3O_7$	407.1817, 390.1540, 330.1343, 274.0708, 250.1446, 232.1337, 215.1069, 159.0442	2	Amicoumacin-A	Isocoumarin	*Bacillus subtilis*	[16]
56	439.2083	$[M+H]^+$	4.12	0.7	$C_{21}H_{30}N_2O_8$	422.1824, 250.1440, 232.1337, 215.1070, 176.0702, 159.0444, 149.0598	4	n.a.			
57	255.1207	$[M+H]^+$	4.25	0.4	$C_9H_{14}N_6O_3$	195.9121	4	n.a.			
58	250.1127	$[M+H]^+$	5.04	-4.4	$C_5H_{14}N_8O_4$	194.0496, 182.0501	4	n.a.			
59	392.1711	$[M+H]^+$	5.43	0.5	$C_{20}H_{25}NO_7$	276.1247, 250.1441, 232.1328, 215.1074, 159.0440, 149.0598, 125.0238	3	Bacillcoumacin D	Isocoumarin	*Bacillus* sp.	[17]
60	390.1556	$[M+H]^+$	5.55	0.8	$C_{20}H_{23}NO_7$	250.1441, 232.1340, 215.1073, 159.0442, 123.0087	3	Antibiotic AI-77-F or -H	Isocoumarin	*Bacillus* spp.	F: [18], H: [19]
61	1071.5811	$[M+H]^+$	6.05	-2.8	$C_{50}H_{78}N_{12}O_{14}$	535.3573, 455.3136, 437.3194, 392.1531, 354.2751, 341.2750, 323.2671, 313.1513, 299.2645, 295.1353, 278.1133, 260.1039, 250.1231, 212.1082, 208.1156, 198.2230, 167.0809, 136.0747	3	Bacillomycin F2	Cyclic lipopeptide	*Bacillus subtilis*	[20], [21]
62	1085.5974	$[M+H]^+$	6.45	-1.9	$C_{51}H_{80}N_{12}O_{14}$	680.4345, 663.4116, 645.3961, 618.3965, 566.3553, 549.3728, 531.3541, 507.3527, 469.3383, 451.3282, 406.1735, 392.1518, 389.1490, 375.1311, 368.2910, 355.2957, 351.2631, 323.1372, 313.2847, 295.1401, 278.1146, 277.1303, 275.1039, 268.2650, 264.0987, 261.0894, 260.1024, 250.1191, 243.1082, 233.0928, 216.0988, 212.2379, 209.0939, 208.1094, 198.0890, 188.1048, 184.1085, 167.0820, 136.0761	3	Bacillomycin F3	Cyclic lipopeptide	*Bacillus subtilis*	[20], [21]
63	1099.6122	$[M+H]^+$	6.88	-2.7	$C_{52}H_{82}N_{12}O_{14}$	694.4479, 677.4232, 660.3998, 632.4120, 580.4044, 563.3820, 545.3659, 483.3513, 465.3430, 406.1723, 392.1563, 389.1466, 382.3062, 375.1307, 369.3107, 365.2799, 351.2995, 337.2898, 327.3002, 323.1351, 313.1502, 309.2888, 295.1408, 278.1149, 275.1031, 264.0986, 261.0858, 250.1187, 233.0918, 226.2534, 212.1035, 209.0947, 188.1048, 184.1090, 167.0815, 136.0763	3	Bacillomycin F5	Cyclic lipopeptide	*Bacillus subtilis*	[20], [21]
64	781.4157	$[M+H]^+$	7.08	-0.8	$C_{44}H_{60}O_{12}$	557.3537, 539.3503, 419.2196, 405.2093, 401.2106, 399.1993, 389.2074, 373.2114, 371.2022, 367.1888, 365.1761, 359.2000, 351.1960, 349.1813, 343.2067, 341.1881, 335.2000, 333.1837, 331.1708, 323.1975, 321.1785, 305.1885, 303.1775, 209.0880,	3	Aurantinin B	Polyketide glycoside	*Bacillus aurantinus*	[22]

No.	*m/z* value	Adduct	R_t (min)	ppm	Putative MF	Fragmentation pattern	IC	Putative identification	Chemical family	Biological origin	Ref
						173.1329, 171.1176, 161.1315, 159.1180, 147.1168, 145.1039, 121.1011, 119.0865					
65	746.4219	$[M+2H]^{2+}$	7.14	n.a.±	$C_{74}H_{114}N_{12}O_{20}$	n.a.±	3	SNA 60-367-5	Cyclic lipopeptide	*Bacillus* sp.	[23]
66	739.4143	$[M+2H]^{2+}$	7.14	n.a.±	$C_{73}H_{112}N_{12}O_{20}$	n.a.±	3	SNA 60-367-6	Cyclic lipopeptide	*Bacillus* sp.	[23]
67	663.3561	$[M+H]^{+}$	7.24	-0.6	$C_{26}H_{38}N_{20}O_2$ or $C_{28}H_{50}N_6O_{12}$*	501.3020, 421.1306, 365.1053	4	n.a.			
68	753.4296	$[M+2H]^{2+}$	7.37	n.a.±	$C_{75}H_{116}N_{12}O_{20}$	n.a.±	3	a: SNA 60-367-12, b: SNA 60-367-13	Cyclic lipopeptide	*Bacillus* sp.	[23]
69	738.4238	$[M+2H]^{2+}$	7.56	n.a.±	$C_{74}H_{114}N_{12}O_{19}$	n.a.±	3	SNA 60-367-17	Cyclic lipopeptide	*Bacillus* sp.	[23]
70	738.4249	$[M+2H]^{2+}$	7.64	n.a.±	$C_{74}H_{114}N_{12}O_{19}$	n.a.±	3	SNA 60-367-18	Cyclic lipopeptide	*Bacillus* sp.	[23]
71	731.4171	$[M+2H]^{2+}$	7.64	n.a.±	$C_{73}H_{112}N_{12}O_{19}$	n.a.±	3	SNA 60-367-19	Cyclic lipopeptide	*Bacillus* sp.	[23]
72	745.4335	$[M+2H]^{2+}$	7.82	n.a.±	$C_{75}H_{116}N_{12}O_{19}$	n.a.±	3	SNA 60-367-23	Cyclic lipopeptide	*Bacillus* sp.	[23]
73	512.3693	$[M+H]^{+}$	8.25	-1.4	$C_{27}H_{49}N_3O_6$ or $C_{24}H_{41}N_{13}$*	268.2627, 115.0873, 102.0553, 84.0438	4	n.a.			
74	1008.6603	$[M+H]^{+}$	10.24	0.6	$C_{51}H_{89}N_7O_{13}$	455.3112, 441.2708, 437.3002, 427.3160, 409.3047, 395.2667, 342.2245, 328.1873, 324.2168, 314.2333, 283.2020, 245.1858, 229.1185, 227.1759, 212.2019, 201.1239, 199.1811, 195.1743, 185.1657, 183.1133, 154.1599, 86.0973	3	Anteiso-C_{13}-[Leu7]-surfactin	Cyclic lipopeptide	*Bacillus pumilus*	[24]
75	1064.5789	$[M+H]^{+}$	10.29	0.4	$C_{48}H_{74}N_{19}O_7Cl$ or $C_{50}H_{86}N_5O_{17}Cl$*	Nf	4	n.a.			
76	994.6426	$[M+H]^{+}$	10.41	-1.4	$C_{50}H_{87}N_7O_{13}$	455.3094, 441.2723, 437.2990, 342.2255, 328.1864, 324.2165, 314.2322, 296.2226, 285.1454, 283.2004, 269.1878, 231.1687, 229.1176, 227.1760, 215.1026, 212.2016, 201.1236, 199.1812, 195.1743, 185.1639, 86.0975	3	Lipopeptide NO	Cyclic lipopeptide	*Bacillus subtilis*	[25]
77	1022.6801	$[M+H]^{+}$	10.64	4.7	$C_{52}H_{91}N_7O_{13}$	469.3278, 451.3170, 441.2706, 423.3218, 395.2657, 356.2436, 342.2031, 338.2332, 328.1876, 310.2383, 296.1983, 285.1457, 283.2006, 269.1866, 267.2441, 255.1708, 245.1868, 229.1192, 227.1763, 215.1034, 213.1604, 209.1902, 201.1241, 199.1811, 185.1653, 183.1132, 170.1181, 154.1593, 86.0974	3	Iso-C_{14}-[Val7]-surfactin	Cyclic lipopeptide	*Bacillus pumilus*	[24]

No.	*m/z* value	Adduct	R_t (min)	ppm	Putative MF	Fragmentation pattern	IC	Putative identification	Chemical family	Biological origin	Ref
78	1078.5947	$[M+H]^+$	10.66	0.5	$C_{51}H_{88}N_5O_{17}Cl$ or $C_{49}H_{76}N_{19}O_7Cl$*	Nf	4	n.a.			
79	1022.6746	$[M+H]^+$	10.76	-0.7	$C_{52}H_{91}N_7O_{13}$	469.3280, 451.3169, 441.2711, 423.3229, 395.2647, 356.2435, 342.2023, 338.2332, 310.2377, 296.1992, 285.1464, 283.2021, 269.1866, 267.2444, 255.1700, 245.1868, 243.1360, 229.1189, 227.1763, 215.1030, 213.1584, 209.1903, 201.1242, 199.1811, 185.1656, 183.1134, 154.1593, 86.0974	3	n-C_{14}-[Val7]-surfactin	Cyclic lipopeptide	*Bacillus pumilus*	[24]
80	1064.5792	$[M+H]^+$	10.78	0.7	$C_{48}H_{74}N_{19}O_7Cl$ or $C_{50}H_{86}N_5O_{17}Cl$*	Nf	4	n.a.			
81	1008.6598	$[M+H]^+$	10.8	0.1	$C_{51}H_{89}N_7O_{13}$	469.3280, 451.3174, 441.2707, 427.3165, 395.2650, 356.2438, 342.2028, 338.2325, 328.1869, 310.2381, 296.1968, 285.1455, 283.2013, 269.1865, 255.1698, 253.2272, 243.1342, 231.1708, 229.1192, 227.1756, 213.1611, 201.1238, 199.1807, 185.1652, 86.0971	3	n-C_{13}-[Leu7]-surfactin	Cyclic lipopeptide	*Bacillus pumilus*	[24]
82	1036.692	$[M+H]^+$	10.95	1	$C_{53}H_{93}N_7O_{13}$	483.3431, 465.3328, 455.3480, 441.2714, 437.3377, 395.2664, 370.2592, 352.2489, 342.2029, 328.1875, 324.2541, 311.1967, 296.1978, 285.1458, 283.2004, 269.1868, 267.2441, 255.1712, 253.2282, 245.1872, 240.2331, 229.1195, 227.1764, 215.1037, 213.1607, 201.1243, 199.1815, 185.1657, 183.1137, 170.1186, 154.1597, 86.0977	3	Anteiso-C_{15}-[Leu7]-surfactin	Cyclic lipopeptide	*Bacillus pumilus*	[24]
83	1092.6073	$[M+H]^+$	10.97	0.1	$C_{46}H_{74}N_{25}O_5Cl$ or $C_{48}H_{86}N_{11}O_{15}Cl$*	Nf	4	n.a.			
84	1022.6751	$[M+H]^+$	11.16	-0.2	$C_{52}H_{91}N_7O_{13}$	483.3442, 465.3320, 441.2701, 395.2653, 370.2591, 356.2491, 342.2023, 328.1870, 324.2528, 296.1963, 285.1446, 283.2008, 269.1873, 255.1707, 240.2331, 231.1708, 229.1192, 227.1765, 215.1028, 213.1597, 201.1245, 199.1814, 185.1651, 183.1138, 86.0979	3	Anteiso-C_{15}-[Val7]-surfactin	Cyclic lipopeptide	*Bacillus pumilus*	[24]
85	1036.6903	$[M+H]^+$	11.18	-1.2	$C_{53}H_{93}N_7O_{13}$	483.3438, 465.3333, 455.3474, 441.2715, 437.3364, 395.2658, 370.2595, 352.2492, 342.2029, 328.1875, 324.2541, 311.1971, 296.1976, 285.1454, 283.2013, 269.1869, 267.2441, 255.1713, 245.1871, 240.2327, 229.1191, 227.1764, 223.2063, 215.1031, 213.1602, 201.1242, 199.1816, 185.1657, 183.1132, 170.1179, 154.1593, 86.0977	3	n-C_{14}-[Leu7]-surfactin	Cyclic lipopeptide	*Bacillus pumilus*	[24]
86	1036.687	$[M+H]^+$	11.48	-3.8	$C_{53}H_{93}N_7O_{13}$	497.3574, 483.3420, 479.3447, 465.3322, 455.3483, 441.2713, 395.2702, 384.2736, 370.2583, 366.2633, 356.2750, 352.2485, 342.2026, 338.2686, 326.2470, 324.2563, 311.1976, 296.1982,	3	Anteiso-C_{15}-[Ile7]-surfactin	Cyclic lipopeptide	*Bacillus pumilus*	[24]

No.	*m/z* value	Adduct	R_t (min)	ppm	Putative MF	Fragmentation pattern	IC	Putative identification	Chemical family	Biological origin	Ref
						285.1438, 283.2013, 269.1826, 267.2478, 254.2482, 245.1890, 243.1348, 231.1699, 229.1181, 227.1755, 215.1025, 213.1602, 201.1240, 199.1809, 185.1648, 183.1124, 170.1178, 86.0976					
87	1064.7209	$[M+H]^+$	11.65	-1.3	$C_{55}H_{97}N_7O_{13}$	511.3743, 493.3648, 483.3786, 441.2816, 398.2912, 395.2643, 380.2790, 352.2855, 342.2029, 328.1868, 296.1967, 285.1422, 283.2024, 268.2642, 255.1717, 245.1867, 233.2294, 229.1197, 227.1754, 215.1038, 201.1240, 199.1813, 185.1668, 183.1134, 154.1599, 86.0986	3	KMM 1364E	Cyclic lipopeptide	*Bacillus pumilus*	[26]

Table S8. Putatively identified compounds produced by *Trichoderma* sp. extracts CHG34-CAG and CHG34-PDA. Putative annotations were based on the accurate mass, the predicted putative molecular formulae (MF), the retention time (R_t), the fragmentation pattern and the biological origin. $^{\Delta}$Different isomers with same *m/z* value and molecular formula, which cannot be differentiated based on MS/MS data; $^{\pm}$Parent mass out of detection limit (>1200 Da) and therefore, the ppm error and fragmentation pattern were not determined; *MF with best ppm error displayed; IC: Identification confidence level [3]; Nf: No fragmentation detected or below noise threshold ($5e^1$); Ref = reference(s).

No.	*m/z* value	Adduct	R_t (min)	ppm	Putative MF	Fragmentation pattern	IC	Putative identification	Chemical family	Biological origin	Medium	Ref
88	237.1132	$[M+H]^+$	4.51	2.1	$C_{13}H_{16}O_4$	195.9131, 167.4925, 165.0561, 141.9551, 139.0753, 125.0237, 123.0437, 113.0982	3	Trichosorbicillin E	Sorbicillinoid	*Trichoderma reesei*	CAG, PDA	[27]
89	410.218	$[M+H]^+$	5.22	0.2	$C_{21}H_{31}NO_7$	242.0663, 224.0546, 124.0363	4	n.a.			CAG	
90	562.3585	$[M+Na]^+$	5.23	0.7	$C_{27}H_{49}N_5O_6$*	320.1957	4	n.a.			PDA	
91	473.1602	$[M+H]^+$	5.42	0.4	$C_{28}H_{24}O_7$	455.1462, 445.1667, 399.1595, 371.1277, 367.1292, 353.1223, 343.1312, 325.1264, 321.1139, 315.1418, 301.1224, 293.1167, 279.0644, 277.1200, 275.0994, 269.0819, 265.1236, 253.0858, 247.1097, 243.1052, 241.0905, 237.0936, 209.0972	4	n.a.			CAG	
92	451.2694	$[M+H]^+$	5.52	-0.4	$C_{25}H_{38}O_7$	289.2172, 271.2065, 217.1958, 215.1443, 205.1220, 197.1335, 187.1477, 185.1332, 182.1101, 171.1170, 169.1024, 159.1187, 157.1004, 155.0854, 151.1133, 147.1177, 145.1024, 137.0966, 133.1015, 131.0863, 127.0398, 119.0860, 105.0706, 99.0446	4	n.a.			PDA	
93	473.1603	$[M+H]^+$	5.69	0.6	$C_{28}H_{24}O_7$	455.1467, 445.1640, 427.1577, 399.1595, 381.1467, 371.1253, 353.1160, 343.1339, 325.1219, 321.1107, 303.1033, 293.1172, 279.0655, 275.1062, 269.0769, 265.1217, 253.0830, 247.1122, 243.0975, 241.0873, 209.0973	4	n.a.			CAG	
94	391.2457	$[M+Na]^+$	5.96	-0.8	$C_{21}H_{36}O_5$	359.219	4	n.a.			CAG	
95	259.1335	$[M-H_2O]^+$	6.36	0.4	$C_{16}H_{20}O_4$	241.1224, 223.1130, 213.1285, 197.1323, 195.1142, 185.1317, 180.0920, 171.1167, 169.1014, 167.0865, 165.0684, 157.1010, 155.0842, 145.1008, 143.0855, 141.0714, 129.0699, 105.0713	4	n.a.			CAG	
96	277.144	$[M+H]^+$	6.6	0	$C_{16}H_{20}O_4$	231.1372, 201.0457, 195.9114, 143.0857, 129.0681	4	n.a.			CAG	

No.	*m/z* value	Adduct	R_t (min)	ppm	Putative MF	Fragmentation pattern	IC	Putative identification	Chemical family	Biological origin	Medium	Ref
97	331.1521	$[M+H]^+$	6.76	0.6	$C_{15}H_{18}N_6O_3$	299.1237, 211.1088, 189.1274, 133.0657	4	n.a.			CAG	
98	588.39	$[M+H]^+$	6.86	0	$C_{34}H_{53}NO_7$*	423.2950, 253.1597, 251.1447, 235.1489, 225.1640, 223.1465, 148.0973, 130.0874, 102.0919	4	n.a.			CAG, PDA	
99	189.1278	$[M+H]^+$	6.87	0.5	$C_{13}H_{16}O$	171.1169, 147.1166, 145.1010, 133.0649, 105.0698	4	n.a.			CAG	
100	439.3324	$[M+H]^+$	7.11	-0.2	$C_{28}H_{42}N_2O_2$	279.2345, 209.1549, 173.1328, 161.1080, 149.1325, 137.1328, 109.1021, 95.0867, 81.0709	4	n.a.			CAG, PDA	
101	344.3164	$[M+H]^+$	7.4	-0.3	$C_{20}H_{41}NO_3$	344.3129, 300.2899, 282.2793, 270.2758, 264.2762, 252.2674, 88.0771	4	n.a.			CAG, PDA	
102	345.1677	$[M+Na]^+$	7.76	-0.3	$C_{18}H_{26}O_5$	Nf	4	n.a.			CAG	
103	770.5386	$[M+H]^+$	7.79	-0.8	$C_{38}H_{71}N_7O_9$	453.3084, 354.2396, 326.2451, 300.2278, 241.1562, 184.1345, 143.1185, 86.0972	4	n.a.			CAG, PDA	
104	1197.756	$[M+Na]^+$	7.93	-2.3	$C_{58}H_{102}N_{12}O_{13}$	983.5846, 955.5931,897.5527, 870.5408, 843.5667, 757.4554, 730.4795, 645.4308, 547.3153, 489.2869, 462.2697, 403.2706	3	Trichodermide C	Peptaibol	*Trichoderma viride*	CAG, PDA	[28]
105	754.5424	$[M+H]^+$	8.12	-2.4	$C_{38}H_{71}N_7O_8$	453.2956, 354.2416, 326.2400, 241.1605, 184.1328, 86.0979	3	n.a.			CAG, PDA	
106	498.379	$[M+H]^+$	8.29	-1	$C_{28}H_{51}NO_6$*	480.3684, 236.1504	4	n.a.			CAG, PDA	
107	751.9497	$[M+2Na]^{2+}$	8.29	-0.7	$C_{71}H_{123}N_{15}O_{17}$	n.a.±	3	Tv29-14S-Vc	Peptaibol	*Trichoderma virens*	CAG, PDA	[29]
108	836.4872	$[M+H]^+$	8.29	0.5	$C_{38}H_{69}N_5O_{15}$ or $C_{36}H_{57}N_{19}O_5$*	369.2141, 256.1304	4	n.a.			CAG, PDA	
109	623.4496	$[M+H]^+$	8.29	0	$C_{32}H_{58}N_6O_6$*	324.2295, 215.1763, 211.1452, 183.1501	4	n.a.			CAG, PDA	
110	1197.7583	$[M+Na]^+$	8.39	-0.3	$C_{58}H_{102}N_{12}O_{13}$	1179.7465, 955.5943, 897.5522, 870.5418, 757.4579, 645.4301, 631.4155, 561.3373, 547.3219	3	Trichorozin-II	Peptaibol	*Trichoderma harzianum*	CAG, PDA	[30]
111	553.3351	$[M+H]^+$	8.41	0.2	$C_{26}H_{44}N_6O_7$*	355.1985, 256.1306, 228.1352, 128.0710, 101.0719	4	n.a.			PDA	
112	623.4495	$[M+H]^+$	8.41	-0.2	$C_{32}H_{58}N_6O_6$*	324.2296, 215.1762, 211.1454, 183.1503	4	n.a.			CAG, PDA	

No.	*m/z* value	Adduct	R_t (min)	ppm	Putative MF	Fragmentation pattern	IC	Putative identification	Chemical family	Biological origin	Medium	Ref
113	961.6072	$[M+H]^+$	8.41	-0.1	$C_{44}H_{72}N_{20}O_5$ or $C_{46}H_{84}N_6O_{15}$*	355.1985, 256.1304	4	n.a.			CAG, PDA	
114	345.168	$[M+Na]^+$	8.45	0.6	$C_{18}H_{26}O_5$	Nf	4	n.a.			CAG	
115	623.4493	$[M+H]^+$	8.45	-0.5	$C_{32}H_{58}N_6O_6$*	324.2290, 215.1765, 211.1449, 183.1499	4	n.a.			CAG, PDA	
116	553.335	$[M+H]^+$	8.45	0	$C_{26}H_{44}N_6O_7$*	355.1923, 256.1304, 228.1354, 128.0707, 101.0713	4	n.a.			CAG, PDA	
117	837.4725	$[M+H]^+$	8.45	0.4	$C_{39}H_{64}N_8O_{12}$ or $C_{37}H_{52}N_{22}O_2$*	370.1981, 257.1146, 128.0712	4	n.a.			CAG, PDA	
118	623.4493	$[M+H]^+$	8.52	-0.5	$C_{32}H_{58}N_6O_6$*	324.2288, 215.1760, 211.1449, 183.1497	4	n.a.			CAG, PDA	
119	758.9578	$[M+2Na]^{2+}$	8.52	-0.4	$C_{72}H_{125}N_{15}O_{17}$	n.a.±	3	Tv29-14S-VI	Peptaibol	*Trichoderma virens*	CAG, PDA	[29]
120	850.5023	$[M+H]^+$	8.52	-0.2	$C_{39}H_{71}N_5O_{15}$ or $C_{37}H_{59}N_{19}O_5$*	383.2293, 270.1457, 142.0862	4	n.a.			CAG, PDA	
121	874.5392	$[M+2H]^{2+}$	8.59	1.7	$C_{81}H_{142}N_{20}O_{22}$	n.a.±	3	Trichorzin MA-2	Peptaibol	*Trichoderma harzianum*	CAG, PDA	[31]
122	1136.6675	$[M+H]^+$	8.59	-0.4	$C_{52}H_{89}N_{13}O_{15}$ or $C_{50}H_{77}N_{27}O_5$*	Nf	4	n.a.			CAG, PDA	
123	623.4504	$[M+H]^+$	8.67	-0.8	$C_{33}H_{54}N_{10}O_2$*	324.2294, 215.1759, 211.1451, 183.1497	4	n.a.			CAG, PDA	
124	975.6241	$[M+H]^+$	8.67	-0.2	$C_{46}H_{70}N_{24}O$ or $C_{48}H_{82}N_{10}O_{11}$*	369.2148, 270.1460	4	n.a.			CAG, PDA	
125	874.5398	$[M+2H]^{2+}$	8.78	1.7	$C_{81}H_{142}N_{20}O_{22}$	n.a.±	3	Trichokindin Ia	Peptaibol	*Trichoderma harzianum*	CAG, PDA	[32]
126	1136.6686	$[M+H]^+$	8.78	0.6	$C_{52}H_{89}N_{13}O_{15}$ or $C_{50}H_{77}N_{27}O_5$*	697.3896, 484.2784, 399.2252, 286.1411, 268.1303 197.0926	4	n.a.			CAG, PDA	
127	623.4493	$[M+H]^+$	8.85	-1	$C_{33}H_{54}N_{10}O_2$*	324.2296, 215.1763, 211.1452, 183.1502	4	n.a.			CAG, PDA	
128	975.6257	$[M+H]^+$	8.85	0.1	$C_{51}H_{90}O_{17}$ or $C_{49}H_{78}N_{14}O_7$*	369.2148, 256.1304	4	n.a.			CAG, PDA	

No.	*m/z* value	Adduct	R_t (min)	ppm	Putative MF	Fragmentation pattern	IC	Putative identification	Chemical family	Biological origin	Medium	Ref
129	976.6094	$[M+H]^+$	8.96	-0.2	$C_{49}H_{77}N_{13}O_8$*	370.1984, 342.2027, 257.1147	4	n.a.			CAG, PDA	
130	623.4506	$[M+H]^+$	8.98	-0.5	$C_{33}H_{54}N_{10}O_2$*	324.2290, 296.1980, 215.1765, 211.1454, 183.1500	4	n.a.			CAG, PDA	
131	867.5361	$[M+2H]^{2+}$	8.98	n.a.±	n.a.±	n.a.±	4	n.a.			CAG, PDA	
132	892.5373	$[M+H+Na]^{2+}$	8.98	n.a.±	n.a.±	n.a.±	4	n.a.			CAG, PDA	
133	892.5372	$[M+H+Na]^{2+}$	9.04	n.a.±	n.a.±	n.a.±	4	n.a.			CAG, PDA	
134	909.527	$[M+2H]^{2+}$	9.04	-0.9	$C_{82}H_{140}N_{22}O_{24}$	n.a.±	3	Trichobrachin B-I	Peptaibol	*Trichoderma longibrachiatum*	CAG, PDA	[33]
135	989.6424	$[M+H]^+$	9.04	1.1	$C_{50}H_{80}N_{14}O_7$ or $C_{52}H_{92}O_{17}$*	383.2299, 270.1472	4	n.a.			CAG, PDA	
136	623.4505	$[M+H]^+$	9.04	-0.6	$C_{33}H_{54}N_{10}O_2$*	324.2296, 211.1455, 183.1503	4	n.a.			CAG, PDA	
137	623.4506	$[M+H]^+$	9.08	-0.3	$C_{33}H_{54}N_{10}O_2$*	324.2294, 211.1452, 183.1503	4	n.a.			CAG, PDA	
138	892.5372	$[M+H+Na]^{2+}$	9.08	n.a.±	n.a.±	n.a.±	4	n.a.			CAG, PDA	
139	989.6392	$[M+H]^+$	9.08	0.6	$C_{48}H_{88}N_6O_{15}$ or $C_{46}H_{76}N_{20}O_5$*	383.2303, 270.1464	4	n.a.			CAG, PDA	
140	892.5383	$[M+H+Na]^{2+}$	9.15	n.a.±	n.a.±	n.a.±	4	n.a.			CAG, PDA	
141	623.4499	$[M+H]^+$	9.21	0.5	$C_{32}H_{58}N_6O_6$*	324.2297, 215.1770, 211.1457, 183.1502	4	n.a.			CAG, PDA	
142	990.6248	$[M+H]^+$	9.21	0.9	$C_{47}H_{71}N_{23}O_2$*	384.2148, 356.2190, 271.1308, 142.0871	4	n.a.			CAG, PDA	
143	874.5402	$[M+2H]^{2+}$	9.25	1.7	$C_{81}H_{142}N_{20}O_{22}$	n.a.±	3	Trichokindin Ib	Peptaibol	*Trichoderma harzianum*	CAG, PDA	[32]
144	874.5392	$[M+2H]^{2+}$	9.38	1.7	$C_{81}H_{142}N_{20}O_{22}$	n.a.±	3	Trichokindin IIa	Peptaibol	*Trichoderma harzianum*	CAG, PDA	[32]
145	1136.6674	$[M+H]^+$	9.38	-0.4	$C_{52}H_{89}N_{13}O_{15}$ or $C_{50}H_{77}N_{27}O_5$*	697.3872, 484.2768, 399.2243, 286.1408, 268.1303, 197.0931	4	n.a.			CAG, PDA	

No.	*m/z* value	Adduct	R_t (min)	ppm	Putative MF	Fragmentation pattern	IC	Putative identification	Chemical family	Biological origin	Medium	Ref
146	1164.6991	$[M+H]^+$	9.41	-0.1	$C_{52}H_{81}N_{27}O_5$ or $C_{54}H_{93}N_{13}O_{15}$	498.2934, 399.2248, 286.1414, 268.1305, 197.0930	4	n.a.			CAG, PDA	
147	881.5459	$[M+2H]^{2+}$	9.5	0.4	$C_{82}H_{144}N_{20}O_{22}$	n.a.$^{\pm}$	3	Trichokindin IIb	Peptaibol	*Trichoderma harzianum*	CAG, PDA	[32]
148	844.5923	$[M+2H]^{2+}$	9.57	n.a.$^{\pm}$	n.a.$^{\pm}$	n.a.$^{\pm}$	4	n.a.			CAG, PDA	
149	873.55	$[M+2H]^{2+}$	9.57	2.2	$C_{82}H_{144}N_{20}O_{21}$	n.a.$^{\pm}$	3	Neoatroviridin B	Peptaibol	*Trichoderma atroviride*	CAG, PDA	[34]
150	881.5432	$[M+2H]^{2+}$	9.57	0.4	$C_{82}H_{144}N_{20}O_{22}$	n.a.$^{\pm}$	3	Trichokindin IIIa/b	Peptaibol	*Trichoderma harzianum*	CAG, PDA	[32]
151	881.547	$[M+2H]^{2+}$	9.61	0.4	$C_{82}H_{144}N_{20}O_{22}$	n.a.$^{\pm}$	3	Trichokindin IV	Peptaibol	*Trichoderma harzianum*	CAG, PDA	[32]
152	902.5486	$[M+2H]^{2+}$	9.61	n.a.$^{\pm}$	n.a.$^{\pm}$	n.a.$^{\pm}$	4	n.a.			CAG, PDA	
153	913.543	$[M+2Na]^{2+}$	9.61	n.a.$^{\pm}$	n.a.$^{\pm}$	n.a.$^{\pm}$	4	n.a.			CAG, PDA	
154	881.5477	$[M+2H]^{2+}$	9.74	0.4	$C_{82}H_{144}N_{20}O_{22}$	n.a.$^{\pm}$	3	Trichokindin Va/b	Peptaibol	*Trichoderma harzianum*	CAG, PDA	[32]
155	888.5443	$[M+2H]^{2+}$	9.99	-0.8	$C_{83}H_{146}N_{20}O_{22}$	n.a.$^{\pm}$	3	Trichokindin VI	Peptaibol	*Trichoderma harzianum*	CAG, PDA	[32]
156	891.5471	$[M+H+Na]^{2+}$	9.99	n.a.$^{\pm}$	$C_{83}H_{146}N_{20}O_{21}$	n.a.$^{\pm}$	3	Neoatroviridin D	Peptaibol	*Trichoderma atroviride*	CAG, PDA	[34]
157	888.555	$[M+2H]^{2+}$	10.14	-0.8	$C_{83}H_{146}N_{20}O_{22}$	n.a.$^{\pm}$	3	Trichokindin VII	Peptaibol	*Trichoderma harzianum*	CAG, PDA	[32]
158	916.5155	$[M+2H]^{2+}$	10.14	n.a.$^{\pm}$	n.a.$^{\pm}$	n.a.$^{\pm}$	4	n.a.			CAG, PDA	
159	481.2935	$[M+Na]^+$	10.33, 10.39$^{\Delta}$	1	$C_{28}H_{42}O_5$	423.2865, 355.2252	3	Ergokonin B	Ergosterol	*Trichoderma koningii*	CAG, PDA	[35]
160	873.5497	$[M+2H]^{2+}$	10.33	2.2	$C_{82}H_{144}N_{20}O_{21}$	n.a.$^{\pm}$	3	Neoatroviridin C	Peptaibol	*Trichoderma atroviride*	CAG, PDA	[34]
161	895.5413	$[M+2H]^{2+}$	10.39	n.a.$^{\pm}$	n.a.$^{\pm}$	n.a.$^{\pm}$	4	n.a.			CAG, PDA	

Table S9. Putatively identified compounds produced by *Fusarium* sp. extracts CHG38-CAG and CHG38-PDA. Putative annotations were based on the accurate mass, predicted putative molecular formulae (MF), the retention time (R_t), the fragmentation pattern and the biological origin. *MF with best ppm error; IC: Identification confidence level [3]; Nf: No fragmentation detected or below noise threshold ($5e^1$); Ref = reference(s).

No.	*m/z* value	Adduct	R_t (min)	ppm	Putative MF	Fragmentation pattern	IC	Putative identification	Chemical family	Biological origin	Medium	Ref
162	235.0614	$[M+H]^+$	2.97	3.4	$C_{12}H_{10}O_5$	217.0504, 193.0493, 189.0548, 175.0385, 161.0598, 151.0394, 149.0594, 125.0603, 111.0084	3	Diploquinone A	Naphthoquinone	*Diplodia mutila*	PDA	[36]
163	277.0715	$[M+H]^+$	3.09	1.1	$C_{14}H_{12}O_6$	259.0608, 235.0603, 231.0657, 217.0498, 193.0496, 191.0697, 123.0438	3	Norjavanicin	Naphthoquinone	*Fusarium* sp.	PDA	[37]
164	235.0977	$[M+H]^+$	3.52	3	$C_{13}H_{14}O_4$	217.0866, 191.0711, 176.0474, 163.0749, 151.0393, 135.0808	2	Aloesol	Chromone	*Fusarium* sp.	PDA	[38]
165	233.0820	$[M+H]^+$	3.63	2.6	$C_{13}H_{12}O_4$	217.0872, 191.0711, 151.0393	2	Macrocarpone C	Chromone	*Fusarium tricinctum*	PDA	[39]
166	279.0881	$[M+H]^+$	3.7	4.3	$C_{14}H_{14}O_6$	261.0784, 243.0665, 219.0664, 201.0530, 191.0708, 177.0186, 173.0605, 163.0770	3	(-)-Citreoisocoumarin	Isocoumarin	*Fusarium tricinctum*	PDA	[39]
167	191.0710	$[M+H]^+$	3.97	1	$C_{11}H_{10}O_3$	176.0488, 151.0390, 149.9310, 135.0444, 110.0086	4	n.a.			PDA	
168	359.1109	$[M+Na]^+$	4.37	0.6	$C_{17}H_{20}O_7$	324.5316, 322.0607, 291.0259, 271.0585, 253.8915, 252.3335	3	3-O-Ethyldihydrofusarubin A or B	Naphthoquinone	*Fusarium solani*	PDA	[40]
169	339.1803	$[M+H]^+$	4.58	-1.5	$C_{18}H_{26}O_6$	303.1549, 285.1486, 267.1371, 259.1721, 257.1524, 229.0869, 217.0846, 215.0682, 189.0564, 177.0592, 175.0392, 167.0347, 161.0622, 149.0613, 135.1176	2	2'-hydroxyzearalanol	Zearalenone (Macrolide)	*Penicillium* sp.	CAG	[41]
170	249.0768	$[M+H]^+$	5.46	2	$C_{13}H_{12}O_5$	217.0504, 192.0427, 153.0183	4	n.a.			PDA	
171	259.0615	$[M+H]^+$	5.66	3.5	$C_{14}H_{10}O_5$	244.0369, 231.0674, 213.0604, 191.0726	3	Huperxanthone B	Xanthone	*Aspergillus versicolor*	PDA	[42]
172	384.3954	$[M+H]^+$	5.96	0	$C_{23}H_{49}N_3O$	367.3683, 296.2959	4	n.a.			PDA	
173	629.3642	$[M+Na]^+$	6.23	0.6	$C_{34}H_{54}O_9$	557.3420, 387.2336	4	n.a.			CAG, PDA	
174	690.2125	$[M+H]^+$	6.56	1.6	$C_{40}H_{23}N_{11}O_2$	373.0741, 355.0630	4	n.a.			CAG	
175	412.4270	$[M+H]^+$	6.59	0.7	$C_{25}H_{53}N_3O$	324.3275	4	n.a.			PDA	
176	319.1553	$[M+H]^+$	6.63	2.5	$C_{18}H_{22}O_5$	301.1441, 283.1342, 265.1237, 255.1386, 241.0873, 231.0662, 229.0860, 227.0712, 217.0859, 215.0706, 213.0561, 205.0868,	2	Zearalenone	Zearalenone (Macrolide)	*Fusarium graminearum*	CAG	[43]

No.	*m/z* value	Adduct	R_t (min)	ppm	Putative MF	Fragmentation pattern	IC	Putative identification	Chemical family	Biological origin	Medium	Ref
						203.0716, 189.0562, 187.0764, 185.0606, 175.0762, 169.0662, 157.0653						
177	575.1199	$[M+H]^+$	7.32	1.6	$C_{30}H_{22}O_{12}$*	287.0563, 274.0464, 259.0613	4	n.a.			CAG, PDA	
178	273.0771	$[M+H]^+$	7.43	2.9	$C_{15}H_{12}O_5$	258.0524, 255.0665, 230.0581, 227.0702	3	Griseoxanthone C	Xanthone	*Fusarium equiseti*	PDA	[44]
179	303.1605	$[M-H_2O]^+$	7.47	3	$C_{18}H_{24}O_5$	285.1493, 229.0855, 215.0695, 205.0511, 191.0348, 163.0399	2	Zearalanone	Zearalenone (Macrolide)	*Fusarium* spp.	CAG	[45]
180	289.1786	$[M+Na]^+$	7.91	2.1	$C_{16}H_{26}O_3$	Nf	4	n.a.			PDA	
181	691.4647	$[M+H]^+$	8.95	0.1	$C_{37}H_{62}N_4O_8$*	659.4388, 428.3079, 377.3063, 359.2942, 331.2998, 313.2980, 303.2622, 263.2337, 235.2063, 232.1362, 220.1098, 181.0634, 172.1087, 164.0696, 155.0792, 147.0751, 130.0498, 121.1051	4	n.a.			CAG	
182	659.4385	$[M+H]^+$	9.07	0.2	$C_{36}H_{58}N_4O_7$	428.3166, 377.3065, 359.2950, 331.2991, 303.2691, 263.2368, 232.1301, 215.1033, 185.0930, 172.1086, 164.0710, 155.0815, 147.0771, 130.0506	3	Fusaristatin A	Cyclic lipopeptide	*Fusarium* sp.	CAG, PDA	[46]
183	695.3956	$[M+H]^+$	9.52	1.2	$C_{44}H_{54}O_7$	379.3360, 309.2571, 295.2407, 253.1958, 239.1786, 213.1648, 201.1647, 199.1482, 187.1492, 185.1322, 173.1342, 171.1174, 161.1311, 159.1167, 157.1026, 149.1328, 147.1171, 145.1016, 143.0850, 133.1014, 131.0849	4	n.a.			PDA	
184	437.3425	$[M+H]^+$	10.67	1.1	$C_{30}H_{44}O_2$	401.3213, 381.2794, 367.2639, 353.2500, 341.2487, 339.2319, 327.2321, 313.2170, 307.2427, 267.2122, 225.1645, 211.1495, 197.1337, 183.1170, 169.1021, 157.1024	4	n.a.			CAG, PDA	
185	737.4779	$[M-H_2O]^+$	10.82	-0.3	$C_{48}H_{66}O_7$*	701.4548, 593.3277, 575.3162, 567.3481, 441.2057, 381.2049, 363.1950, 313.1449, 295.1334, 293.1901, 275.1901, 267.1755, 249.1637, 225.1637, 209.1335, 201.0553, 199.0755, 195.1171, 183.1152	4	n.a.			CAG	
186	468.3607	$[M+H]^+$	11.44	0.8	$C_{31}H_{47}O_3$	437.3426, 393.2792, 365.2496, 352.2406, 339.2327, 337.2165, 319.2072, 307.2425, 293.2269, 265.1950, 251.1812, 249.1646, 237.1648, 235.1481, 223.1490, 211.1495,	4	n.a.			CAG	

No.	*m/z* value	Adduct	R_t (min)	ppm	Putative MF	Fragmentation pattern	IC	Putative identification	Chemical family	Biological origin	Medium	Ref
						209.1336, 196.1250, 183.1177, 181.1023, 169.1022						
187	793.5020	$[M+H]^+$	11.54	0.5	$C_{47}H_{64}N_6O_5$*	761.4775, 743.4667, 651.4027	4	n.a.			CAG	
188	755.4899	$[M+H]^+$	11.54	1.6	$C_{48}H_{66}O_7$	Nf	4	n.a.			CAG	
189	437.3420	$[M+H]^+$	11.59	0	$C_{30}H_{44}O_2$	419.3311, 401.3185, 381.2783, 357.1451, 335.2753, 313.2141, 299.2004, 275.1782, 259.1690, 223.1484, 211.1479, 195.1165, 183.1165, 169.1019, 159.1168, 145.0999	4	n.a.			PDA	
190	436.3340	$[M+H]^+$	11.68	-0.2	$C_{30}H_{43}O_2$	421.3112, 385.2896, 365.2484, 337.2177, 323.2018, 317.2274, 311.2021, 301.1965, 297.1866, 261.1650, 235.1489, 209.1336, 195.1180	4	n.a.			CAG, PDA	
191	437.3424	$[M+H]^+$	11.69	0.9	$C_{30}H_{44}O_2$	421.3102, 419.3308, 401.3206, 385.2891, 337.2167, 323.2014, 317.2267, 313.2159, 311.2007, 301.1959, 297.1853, 275.1794, 263.1789, 261.1641, 259.1678, 253.1947, 249.1632, 235.1484, 223.1482, 221.1327, 211.1483, 209.1327, 197.1323, 195.1172, 183.1167, 169.1006	4	n.a.			PDA	
192	753.4741	$[M+H]^+$	11.82	-0.4	$C_{49}H_{60}N_4O_3$*	735.4622, 656.3732, 638.3626, 623.3362, 605.3292, 587.3196, 565.3297, 523.2813, 521.2742, 509.2715, 497.2628, 495.2540, 481.2378, 477.2425, 469.2356, 467.2224, 463.2249, 313.1443, 295.1342, 221.1326, 201.0557, 199.0752, 195.1175	4	n.a.			CAG	
193	436.3335	$[M+H]^+$	11.93	-1.3	$C_{30}H_{43}O_2$	421.3098, 403.2995, 393.2770, 385.2880, 340.2391, 325.2155, 323.2359, 321.2568, 319.2407, 307.2062, 293.2261, 279.2098, 265.1939, 251.1783, 249.1631, 237.1637, 235.1479, 225.1630, 223.1380, 211.1479, 208.1323, 197.1318, 195.1164, 184.1246, 169.1005, 155.0851	4	n.a.			CAG, PDA	

Table S10. Putatively identified compounds produced by *Penicillium* sp. extracts CKG23-CAG and CKG23-PDA. Putative annotations were based on the accurate mass, the predicted putative molecular formulae (MF), the retention time (R_t), the fragmentation pattern and the biological origin. $^{\Delta}$Different isomers with same *m/z* value and molecular formula, which cannot be differentiated based on MS/MS data; *MF with best ppm error displayed; IC: Identification confidence level [3]; Nf: No fragmentation detected or below noise threshold ($5e^1$); Ref = reference(s).

No.	*m/z* value	Adduct	R_t (min)	ppm	Putative MF	Fragmentation pattern	IC	Putative identification	Chemical family	Biological origin	Medium	Ref
194	245.082	$[M+H]^+$	1.24	2.4	$C_{14}H_{12}O_4$	227.0711, 217.0858, 209.0590, 199.0762, 189.0916, 185.0596, 181.0651, 175.0771, 173.0607, 171.0809, 161.0609, 157.0655, 153.0705, 151.0388, 147.0443, 143.0853, 135.0447, 123.0446, 107.0495	3	3-methylbisnoryangonin	Styrylpyrone	*Penicillium glabrum*	CAG, PDA	[47]
195	157.0059	$[M+H]^+$	2.14	1.9	$C_7H_5O_2Cl$	129.0107, 121.0266, 101.0160, 94.0412	4	n.a.			CAG	
196	233.082	$[M+H]^+$	2.84	2.6	$C_{13}H_{12}O_4$	194.9539, 191.0710, 187.0742, 173.0598, 149.0604, 147.0451, 123.0438, 121.0669, 85.0288	4	n.a.			PDA	
197	325.1292	$[M+H]^+$	3.46	1.5	$C_{16}H_{20}O_7$	289.1072, 247.0972, 233.0819, 231.0664, 227.1076, 213.0559, 191.0341, 189.0549, 183.0296, 165.0552	3	11,12-Dihydroxycurvularin	Zearalenone (Macrolide)	*Penicillium citreo-viride*	PDA	[48]
198	327.1235	$[M+H]^+$	3.9	0.9	$C_{19}H_{18}O_5$	309.1131, 294.0894, 285.0765	3	1,7-Dihydroxy-2-methoxy-3-prenylxanthone	Xanthone	*Phomopsis* sp.	CAG	[49]
199	309.1345	$[M+H]^+$	4	2.3	$C_{16}H_{20}O_6$	273.1129, 255.1038, 231.1031, 229.1228, 215.0716, 213.0914, 201.0553, 189.0924, 187.0768, 177.0551, 175.0400, 173.0602, 161.0605, 149.0605	3	(3S,7S)-7-hydroxyresorcylide	Zearalenone (Macrolide)	*Penicillium* sp.	PDA	[50]
200	273.0409	$[M+H]^+$	4.12	3.7	$C_{14}H_8O_6$	245.0457, 227.0340, 217.0508, 199.0397	3	2,8-dihydroxy-9-oxo-9H-xanthene-6-carboxylic acid	Xanthone	*Arthrinium arundinis*	PDA	[51]
201	399.1813	$[M+H]^+$	4.55, 4.87$^{\Delta}$	1.3	$C_{23}H_{26}O_6$	381.1706, 363.1587, 355.1514, 352.1281, 348.1369, 339.1246, 327.1208, 311.0899	3	Seco-penicitrinol A	Xanthone	*Penicillium citrinum*	CAG	[52]
202	289.0717	$[M+H]^+$	4.57	1.7	$C_{15}H_{12}O_6$	274.0483, 270.0526, 246.0534, 243.0657, 200.0488	3	Drimiopsin I	Xanthone	*Penicillium* sp.	PDA	[53]
203	511.2927	$[M+H]^+$	4.96	1.4	$C_{28}H_{38}N_4O_5$	265.1557, 247.1459, 219.1503, 199.1437, 171.1505, 166.0866, 120.0811, 72.0825	2	Bilaid A	Tetrapeptide	*Penicillium* sp.	PDA	[54]
204	447.2936	$[M+Na]^+$	5.22	0.4	$C_{21}H_{44}O_8$	Nf	4	n.a.			PDA	
205	309.1343	$[M+H]^+$	5.25	1.6	$C_{16}H_{20}O_6$	291.1229, 273.1129, 255.1022, 245.1183, 231.0663, 227.1073, 217.0497, 213.0556, 207.0497, 195.0292, 193.0501, 191.0343,	3	(3S,7R)-7-hydroxyresorcylide	Zearalenone (Macrolide)	*Penicillium* sp.	PDA	[50]

No.	*m/z* value	Adduct	R_t (min)	ppm	Putative MF	Fragmentation pattern	IC	Putative identification	Chemical family	Biological origin	Medium	Ref
						183.0292, 181.0136, 177.0192, 167.0341, 165.0184, 159.0445, 99.0812, 81.0706						
206	457.2601	$[M+H]^+$	5.47	-0.7	$C_{28}H_{32}N_4O_2$	440.2370, 439.2495, 399.2179, 385.2024, 382.1931, 381.1940, 368.1750, 340.1801, 326.1655, 323.1541, 309.1378, 299.1517, 297.1387, 255.1489, 238.1450, 210.1285, 198.1156, 197.1081, 185.1075, 183.0918, 181.0893, 168.0813, 159.0927	2	Communesin A	Indole alkaloid	*Penicillium* sp.	CAG	[55]
207	427.1757	$[M+H]^+$	5.47	0	$C_{24}H_{26}O_7$	381.1703, 363.1595, 354.1459, 348.1355, 339.1234, 326.1150, 321.1122, 311.0921, 308.1045, 297.0759	4	n.a.			CAG	
208	293.1393	$[M+H]^+$	5.97	1.4	$C_{16}H_{20}O_5$	275.1283, 257.1166, 239.1059, 231.1375, 229.1238, 215.0709, 205.0494, 201.0546, 189.0547, 187.0414, 179.0341, 177.0546, 175.0388, 173.0599, 163.0749, 161.0593, 151.0390, 149.0599, 99.0811, 81.0702	3	Dihydroresorcylide	Zearalenone (Macrolide)	*Penicillium brocae*	CAG, PDA	[56]
209	529.2708	$[M+H]^+$	6	1.1	$C_{32}H_{36}N_2O_5$	331.1811, 200.1068, 185.0722, 130.0658	3	Chaetoglobosin D	Cytochalasan alkaloid	*Penicillium expansum*	CAG	[57]
210	509.2773	$[M+H]^+$	6.11	-0.8	$C_{29}H_{32}N_8O$*	311.1388, 283.1449, 263.1399, 247.1446, 235.1447, 219.1502, 199.1440, 171.1498, 136.0762, 120.0815	4	n.a.			PDA	
211	357.1341	$[M+H]^+$	6.22	0.8	$C_{20}H_{20}O_6$	342.1103, 327.0872, 315.0868, 313.0717, 301.0714, 286.0481, 257.0454, 229.0506	4	n.a.			PDA	
212	529.27	$[M+H]^+$	6.29	-0.4	$C_{32}H_{36}N_2O_5$	200.1071, 198.0916, 185.0718, 174.0925, 157.1017, 130.0657	2	Chaetoglobosin A	Cytochalasan alkaloid	*Penicillium chrysogenum*	CAG	[58]
213	307.1551	$[M+H]^+$	6.38	2	$C_{17}H_{22}O_5$	265.1435, 201.1277, 155.0864, 135.0807, 133.1008, 131.0864, 105.0695	3	Expansolide A or B	Sesquiterpenoid	*Penicillium expansum*	CAG	[59]
214	707.2095	$[M+H]^+$	6.42	-1	$C_{36}H_{30}N_6O_{10}$ or $C_{34}H_{18}N_{20}$*	668.0617, 602.0138, 584.0034, 573.0614, 365.0998, 296.0298, 275.0551	4	n.a.			PDA	
215	313.1084	$[M+H]^+$	6.64	2.6	$C_{18}H_{16}O_5$	297.0768, 271.0612, 257.0456, 229.0503	4				PDA	
216	289.0716	$[M+H]^+$	6.72	1.4	$C_{15}H_{12}O_6$	274.0482, 270.0525, 246.0518, 243.0660, 232.0370, 228.0396, 200.0479	3	Drimiopsin H	Xanthone	*Penicillium* sp.	PDA	[53]
217	305.1296	$[M+H]^+$	6.72	2	$C_{19}H_{16}N_2O_2$	277.1343, 234.1274, 220.1120, 187.0869, 132.0810	4	n.a.			CAG	
218	339.0871	$[M+H]^+$	6.85	0.6	$C_{19}H_{14}O_6$	324.0636, 321.0758, 311.0922, 296.0687, 293.0815, 283.0966, 269.0456, 265.0869, 249.0918	4	n.a.			PDA	

No.	*m/z* value	Adduct	R_t (min)	ppm	Putative MF	Fragmentation pattern	IC	Putative identification	Chemical family	Biological origin	Medium	Ref
219	509.2922	$[M+H]^+$	7.04	1	$C_{32}H_{36}N_4O_2$	491.2809, 451.2503, 437.2342, 381.1972, 367.1808, 357.2074, 343.1924, 340.1916, 326.1661, 323.1545, 309.1391, 255.1501, 253.1346, 197.1083, 185.1085, 183.0922, 159.0922, 95.0501	2	Communesin B	Indole alkaloid	*Penicillium* sp.	CAG	[55]
220	357.1341	$[M+H]^+$	7.05	0.8	$C_{20}H_{20}O_6$	341.1023, 327.0877, 313.1076, 311.0921, 301.0721, 286.0482, 273.0771, 258.0528, 247.0608, 230.0600	4	n.a.			PDA	
221	359.1135	$[M+H]^+$	7.22, 7.71$^\Delta$	1.1	$C_{19}H_{18}O_7$	341.1030, 326.0796, 323.0922, 313.1078, 302.0749, 271.0614, 257.0455	3	Penixanthone D	Xanthone	*Penicillium* sp.	PDA	[60]
222	487.27	$[M+H]^+$	7.22	0.8	$C_{28}H_{38}O_7$	395.2227, 377.2127, 367.2277, 349.2178, 243.1752, 225.1640, 215.1806, 185.1337, 183.1183, 175.1487, 171.1180, 161.1338, 151.0396	2	Andrastin A	Meroterpenoid	*Penicillium* sp.	CAG	[61]
223	319.1455	$[M+H]^+$	7.28	2.5	$C_{20}H_{18}N_2O_2$	291.1506, 234.1290, 201.1029, 188.0718, 132.0817, 91.0551	4	n.a.			CAG	
224	307.1552	$[M+H]^+$	7.42	2.1	$C_{17}H_{22}O_5$	289.1434, 271.1339, 243.1392, 229.0860, 193.0503, 191.0710, 189.0561, 175.0766, 165.0543, 163.0751	3	a: 5-oxolasiodiplodin, b: 7-oxolasiodiplodin	Zearalenone (Macrolide)	*a: Lasiodiplodia theobromae, b: Lasiodiplodia sp.*	PDA	a: [62], b: [63]
225	355.1188	$[M+H]^+$	7.54	1.7	$C_{20}H_{18}O_6$*	339.0874, 325.0719, 311.0926	4	n.a.			PDA	
226	357.1346	$[M+H]^+$	8	2.2	$C_{20}H_{20}O_6$	342.1128, 327.0954, 315.0874, 313.0724, 301.0730, 286.0486, 281.0819, 273.0769, 258.0534, 255.0661, 247.0605	4	n.a.			PDA	
227	711.2451	$[M+H]^+$	8.12	-0.6	$C_{41}H_{54}N_4O_8$*	679.2186, 636.2005, 356.1266, 341.1040, 327.1239, 232.0929	4	n.a.			PDA	
228	367.2253	$[M+Na]^+$	8.13	1.1	$C_{22}H_{32}O_3$	320.1843, 252.0905, 242.1019, 234.1131, 224.0923, 198.1151, 149.0231, 138.0189	4	n.a.			CAG	
229	389.2327	$[M+H]^+$	8.39	-0.3	$C_{23}H_{32}O_5$	Nf	4	n.a.			CAG	
230	865.5916	$[M+H]^+$	8.8	0.1	$C_{47}H_{76}N_8O_7$*	594.4034, 526.3393, 481.3192, 413.2555, 408.3224, 368.2338, 340.2582, 300.1718, 295.2392, 272.1769, 267.2441, 227.1761, 199.1817, 159.0919	4	n.a.			CAG	
231	500.3957	$[M+H]^+$	8.95	1.2	$C_{28}H_{53}NO_6$*	482.3847, 236.1509, 144.1026	4	n.a.			PDA	
232	371.1132	$[M+H]^+$	9.49	0.3	$C_{20}H_{18}O_7$	339.0872, 329.1029, 311.0927, 296.0686, 287.0559, 283.0973, 273.0402, 269.0455, 265.0868, 255.0296, 241.0500	3	Chaetoxanthone A	Xanthone	*Chaetomium* sp.	PDA	[64]

No.	*m/z* value	Adduct	R_t (min)	ppm	Putative MF	Fragmentation pattern	IC	Putative identification	Chemical family	Biological origin	Medium	Ref
233	467.0751	$[M+H]^+$	9.49	-0.4	$C_{24}H_6N_{10}O_2$*	426.0487, 423.0406, 421.0434, 412.9966, 405.0294, 403.0327, 390.0050, 388.0096, 385.0206, 371.9950, 369.9992	4	n.a.			PDA	
234	279.233	$[M+H]^+$	9.66	2.1	$C_{18}H_{30}O_2$	261.2221, 223.1700, 209.1532, 201.0458, 195.1395, 191.1436, 187.1490, 177.1271, 173.1328, 163.1481, 159.1174, 151.1479, 149.1330, 147.1175, 145.1004, 137.1334, 135.1168, 131.0853, 123.1168, 121.1018, 109.1015, 107.0859, 95.0860, 93.0696, 81.0707	4	n.a.			PDA	
235	279.2333	$[M+H]^+$	9.74	3.2	$C_{18}H_{30}O_2$	261.2222, 209.1537, 201.0462, 195.1386, 191.1434, 187.1488, 177.1286, 173.1329, 163.1490, 159.1176, 151.1487, 149.1335, 147.1167, 145.1022, 137.1336, 135.1173, 133.1024, 131.0863, 123.1177, 121.1020, 109.1017, 107.0863, 95.0861, 93.0698, 81.0708, 79.0556	4	n.a.			PDA	
236	343.1188	$[M+H]^+$	9.82	1.7	$C_{19}H_{18}O_6$	327.0860, 313.0720, 301.0718, 287.0565, 275.0558, 259.0612, 233.0453, 213.0556	3	Umbilicaxanthone A	Xanthone	*Umbilicaria proboscidea*	PDA	[65]
237	843.3354	$[M+H]^+$	9.82	0	$C_{46}H_{46}N_6O_{10}$ or $C_{44}H_{34}N_{20}$*	364.0936	4	n.a.			PDA	
238	714.4153	$[M+H]^+$	10.44	-0.7	$C_{47}H_{55}NO_5$*	696.4050, 571.2720	4	n.a.			PDA	
239	850.4537	$[M+H]^+$	10.93	0.8	$C_{49}H_{51}N_{15}$ or $C_{51}H_{63}NO_{10}$*	377.3211, 341.1031, 326.0794, 313.1082	4	n.a.			PDA	

References

1. Wang, M.; Carver, J.J.; Phelan, V.V.; Sanchez, L.M.; Garg, N.; Peng, Y.; Nguyen, D.D.; Watrous, J.; Kapono, C.A.; Luzzatto-Knaan, T.; et al. Sharing and community curation of mass spectrometry data with Global Natural Products Social Molecular Networking. *Nat. Biotechnol.* **2016**, *34*, 828-837, doi:10.1038/nbt.3597.
2. Altschul, S.F.; Gish, W.; Miller, W.; Myers, E.W.; Lipman, D.J. Basic local alignment search tool. *J. Mol. Biol.* **1990**, *215*, 403-410, doi:10.1016/S0022-2836(05)80360-2.
3. Sumner, L.W.; Amberg, A.; Barrett, D.; Beale, M.H.; Beger, R.; Daykin, C.A.; Fan, T.W.; Fiehn, O.; Goodacre, R.; Griffin, J.L.; et al. Proposed minimum reporting standards for chemical analysis Chemical Analysis Working Group (CAWG) Metabolomics Standards Initiative (MSI). *Metabolomics* **2007**, *3*, 211-221, doi:10.1007/s11306-007-0082-2.
4. Drautz, H.; Zähner, H.; Kupfer, E.; Keller-Schierlein, W. 164. Stoffwechselprodukte von Mikroorganismen, 205. Mitteilung. Isolierung und Struktur von Streptazolin. *Helv. Chim. Acta* **1981**, *64*, 1752-1765, doi:10.1002/hlca.19810640605.
5. Puder, C.; Loya, S.; Hizi, A.; Zeeck, A. New co-metabolites of the streptazolin pathway. *J. Nat. Prod.* **2001**, *64*, 42-45, doi:10.1021/np000377i.
6. Zhang, C.; Ondeyka, J.; Guan, Z.; Dietrich, L.; Burgess, B.; Wang, J.; Singh, S.B. Isolation, structure and biological activities of platensimycin B4 from *Streptomyces platensis*. *J. Antibiot.* **2009**, *62*, 699-702, doi:10.1038/ja.2009.106.
7. Paulus, C.; Rebets, Y.; Zapp, J.; Ruckert, C.; Kalinowski, J.; Luzhetskyy, A. New alpiniamides from *Streptomyces* sp. IB2014/011-12 assembled by an unusual hybrid non-ribosomal peptide synthetase Trans-AT polyketide synthase enzyme. *Front. Microbiol.* **2018**, *9*, 1959, doi:10.3389/fmicb.2018.01959.
8. Fleck, W.F.; Ritzau, M.; Heinze, S.; Gräfe, U. Isolation of dimeric nonactic acid from the nonactin-producing *Streptomyces* spec. JA 5909-1. *J. Basic Microbiol.* **1996**, *36*, 235-238, doi:10.1002/jobm.3620360405.
9. Schumacher, R.W.; Talmage, S.C.; Miller, S.A.; Sarris, K.E.; Davidson, B.S.; Goldberg, A. Isolation and structure determination of an antimicrobial ester from a marine sediment-derived bacterium. *J. Nat. Prod.* **2003**, *66*, 1291-1293, doi:10.1021/np020594e.
10. Huang, H.; Lan, X.; Wang, Y.; Tian, L.; Fang, Y.; Zhang, L.; Zhang, K.; Zheng, X. New bioactive derivatives of nonactic acid from the marine *Streptomyces griseus* derived from the plant *Salicornia* sp. *Phytochem. Lett.* **2015**, *12*, 190-195, doi:10.1016/j.phytol.2015.04.001.
11. Jeong, S.Y.; Shin, H.J.; Kim, T.S.; Lee, H.S.; Park, S.K.; Kim, H.M. Streptokordin, a new cytotoxic compound of the methylpyridine class from a marine-derived *Streptomyces* sp. KORDI-3238. *J. Antibiot.* **2006**, *59*, 234-240, doi:10.1038/ja.2006.33.
12. Charan, R.D.; Schlingmann, G.; Janso, J.; Bernan, V.; Feng, X.; Carter, G.T. Diazepinomicin, a new antimicrobial alkaloid from a marine *Micromonospora* sp. *J. Nat. Prod.* **2004**, *67*, 1431-1433, doi:10.1021/np040042r.
13. Maskey, R.P.; Grun-Wollny, I.; Laatsch, H. Isolation and structure elucidation of diazaquinomycin C from a terrestrial *Streptomyces* sp. and confirmation of the akashin structure. *Nat. Prod. Res.* **2005**, *19*, 137-142, doi:10.1080/14786410410001704741.
14. McBrien, K.D.; Berry, R.L.; Lowe, S.E.; Neddermann, K.M.; Bursuker, I.; Huang, S.; Klohr, S.E.; Leet, J.E. Rakicidins, new cytotoxic lipopeptides from *Micromonospora* sp. fermentation, isolation and characterization. *J. Antibiot.* **1995**, *48*, 1446-1452, doi:10.7164/antibiotics.48.1446.
15. Oku, N.; Matoba, S.; Yamazaki, Y.M.; Shimasaki, R.; Miyanaga, S.; Igarashi, Y. Complete stereochemistry and preliminary structure-activity relationship of rakicidin A, a hypoxia-selective cytotoxin from *Micromonospora* sp. *J. Nat. Prod.* **2014**, *77*, 2561-2565, doi:10.1021/np500276c.

16. Itoh, J.; Omoto, S.; Shomura, T.; Nishizawa, N.; Miyado, S.; Yuda, Y.; Shibata, U.; Inouye, S. Amicoumacin-A, a new antibiotic with strong antiinflammatory and antiulcer activity. *J. Antibiot.* **1981**, *34*, 611-613, doi:10.7164/antibiotics.34.611.
17. Bai, J.; Liu, D.; Yu, S.; Proksch, P.; Lin, W. Amicoumacins from the marine-derived bacterium *Bacillus* sp. with the inhibition of NO production. *Tetrahedron Lett.* **2014**, *55*, 6286-6291, doi:10.1016/j.tetlet.2014.09.100.
18. Shimojima, Y.; Hayashi, H.; Ooka, T.; Shibukawa, M. Production, isolation and pharmacological studies of AI-77s. *Agr. Biol. Chem.* **2014**, *46*, 1823-1829, doi:10.1080/00021369.1982.10865335.
19. Xia, M.; Liu, S.W.; Zhou, Z.H.; Tuo, L.; Ling, B.; Guo, L.; Dong, Y.P.; Wang, F.F.; Jiang, Z.K.; Li, X.J.; et al. Studies on amicoumacin group antibiotics produced by *Bacillus* strain XZ7. *Chin. J. Antibiot.* **2014**, *39*, 801-807.
20. Mhammedi, A.; Peypoux, F.; Besson, F.; Michel, G. Bacillomycin F, a new antibiotic of iturin group: isolation and characterization. *J. Antibiot.* **1982**, *35*, 306-311, doi:10.7164/antibiotics.35.306.
21. Peypoux, F.; Marion, D.; Maget-Dana, R.; Ptak, M.; Das, B.C.; Michel, G. Structure of bacillomycin F, a new peptidolipid antibiotic of the iturin group. *Eur. J. Biochem.* **1985**, *153*, 335-340, doi:10.1111/j.1432-1033.1985.tb09307.x.
22. Konda, Y.; Nakagawa, A.; Harigaya, Y.; Onda, M.; Masuma, R.; Omura, S. Aurantinin B, a new antimicrobial antibiotic from bacterial origin. *J. Antibiot.* **1988**, *41*, 268-270, doi:10.7164/antibiotics.41.268.
23. Kimura, K.; Nakayama, S.; Nakamura, J.; Takada, T.; Yoshihama, M.; Esumi, Y.; Itoh, Y.; Uramoto, M. SNA-60-367, new peptide enzyme inhibitors against aromatase. *J. Antibiot.* **1997**, *50*, 529-531, doi:10.7164/antibiotics.50.529.
24. Zhuravleva, O.I.; Afiyatullov, S.S.; Ermakova, S.P.; Nedashkovskaya, O.I.; Dmitrenok, P.S.; Denisenko, V.A.; Kuznetsova, T.A. New C14-surfactin methyl ester from the marine bacterium *Bacillus pumilus* KMM 456. *Russ. Chem. Bull.* **2011**, *59*, 2137-2142, doi:10.1007/s11172-010-0369-8.
25. Liu, X.Y.; Yang, S.Z.; Mu, B.Z. Isolation and characterization of a C12-lipopeptide produced by *Bacillus subtilis* HSO 121. *J. Pept. Sci.* **2008**, *14*, 864-875, doi:10.1002/psc.1017.
26. Kalinovskaya, N.I.; Kuznetsova, T.A.; Ivanova, E.P.; Romanenko, L.A.; Voinov, V.G.; Huth, F.; Laatsch, H. Characterization of surfactin-like cyclic depsipeptides synthesized by *Bacillus pumilus* from ascidian *Halocynthia aurantium*. *Mar. Biotechnol.* **2002**, *4*, 179-188, doi:10.1007/s1012601-0084-4.
27. Zhang, P.; Deng, Y.; Lin, X.; Chen, B.; Li, J.; Liu, H.; Chen, S.; Liu, L. Anti-inflammatory mono- and dimeric sorbicillinoids from the marine-derived fungus *Trichoderma reesei* 4670. *J. Nat. Prod.* **2019**, *82*, 947-957, doi:10.1021/acs.jnatprod.8b01029.
28. Jiao, W.H.; Khalil, Z.; Dewapriya, P.; Salim, A.A.; Lin, H.W.; Capon, R.J. Trichodermides A-E: New peptaibols isolated from the Australian termite nest-ferived fungus *Trichoderma virens* CMB-TN16. *J. Nat. Prod.* **2018**, *81*, 976-984, doi:10.1021/acs.jnatprod.7b01072.
29. Mukherjee, P.K.; Wiest, A.; Ruiz, N.; Keightley, A.; Moran-Diez, M.E.; McCluskey, K.; Pouchus, Y.F.; Kenerley, C.M. Two classes of new peptaibols are synthesized by a single non-ribosomal peptide synthetase of *Trichoderma virens*. *J. Biol. Chem.* **2011**, *286*, 4544-4554, doi:10.1074/jbc.M110.159723.
30. Iida, A.; Sanekata, M.; Wada, S.; Fujita, T.; Tanaka, H.; Enoki, A.; Fuse, G.; Kanai, M.; Asami, K. Fungal metabolites. XVIII. New membrane-modifying peptides, trichorozins I-IV, from the fungus *Trichoderma harzianum*. *Chem. Pharm. Bull.* **1995**, *43*, 392-397, doi:10.1248/cpb.43.392.
31. Goulard, C.; Hlimi, S.; Rebuffat, S.; Bodo, B. Trichorzins HA and MA, antibiotic peptides from *Trichoderma harzianum*. I. Fermentation, isolation and biological properties. *J. Antibiot.* **1995**, *48*, 1248-1253, doi:10.7164/antibiotics.48.1248.
32. Iida, A.; Sanekata, M.; Fujita, T.; Tanaka, H.; Enoki, A.; Fuse, G.; Kanai, M.; Rudewicz, P.J.; Tachikawa, E. Fungal metabolites. XVI. Structures of new peptaibols, trichokindins I-VII, from

the fungus *Trichoderma harzianum*. *Chem. Pharm. Bull.* **1994**, *42*, 1070-1075, doi:10.1248/cpb.42.1070.
33. Mohamed-Benkada, M.; Montagu, M.; Biard, J.F.; Mondeguer, F.; Verite, P.; Dalgalarrondo, M.; Bissett, J.; Pouchus, Y.F. New short peptaibols from a marine *Trichoderma* strain. *Rapid Commun. Mass Spectrom.* **2006**, *20*, 1176-1180, doi:10.1002/rcm.2430.
34. Oh, S.U.; Yun, B.S.; Lee, S.J.; Kim, J.H.; Yoo, I.D. Atroviridins A-C and neoatroviridins A-D, novel peptaibol antibiotics produced by *Trichoderma atroviride* F80317. I. Taxonomy, fermentation, isolation and biological activities. *J. Antibiot.* **2002**, *55*, 557-564, doi:10.7164/antibiotics.55.557.
35. Augustiniak, H.; Forche, E.; Reichenbach, H.; Wray, V.; Gräfe, U.; Höfle, G. Isolierung und Strukturaufklarung von Ergokonin A und B; zwei neue antifungische Sterol-Antibiotika aus *Trichoderma koningii*. *Liebigs Ann. Chem.* **1991**, *1991*, 361-366, doi:10.1002/jlac.199119910163.
36. Reveglia, P.; Savocchia, S.; Billones-Baaijens, R.; Masi, M.; Cimmino, A.; Evidente, A. Diploquinones A and B, two new phytotoxic tetrasubstituted 1,4-naphthoquinones from *Diplodia mutila*, a causal agent of grapevine trunk disease. *J. Agr. Food Chem.* **2018**, *66*, 11968-11973, doi:10.1021/acs.jafc.8b05004.
37. Chilton, W.S. Isolation and structure of norjavanicin. *J. Org. Chem.* **1968**, *33*, 4299-4300, doi:10.1021/jo01275a074.
38. Trisuwan, K.; Khamthong, N.; Rukachaisirikul, V.; Phongpaichit, S.; Preedanon, S.; Sakayaroj, J. Anthraquinone, cyclopentanone, and naphthoquinone derivatives from the sea fan-derived fungi *Fusarium* spp. PSU-F14 and PSU-F135. *J. Nat. Prod.* **2010**, *73*, 1507-1511, doi:10.1021/np100282k.
39. Ola, A.R.; Thomy, D.; Lai, D.; Brotz-Oesterhelt, H.; Proksch, P. Inducing secondary metabolite production by the endophytic fungus *Fusarium tricinctum* through coculture with *Bacillus subtilis*. *J. Nat. Prod.* **2013**, *76*, 2094-2099, doi:10.1021/np400589h.
40. Kurobane, I.; Vining, L.C.; McInnes, A.G.; Gerber, N.N. Metabolites of *Fusarium solani* related to dihydrofusarubin. *J. Antibiot.* **1980**, *33*, 1376-1379, doi:10.7164/antibiotics.33.1376.
41. Yang, X.; Khong, T.T.; Chen, L.; Choi, H.D.; Kang, J.S.; Son, B.W. 8'-hydroxyzearalanone and 2'-hydroxyzearalanol: Resorcyclic acid lactone derivatives from the marine-derived fungus *Penicillium* sp. *Chem. Pharm. Bull.* **2008**, *56*, 1355-1356, doi:10.1248/cpb.56.1355.
42. Ma, T.-T.; Shan, W.-G.; Ying, Y.-M.; Ma, L.-F.; Liu, W.-H.; Zhan, Z.-J. Xanthones with α-glucosidase inhibitory activities from *Aspergillus versicolor*, a fungal endophyte of *Huperzia serrata*. *Helv. Chim. Acta* **2015**, *98*, 148-152, doi:10.1002/hlca.201400165.
43. Stob, M.; Baldwin, R.S.; Tuite, J.; Andrews, F.N.; Gillette, K.G. Isolation of an anabolic, uterotrophic compound from corn infected with *Gibberella zeae*. *Nature* **1962**, *196*, 1318, doi:10.1038/1961318a0.
44. Hawas, U.W.; Farrag, A.R.H.; Ahmed, E.F.; Abou El-Kassem, L.T. Cytotoxic effect of *Fusarium equiseti* fungus metabolites against N-Nitrosodiethylamine- and CCL4-induced hepatocarcinogenesis in rats. *Pharm. Chem. J.* **2018**, *52*, 326-333, doi:10.1007/s11094-018-1816-3.
45. Richardson, K.E.; Hagler, W.M.; Mirocha, C.J. Production of zearalenone, α- and β-zearalenol, and α- and β-zearalanol by *Fusarium* spp. in rice culture. *J. Agr. Food Chem.* **1985**, *33*, 862-866, doi:10.1021/jf00065a024.
46. Shiono, Y.; Tsuchinari, M.; Shimanuki, K.; Miyajima, T.; Murayama, T.; Koseki, T.; Laatsch, H.; Funakoshi, T.; Takanami, K.; Suzuki, K. Fusaristatins A and B, two new cyclic lipopeptides from an endophytic *Fusarium* sp. *J. Antibiot.* **2007**, *60*, 309-316, doi:10.1038/ja.2007.39.
47. Hammerschmidt, L.; Wray, V.; Lin, W.; Kamilova, E.; Proksch, P.; Aly, A.H. New styrylpyrones from the fungal endophyte *Penicillium glabrum* isolated from *Punica granatum*. *Phytochem. Lett.* **2012**, *5*, 600-603, doi:10.1016/j.phytol.2012.06.003.
48. Lai, S.; Shizuri, Y.; Yamamura, S.; Kawai, K.; Furukawa, H. New curvularin-type metabolites from the hybrid strain ME 0005 derived from *Penicillium citreo-viride* B. IFO 4692 and 6200. *Bull. Chem. Soc. Jpn.* **1991**, *64*, 1048-1050, doi:10.1246/bcsj.64.1048.

49. Huang, Z.; Yang, R.; Yin, X.; She, Z.; Lin, Y. Structure elucidation and NMR assignments for two xanthone derivatives from a mangrove endophytic fungus (No. ZH19). *Magn. Reson. Chem.* **2010**, *48*, 80-82, doi:10.1002/mrc.2539.
50. Li, F.; Sun, W.; Zhang, S.; Gao, W.; Lin, S.; Yang, B.; Chai, C.; Li, H.; Wang, J.; Hu, Z.; et al. New cyclopiane diterpenes with anti-inflammatory activity from the sea sediment-derived fungus *Penicillium* sp. TJ403-2. *Chin. Chem. Lett.* **2020**, *31*, 197-201, doi:10.1016/j.cclet.2019.04.036.
51. Liao, Z.-J.; Tian, W.-J.; Liu, X.-X.; Jiang, X.; Wu, Y.; Lin, T.; Chen, H.-F. A new xanthone from an endophytic fungus of *Anoectochilus roxburghii*. *Chem. Nat. Compd.* **2018**, *54*, 267-269, doi:10.1007/s10600-018-2320-4.
52. Yang, S.-Q.; Li, X.-M.; Li, X.; Li, H.-L.; Meng, L.-H.; Wang, B.-G. New citrinin analogues produced by coculture of the marine algal-derived endophytic fungal strains *Aspergillus sydowii* EN-534 and *Penicillium citrinum* EN-535. *Phytochem. Lett.* **2018**, *25*, 191-195, doi:10.1016/j.phytol.2018.04.023.
53. Zhuang, Y.-B.; Yin, H.; Zhang, X.-W.; Zhou, W.; Liu, T. Three new xanthones from the fungus *Penicillium* sp. NH-7-1. *Helv. Chim. Acta* **2015**, *98*, 699-703, doi:10.1002/hlca.201400296.
54. Dekan, Z.; Sianati, S.; Yousuf, A.; Sutcliffe, K.J.; Gillis, A.; Mallet, C.; Singh, P.; Jin, A.H.; Wang, A.M.; Mohammadi, S.A.; et al. A tetrapeptide class of biased analgesics from an Australian fungus targets the micro-opioid receptor. *Proc. Natl. Acad. Sci. USA* **2019**, *116*, 22353-22358, doi:10.1073/pnas.1908662116.
55. Numata, A.; Takahashi, C.; Ito, Y.; Takada, T.; Kawai, K.; Usami, Y.; Matsumura, E.; Imachi, M.; Ito, T.; Hasegawa, T. Communesins, cytotoxic metabolites of a fungus isolated from a marine alga. *Tetrahedron Lett.* **1993**, *34*, 2355-2358, doi:10.1016/s0040-4039(00)77612-x.
56. Zhang, P.; Meng, L.-H.; Mándi, A.; Li, X.-M.; Kurtán, T.; Wang, B.-G. Structure, absolute configuration, and conformational study of resorcylic acid derivatives and related congeners from the fungus *Penicillium brocae*. *RSC Adv.* **2015**, *5*, 39870-39877, doi:10.1039/c5ra02203g.
57. Andersen, B.; Smedsgaard, J.; Frisvad, J.C. *Penicillium expansum*: Consistent production of patulin, chaetoglobosins, and other secondary metabolites in culture and their natural occurrence in fruit products. *J. Agr. Food Chem.* **2004**, *52*, 2421-2428, doi:10.1021/jf035406k.
58. Huang, S.; Chen, H.; Li, W.; Zhu, X.; Ding, W.; Li, C. Bioactive chaetoglobosins from the mangrove endophytic fungus *Penicillium chrysogenum*. *Mar. Drugs* **2016**, *14*, 172, doi:10.3390/md14100172.
59. Massias, M.; Rebuffat, S.; Molho, L.; Chiaroni, A.; Riche, C.; Bodo, B. Expansolides A and B: Tetracyclic sesquiterpene lactones from *Penicillium expansum*. *J. Am. Chem. Soc.* **1990**, *112*, 8112-8115, doi:10.1021/ja00178a039.
60. Huang, J.; She, J.; Yang, X.; Liu, J.; Zhou, X.; Yang, B. A new macrodiolide and two new polycyclic chromones from the fungus *Penicillium* sp. SCSIO041218. *Molecules* **2019**, *24*, 1686, doi:10.3390/molecules24091686.
61. Cheng, Z.; Xu, W.; Wang, Y.; Bai, S.; Liu, L.; Luo, Z.; Yuan, W.; Li, Q. Two new meroterpenoids and two new monoterpenoids from the deep sea-derived fungus *Penicillium* sp. YPGA11. *Fitoterapia* **2019**, *133*, 120-124, doi:10.1016/j.fitote.2018.12.022.
62. Matsuura, H.; Nakamori, K.; Omer, E.A.; Hatakeyama, C.; Yoshihara, T.; Ichihara, A. Three lasiodiplodins from *Lasiodiplodia theobromae* IFO 31059. *Phytochemistry* **1998**, *49*, 579-584, doi:10.1016/s0031-9422(98)00267-2.
63. Li, J.; Xue, Y.; Yuan, J.; Lu, Y.; Zhu, X.; Lin, Y.; Liu, L. Lasiodiplodins from mangrove endophytic fungus *Lasiodiplodia* sp. 318. *Nat. Prod. Res.* **2016**, *30*, 755-760, doi:10.1080/14786419.2015.1062762.
64. Pontius, A.; Krick, A.; Kehraus, S.; Brun, R.; Konig, G.M. Antiprotozoal activities of heterocyclic-substituted xanthones from the marine-derived fungus *Chaetomium* sp. *J. Nat. Prod.* **2008**, *71*, 1579-1584, doi:10.1021/np800294q.
65. Řezanka, T.; Jáchymová, J.; Dembitsky, V.M. Prenylated xanthone glucosides from Ural's lichen *Umbilicaria proboscidea*. *Phytochemistry* **2003**, *62*, 607-612, doi:10.1016/s0031-9422(02)00539-3.
